# Mesoporous Gold Nanospheres Confined Platinum Nanoclusters as Robust ROS and Oxygen Nanogenerators for NIR‐II Hyperthermia Cancer Therapy

**DOI:** 10.1002/advs.202502688

**Published:** 2025-05-11

**Authors:** Fei Cun, Jie Chen, Hanxue Li, Yufang Kou, Meiyan Wang, Xiaomin Li, Hui Chen, Jilie Kong

**Affiliations:** ^1^ Department of Chemistry Fudan University Shanghai 200438 China; ^2^ 411 Hospital, School of Medicine Shanghai University Shanghai 200444 China

**Keywords:** catalytic therapy, confinement effect, mesoporous gold, platinum nanozymes, photothermal therapy

## Abstract

While massive studies are focused on platinum (Pt)‐based nanozyme for antitumor therapies, their therapeutic efficiency is deficient due to the weak catalytic activity in the highly complex tumor microenvironment. Herein, mesoporous gold nanospheres confined platinum nanoclusters (MGNSs@Pt) as robust hydroxyl radical and oxygen nanogenerators are achieved for multimodal therapies. Benefiting from the confinement effect of the mesopores in the MGNSs, the Pt nanoclusters (Pt NCs) demonstrate enhanced stability and catalytic activity, with a catalytic constant (K_cat_) of 1.42 × 10^6^ s^−1^, which is 2 and 5 orders magnitude higher than K_cat_ values of Pt‐decorated non‐porous gold nanoparticles and pure Pt NCs respectively. Density functional theory (DFT) calculations reveal the proper interaction of intermediates contributes to the ultra‐high catalytic activity of MGNSs@Pt. Meanwhile, owing to the local surface plasmon resonance (LSPR) effect in the second near‐infrared (NIR‐II) bio‐window of MGNSs, the nanozymes exhibited high photothermal conversion efficiency up to 43.4%, which enhanced the nanocatalytic damage on cancer cells. This process can induce robust oxidative stress and oxygenation within the tumor, thereby activating the apoptosis pathway for tumor eradication by mitochondrial dysfunction, cell membrane disruption, HIF‐1α downregulation as well as caspase 3 activation, which pave the way for multimodal and effective cancer treatment.

## Graphical abstract

Mesoporous gold nanospheres confined platinum nanoclusters (MGNSs@Pt) is developed as robust hydroxyl radical and oxygen nanogenerators for multimodal therapies. Their NIR‐II LSPR property also allowed MGSNs to be ideal phototheranostic agents. The chemodynamic and photothermal therapy efficiently induced the tumor cell apoptosis, enabling the MGNSs@Pt nanocatalysts to exhibit an excellent tumor inhibition rate in 4T1‐tumor‐bearing mice.

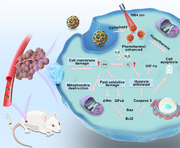

## 1 Introduction

Nanocatalytic therapy has emerged as a promising approach for tumor therapeutic.^[^ ^1^ ^]^ Among them, nanozymes enable the transformation or breakdown of intrinsic substances into toxic chemicals or the disruption of the internal equilibrium within the tumor microenvironment (TME).^[^ ^2^ ^]^ For instance, peroxidase (POD)‐mimicking nanozymes can decompose H_2_O_2_ into cytotoxic reactive species (ROS), resulting in tumor cell eradication.^[^ ^3^ ^]^ Meanwhile, the overexpressed H_2_O_2_ can also be catalytically converted into O_2_ by catalase (CAT)‐mimicking nanozymes, alleviating tumor hypoxia and curbing resistance to treatment, tumor aggressiveness, and metastasis.^[^ ^4^ ^]^ Despite the significant progress has been made on nanozyme‐based tumor therapy, challenges persist, such as relatively low catalytic efficiency and limited practical applications.

Up to now, platinum (Pt)‐based nanozymes have been extensively studied owing to their excellent characteristics in catalytic activity, biocompatibility and well‐controlled structure.^[^ ^5^ ^]^ Pt nanoclusters (NCs) were reported to possess higher peroxidase‐like activity than larger nanoparticles due to more active sites and higher affinity for H_2_O_2_.^[^ ^6^ ^]^ However, the small‐size NCs are hard to separate and tend to aggregate in solution, which induce the low stability and a sharp drop in catalytic efficiency owing to a serious decrease in active sites on nanoclusters’ surfaces. In order to avert the issue, one of the strategies is immobilizing the Pt NCs onto solid supports like MOFs,^[^ ^7^ ^]^ and carbon^[^ ^8^ ^]^‐based nanomaterials. However, these materials are microporous, which does not match the size of Pt NCs. This results in the most Pt NCs being located on the surface and constrains the stability. Other pore tunable material like mesoporous silica nanospheres are promising candidates for Pt NCs loading.^[^ ^9^ ^]^ However, they only served as nanocarriers with inactive surface that cannot enhance catalytic ability of Pt NCs. Therefore, designing and synthesizing solid supports with both high stability and enhancing catalytic ability to Pt NCs are crucial for peroxidase mimics.

Mesoporous metals with pore sizes of 2–50 nm are a new type of nanostructured nanomaterials.^[^ ^10^ ^]^ Efforts are underway to deposit additional metal clusters onto these mesoporous metals. The confined space from the mesopores can increase the absorption ability and retention time of reactant, which allows NCs to promote their catalytic efficiency.^[^ ^10^, ^11^ ^]^ The active metal site in mesoporous metal and NCs can provide the synergy effect, thus contributing to excellent catalytic activity.^[^ ^10c^ ^]^ Furthermore, the strong LSPR effect of mesoporous metals can enhance NCs’ photothermal effect, making their composite an idea photothermal agents for tumor therapy.^[^ ^12^ ^]^ Nevertheless, decorating NCs on mesoporous metals was rarely reported owing to the difficulty in accessing the internal pores and uneven distribution. Therefore, appropriate synthesis methods are crucial to achieve successful NCs loading on mesoporous metal.

Herein, 3D mesoporous gold nanospheres (MGNSs) confined Pt nanoclusters were synthesized for NIR‐II hyperthermia‐enhanced ROS and oxygen generation. The MGNSs not only served as the efficient nanocarriers to confine Pt NCs, but also contributed to significantly enhanced the catalytic ability of Pt NCs. Meanwhile, the NIR‐II LSPR and mesoporous property allowed MGSNs to be ideal phototheranostic agents with high photothermal conversion efficiency. These multiple modes of chemodynamic and photothermal therapy efficiently induced the tumor cell apoptosis, enabling the MGNSs@Pt nanocatalysts to exhibit an excellent tumor inhibition rate in 4T1‐tumor‐bearing mice. To best of our knowledge, this is the first time to report the confinement of noble metal nanoclusters in 3D mesoporous gold, offering promising catalytic and photothermal abilities for potential therapeutic applications.

## 2 Results and Discussion

### 2.1 Synthesis and Characterization of MGNSs@Pt

**Scheme 1:**
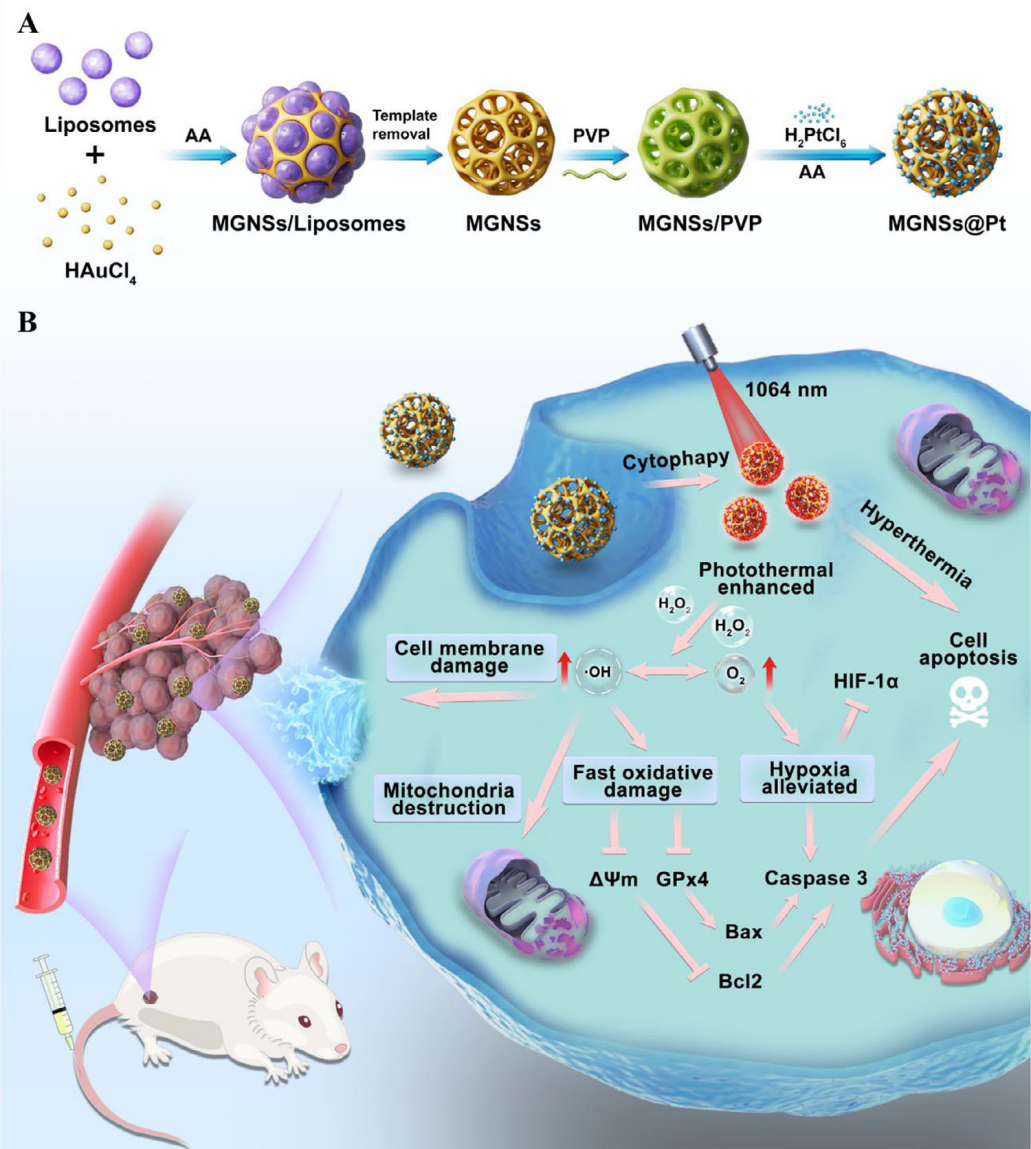
The synthesis A) and utilization B) of MGNSs@Pt nanomaterials for antitumor therapy through inducing hyperthermia‐enhanced ROS and oxygen generation.

**Figure 1:**
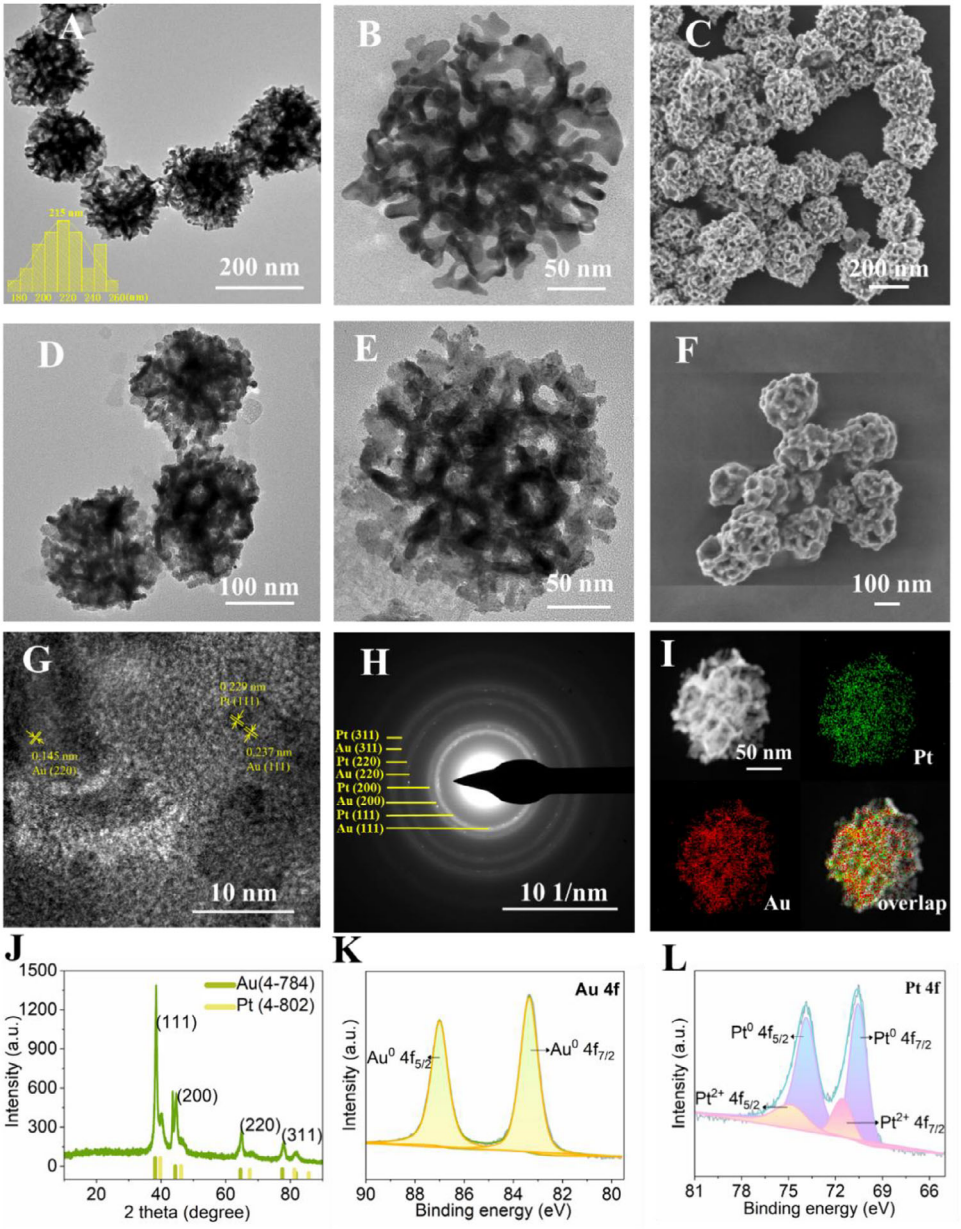
A,B) TEM images of MGNSs under different magnification. The insets in panels A are the size distribution of MGNSs. C) FESEM images of the MGNSs. D,E) TEM images of MGNSs@Pt (Pt content: 34 wt.%) under different magnification. F) FESEM images of the MGNSs@Pt. G) HRTEM images showing crystallinity of MGNSs@Pt. H) SAED patterns of MGNSs@Pt. I) Elemental mapping images of MGNSs@Pt. J) X‐ray diffraction (XRD) patterns of MGNSs@Pt. High‐resolution scans for K) Au 4f and L) Pt 4f spectra of MGNSs@Pt.

Inspired by the fascinating properties of mesoporous Au nanostructures and Pt NCs, MGNSs@Pt were finely designed and prepared as shown in **Scheme** **1**. First, liposomes were employed as the surfactant templates and ascorbic acid (AA) served as a reducing agent to generate interconnected mesopores in MGNSs (Figure S1, Supporting Information).^[^ ^13^ ^]^ Then, Pt nanoclusters were grown on the surface of MGNSs via the in‐situ growth strategy, where poly(vinylpyrrolidone) (PVP) was selected as a stabilizer and adsorbent reagent for PtCl_6_^2−^.^[^ ^14^ ^]^ By reacting with excessive ascorbic acid, the precursor (H_2_PtCl_6_) was ultimately reduced into Pt nanoclusters (see the Experimental Section for details). Transmission electron microscope (TEM) images showed that the as‐prepared monodispersed MGNSs were spherical in morphology (**Figure** **1**A,B). The mesoporous were distributed not only on the outer surface of MGNSs but also throughout the interior of the MGNSs. The average diameter of MGNSs was 210 nm measured by dynamic light scattering (Figure S2, Supporting Information). Field emission scanning electron microscopy (FESEM) images showed that the MGNSs had high dispersity and uniformity (Figure 1C). The mesoporous was evident in the FESEM images with the pore size of 20 to 48 nm. The pore size and interconnected pore structure throughout the entire nanoparticles gave MGNSs high surface area, which exposed abundant loading sites and created utmost large contact area for catalysts.

HRTEM (Figure 1D,E) and FESEM (Figure 1F) showed the representative morphology of as‐prepared MGNSs@Pt. The uniform spots and rough Au skeleton were very clear in TEM images compared to the bare MGNSs, indicating that the ultrasmall Pt nanoclusters were successively anchored on the mesoporous Au supports. The size of Pt nanoclusters was measured to be 2.4 ± 0.4 nm by randomly analyzing 100 particles (Figure S3, Supporting Information). HRTEM image with high magnification shows the lattice fringes of 0.145, 0.237, and 0.229 nm, which ascribed to the (220), (111) lattice plane of Au and (111) lattice plane of Pt (Figure 1G). Moreover, the samples showed a polycrystalline feature with a face‐centered cubic (fcc) crystal structure as revealed by the spotty rings of the selected area electron diffraction (SAED) pattern (Figure 1H). The MGNSs@Pt composites could be well indexed to the (111), (200), (220), and (311) lattice planes of Au and Pt, respectively. Energy dispersive spectroscopy (EDS) mapping further demonstrated the coexistence of Au and Pt elements in the sample, where Pt elements was uniformly distributed on the framework of MGNSs (Figure 1I). EDS analysis indicated Au/Pt atomic ratio was ≈68:32 (Figure S4, Supporting Information), which was consistent with the results of ≈34% Pt content in MGNSs@Pt sample analyzed by inductively coupled plasma optical emission spectroscopy (ICP‐OES) (Table S3, Supporting Information). The crystalline phase and the element chemical valence state of as‐prepared MGNSs@Pt were further measured by X‐ray diffraction (XRD, Figure 1J) and X‐ray photoelectron spectroscopy (XPS, Figure 1K,L; Figure S5, Supporting Information), which further confirmed the presence of Au and Pt elements in MGNSs@Pt. Moreover, zeta potential of these nanomaterials was shown in Figure S6 (Supporting Information). Such high‐content and ultra‐dispersed of Pt nanoclusters were successfully confined in MGNSs, making them ideal noble metal‐metal interfaces catalysts for anti‐tumor therapy.

### 2.2 The Photothermal Performance of MGNSs@Pt

**Figure 2:**
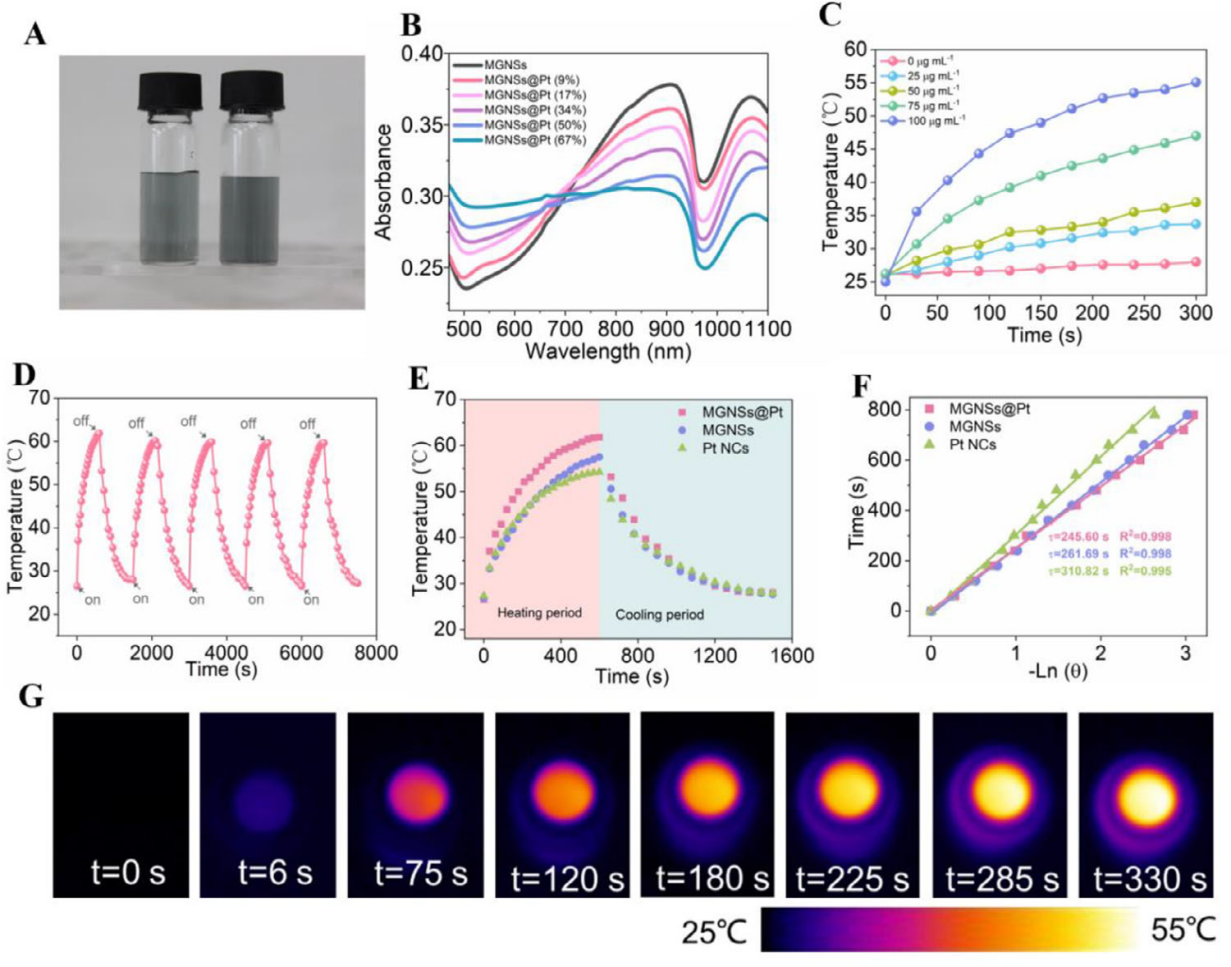
A) The digital picture of MGNSs (left) and MGNSs@Pt (right) solution. B) The absorption spectra of MGNSs and MGNSs@Pt with different Pt content. C) Concentration‐dependent photothermal curves of MGNSs@Pt under 1064 nm laser irradiation (2 W cm^−2^) for different times. D) Photothermal stability evaluation of MGNSs@Pt under five on‐off 1064 nm laser irradiation (100 µg mL^−1^, 2 W cm^−2^). E) Time‐resolved photothermal effect of MGNSs@Pt (Pt content: 34%), MGNSs and Pt NCs (100 µg mL^−1^, 2 W cm^−2^) with 1064 nm laser irradiation. F) The corresponding fitting curve of (E) based on Roper's method for photothermal conversion time constants calculation. G) Photothermal image of MGNSs@Pt under different time (0–330 s) exposed to a 1064 nm laser.

The color of MGNSs solution exhibited difference compared to conventional gold nanospheres or nanorods, which typically manifested dark green. With the deposition of platinum (Pt) nanoclusters, the color of the solution further intensified (**Figure** **2**A). Five types of MGNSs@Pt with different content of Pt (9, 17, 34, 50, and 67 wt.%) were synthesized by introducing different concentrations of H_2_PtCl_6_·6H_2_O solution, while kept other parameters constant (see more synthesis and characterization information in Figure S7 and Table S1, Supporting Information). Then, UV−vis spectroscopy was performed to reveal the connection between LSPR properties and MGNSs@Pt with different Pt content. In Figure 2B, the MGNSs solution had two LSPR peaks at 907 and 1066 nm. With the Pt content increased, the intensity of the adsorption peak was weakened. Compared to pure Pt NCs, an increase in absorbance at 1066 nm was observed upon the decoration of Pt NCs on MGNSs, showing an enhancement in the LSPR characteristics of Pt NCs. It was reported that the laser located in the NIR‐II bio‐window (1064 nm) offer the advantage of deeper tissue penetration, making them safer and more suitable compared to laser located in the NIR‐I biowindow at 808 nm.^[^ ^15^ ^]^ Figure 2C showed the photothermal performance of as‐prepared MGNSs@Pt (34 wt.%) by irradiation with a 1064 nm laser for 300 s. The heating behavior revealed concentration‐dependence pattern, where the temperature variations increased notably with higher concentrations of MGNSs@Pt. Then, the five heating/cooling cycles of MGNSs and MGNSs@Pt were conducted to investigate the photothermal stability (Figure 2D; Figure S8, Supporting Information). The trends of the heating/cooling curves were basically kept the consistent in MGNSs@Pt, with temperature differences during each thermal cycle being minimal, less than 1.5 °C, confirming its exceptional thermostability. The photothermal‐conversion efficiency of MGNSs@Pt, MGNSs and Pt NCs were determined based on Roper's method^[^ ^16^ ^]^ to be 43.4%, 30.5%, and 37.8% based on the time constant for heat transfer (Figure 2E,F). Notably, the photothermal conversion performance of MGNSs@Pt was improved. This enhancement could be attributed to the synergy effect of MGNSs@Pt, which lead to an efficient light‐harvesting in NIR‐II window, thereby promoted the generation of heat and the photothermal conversion. The photothermal conversion efficiency of MGNSs@Pt was greatly surpassed the reported efficiencies of various Au or Pt based noble metal nanomaterials (Table S4, Supporting Information). The high photothermal‐conversion efficiency was due to numerous nanopores and edges generated in MGNSs@Pt, which effectively amplify the electromagnetic field and facilitate a greater number of hot electrons produced.^[^ ^17^ ^]^ Photothermal imaging also demonstrated the substantial temperature elevation in the MGNSs@Pt dispersion, with the temperature increasing from 25.0 °C to 53.1 °C (Figure 2G), and the temperature was sufficient to kill tumor cells.

### 2.3 Nanocatalytic Performance of MGNSs@Pt

**Figure 3:**
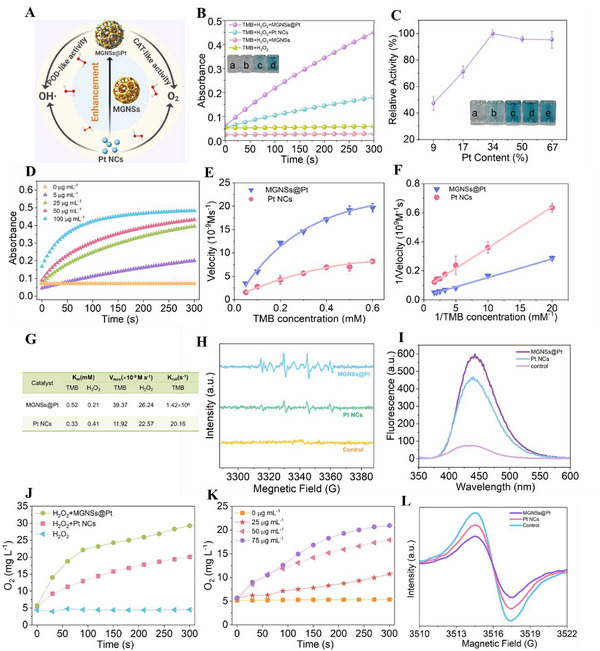
A) Schematic illustration for the catalytic performance of MGNSs@Pt. B) Absorbance curves at 650 nm with different catalysts. Inserts: the color change of (a) TMB+H_2_O_2_, (b) TMB+H_2_O_2_+MGNSs, (c) TMB+H_2_O_2_+Pt NCs, and (d) TMB+H_2_O_2_+MGNSs@Pt. C) The relative POD‐like activity of MGNSs@Pt with different Pt NCs content. Inserts: the color change corresponding to MGNSs@Pt with different Pt NCs content (9, 17, 34, 50, and 67 wt.%). D) Absorbance curves at 650 nm with different concentrations of MGNSs@Pt (0, 5, 25, 50, and 100 µg mL^−1^). E) Steady‐state kinetic assay of MGNSs@Pt and Pt NCs by varying concentrations of TMB. F) Double‐reciprocal plots of (E). G) Comparison of the Michaelis−Menten constant (K_m_) and the Maximum Reaction Velocity (V_max_) among MGNSs@Pt and Pt NCs. H) ESR spectra of 50 mm DMPO, 50 mm H_2_O_2_ with MGNSs@Pt and Pt NCs (100 µg mL^−1^) at pH 6.5. I) Fluorescence spectra for the system of 0.5 mm TA, 50 mm H_2_O_2_ with MGNSs@Pt and Pt NCs (100 µg mL^−1^) at pH 6.5. J) O_2_ generated by MGNSs@Pt and Pt NCs (100 µg mL^−1^) with H_2_O_2_ (10 mm) addition. K) O_2_ generated by MGNSs@Pt at different concentrations (0, 25, 50, and 75 µg mL^−1^) with H_2_O_2_ (10 mm) addition. L) ESR spectra of 0.1 mm ^15^N‐PDT mixed with MGNSs@Pt and Pt NCs (75 µg mL^−1^) in 0.2 mm H_2_O_2_ at pH 6.5.

Pt nanozymes have been reported to have pH‐switchable enzyme‐mimetic properties, including both intrinsic peroxidase (POD)‐like and catalase (CAT)‐like activities (**Figure** **3**A). The POD‐like activity of Pt NCs involves the decomposition of H_2_O_2_ into ·OH at lower pH condition, whereas the CAT‐like activity results in the decomposition of H_2_O_2_ into O_2_, with a preference for occurrence at higher pH levels.^[^ ^18^ ^]^ In TME, Pt NCs showed two enzyme‐mimetic properties producing ·OH and O_2_.^[^ ^19^ ^]^ Accordingly, we applied a typical catalytic oxidation reaction containing a 3,3′,5,5′‐tetramethylbenzidine (TMB, a chromogenic substrate) and H_2_O_2_ to study POD‐like catalytic property of MGNSs@Pt in the slight acidic TME (pH = 6.5).^[^ ^15^, ^20^ ^]^ As shown in Figure S9 (Supporting Information), a blue‐colored product (oxTMB) with a maximum adsorption at 650 nm was yielded after adding MGNSs@Pt into the substrate. In the meantime, the absorbance with different catalysts was observed in Figure 3B. Evidently, the catalytic rate of MGNSs@Pt surpassed that of Pt NCs. It was observed that MGNSs could not catalyze the TMB, indicating that the catalytic behaviors predominantly originated from the Pt nanoclusters. To optimize the catalytic efficiency, the POD‐like activity of MGNSs@Pt with different Pt content (9, 17, 34, 50, and 67 wt.%) were evaluated (Figure 3C). As expected, the catalytic oxidation abilities improved with the Pt content up to 34 wt.%. However, beyond 34 wt.%, catalytic activity slightly decreased. The phenomenon could be explained by observing their morphology in TEM images (Figures S7 Supporting Information). Initially, low Pt content enabled MGNSs to have abundant loading capacity and active sites. However, loading areas reached saturation as the amount of Pt reached a threshold, resulting in the accumulation of Pt nanoclusters and the block of active sites, which impeded the transport of reactants. Thus, peroxidase‐like activity was limited beyond 34 wt.%. Since MGNSs@Pt with 34 wt.% Pt NCs content had highest catalytic efficiency, it was chosen for subsequent studies.

Figure 3D showed the concentration‐dependent manner in POD‐like activity of MGNSs@Pt. After investigating the effect on peroxidase‐like activity under different condition (Figure S10, Supporting Information), the steady‐state kinetic parameters were further measured. Michealis‐Menten curves and double‐reciprocal Lineweaver−Burk plot for the reaction between TMB and H_2_O_2_ was presented in Figure 3E,F and Figure S11 (Supporting Information).^[^ ^21^ ^]^ The steady‐state kinetic parameters were calculated and summarized in Figure 3G. Notably, MGNSs@Pt exhibited the highest V_max_, with calculated values 3.3 times higher than those Pt NCs, respectively. For a more precise quantification of the catalytic efficiency of these nanozymes, catalytic constants (K_cat_) were determined from the equation [K_cat_ = V_max_/E], with E (particle concentration) was measured via nanoparticle tracking analysis (Figure S12, Supporting Information). Remarkably, the K_cat_ value of MGNSs@Pt reached an impressive value of 1.42 × 10^6^ s^−1^. In comparison to various reported Au, Pt, or Au‐Pt composite nanoparticles, MGNSs@Pt exhibited the highest POD‐like catalytic efficiency, surpassing these metal nanomaterials by ≈1–3 orders of magnitude (Table S5, Supporting Information). Present studies had demonstrated that the hydroxyl radical (·OH) was one of the most common ROS produced in H_2_O_2_‐TMB catalytic systems.^[^ ^22^ ^]^ Hence the electron spin resonance (ESR) spectroscopy was applied to investigate ·OH radicals generation using 5,5‐dimethyl‐1‐pyrroline N‐oxide (DMPO) as the spin trap. As shown in Figure 3H, higher characteristic peaks (intensity ratio was 1:2:2:1) were generated than pure Pt NCs, indicating that the POD‐like process in MGNSs@Pt involved elevated ·OH generation. Terephthalic acid (TA) and isopropanol as ·OH radicals probe also showed that MGNSs@Pt displayed the stronger ·OH generation than Pt NCs (Figure 3I; Figure S13, Supporting Information).

The CAT‐like activity enabled nanozymes to decompose H_2_O_2_ to generate O_2_, which is a crucial process for alleviating hypoxia within the TME with high H_2_O_2_ levels.^[^ ^3^, ^23^ ^]^ The catalase‐like activity of MGNSs@Pt was assessed by real‐time detection of O_2_ in an H_2_O_2_ solution using a portable oxygen meter. As shown in Figure 3J,K, MGNSs@Pt exhibited superior catalase‐like activity compared to Pt NCs. At the concentration of MGNSs@Pt at 75 µg mL^−1^, the O_2_ content was increased to the highest level at 300 s, indicating the robust CAT‐like activity of MGNSs@Pt. To further prove the existence of O_2_, ESR oximetry was applied to investigate CAT‐like process.^[^ ^24^ ^]^ As shown in Figure 3L, MGNSs@Pt displayed a relatively low peak intensity compared to Pt NCs into ^15^N‐PDT+H_2_O_2_ system, suggesting that more O_2_ was produced from H_2_O_2_ decomposition and superior CAT activity. Meanwhile, different storage condition was evaluated and the activity of MGNSs@Pt solution still maintained 84% after a month (pH = 6.5, 4 °C, Figure S14, Supporting Information).

In addition to pure Pt NCs, gold nanorods (non‐porous gold particles) decorated with Pt NCs (GNRs@Pt) were also prepared to validate the influence of porosity on the POD‐like activity in MGNSs (Figures S15 and S16, Supporting Information). The Au and Pt contents in GNRs and MGNSs were generally kept the same (Table S3, Supporting Information). Figures S17 and S18 (Supporting Information) illustrated that the POD‐like activity of MGNSs@Pt was higher than GNRs@Pt.

### 2.4 Mechanism of Enhanced POD‐Like Activity and CAT‐Like Activity

**Figure 4:**
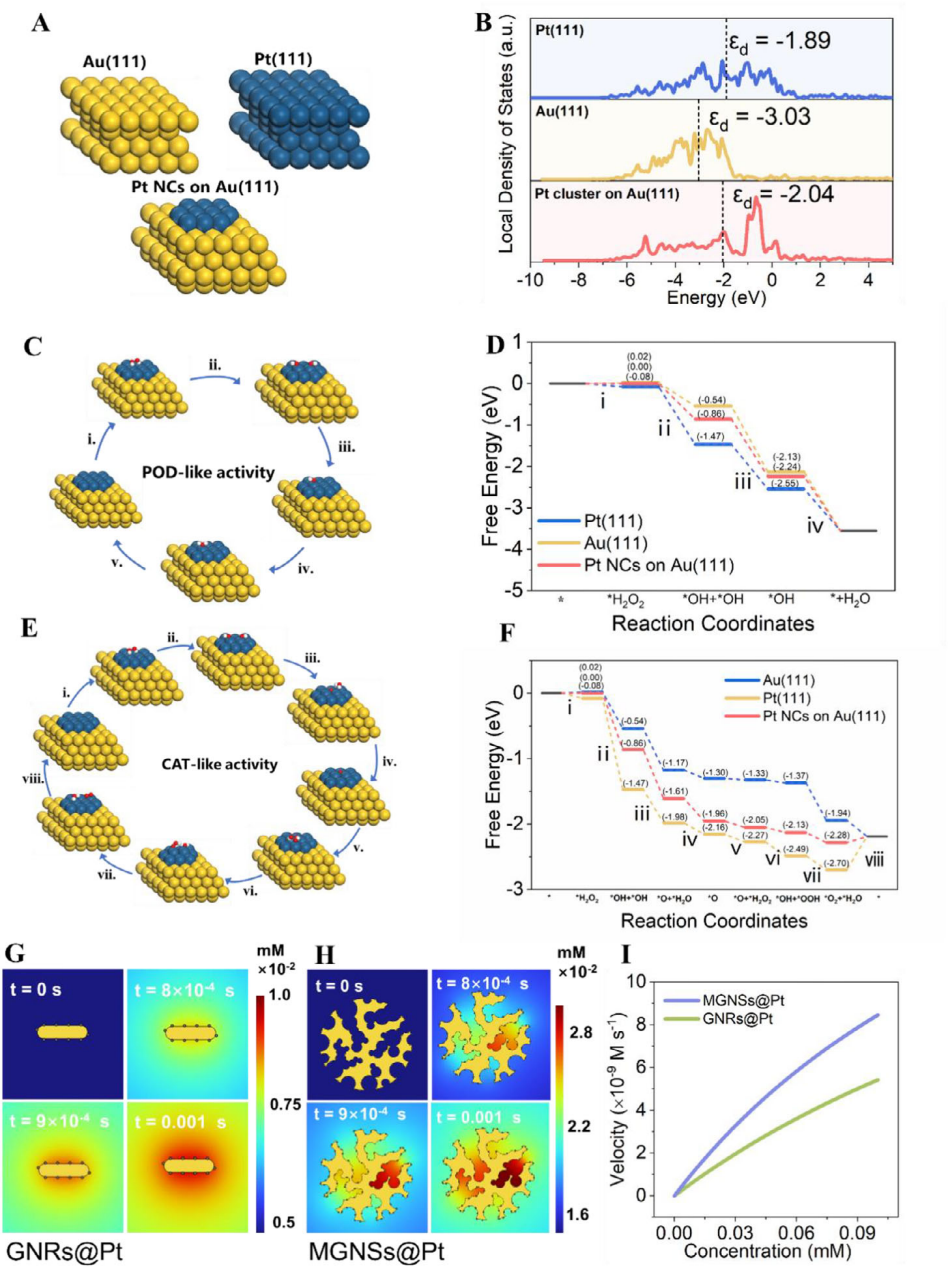
A) The stable configuration of MGNSs@Pt (Pt cluster on Au (111)), MGNSs (Au (111)), and Pt NCs (Pt (111)) based on the principle of energy minimization. B) The calculated local DOS of Pt NCs, MGNSs, and MGNSs@Pt, where the corresponding d‐band centers (ε_d_) are labeled with black dash lines, and the Fermi level is set to be zero. C) The proposed catalytic mechanism of POD‐like activity. D) The calculated free energy diagram POD‐like reaction on the Pt(111) surface, Au(111) surface, and Pt cluster on Au(111). E) The proposed catalytic mechanism of CAT‐like activity. F) The calculated free energy diagram CAT‐like reaction on the Pt(111) surface, Au(111) surface, and Pt cluster on Au(111). G) Real‐time ·OH generation of constructed GNRs@Pt models with initial H_2_O_2_ concentration of 0.1 mm. H) Real‐time ·OH generation of constructed MGNSs@Pt models with initial H_2_O_2_ concentration of 0.1 mm. I) The simulated velocity of ·OH generation in MGNSs@Pt and GNRs@Pt by varying concentrations of H_2_O_2_.

Kinetic data revealed that Pt NCs combined with MGNSs exhibited enhanced catalytic rates compared to their pure counterparts. These results provide clear evidence of the catalytic activity enhancement facilitated by the mesoporous gold structures on Pt NCs. To gain more insights of its enhanced catalytic ability in MGNSs, we performed density functional theory (DFT) calculations for H_2_O_2_ decomposition using three simplified catalyst models. As shown in **Figure** **4**A, Au(111) and Pt(111) was established to simulate the surface of prepared MGNSs and Pt NCs. In order to achieve stable structures for MGNSs@Pt, the configuration with minimized energy was designed by anchoring 10 Pt atoms as mimicking Pt NCs(111) on Au(111) slab according to HRTEM images in Figure 1G. The three models of optimized atomic structures of adsorbed intermediates during catalytic reaction on surface atoms are shown in Figures S19 and S20 (Supporting Information). Studies have well demonstrated that density of state (DOS) can reflect the electrons distribution of transition mentals in the orbitals, thereby predicting the origin of the binding affinity between adsorbates and catalysts. Figure 4B shows the DOS profiles of Pt NCs, MGNSs, and MGNSs@Pt. According to the Sabatier principle, the adsorption strength in an intermediate level would contribute to an optimal catalysis effect.^[^ ^25^ ^]^ Pt NCs with a dominant occupation close to the Fermi level (0 eV) implied a strong binding with reaction species, which was unfavorable due to a slow transition for stable intermediates. The catalytic rate was also adversely affected on the surface of MGNSs because a weak interaction occurred which leaded to a low population of intermediates. Altogether, MGNSs@Pt exhibited optimal performance due to its appropriate adsorption strength during the catalytic process.

Further, the free energy diagram for reaction pathways was calculated to understand the POD‐like activity and CAT‐like activity. For POD‐like activity, the catalysis was initiated by a H_2_O_2_ molecule adsorption at the surface of Pt site (i). Then, *H_2_O_2_ were dissociated to give a dual hydroxyl‐adsorbed structure (ii). The *OH was subsequently hydrolyzed by amino groups of two TMB‐H^+^ and formed two H_2_O molecules (iii and iv) (Figure 4C).^[^ ^2b^ ^]^ In Figure 4D, Pt NCs possessed the most negative free energy of *H_2_O_2_ (−0.08 eV) and *OH+*OH (−1.47 eV), indicating the favorable adsorption and dissociation of H_2_O_2_ on surfaces of Pt NCs. However, the desorption energy of *OH species (−1.47 to −2.55 eV and −2.55 to −3.55 eV) was lower than that of MGNSs and MGNSs@Pt, which suggested the strong adsorption of *OH and the difficulty to be removed by amino of TMB on Pt NCs surface. In contrast, the weakest adsorption occurred on MGNSs slab (0.02 eV for *H_2_O_2_, –0.54 eV for *2OH and −2.13 eV for *OH), where dissociation of H_2_O_2_ was limited primarily, but *OH hydrogenation was barrierless. It is noteworthy that MGNSs@Pt had the proper interaction strength that could balance the two key reaction steps. There is no high energy barrier in *H_2_O_2_ decomposition and *OH desorption, which resulted in *OH adsorption and transfer. Thus MGNSs@Pt was the most catalytically active. The CAT‐like activity process was demonstrated in the reaction step according to the previous reports (Figure 4E).^[^ ^3^, ^4^ ^]^ The adsorbed *H_2_O_2_ underwent the cleavage into *OH (i and ii), followed by the formation of *O and *H_2_O (iii). After the desorption of *H_2_O (iv), *O combined with another *H_2_O_2_ molecules to generate *OH and *OOH (v and vi). Ultimately, *OH reacted with *OOH to yield *O_2_ and *H_2_O, and released from the catalysis (vii and viii). A similar adsorption trend was also observed for the active species during CAT‐like activity. In the energy profiles presented in Figure 4F, the adsorption energy of H_2_O_2_ on Pt NCs slab (−0.08 eV) was lower than that of MGNSs (0.02 eV) and MGNSs@Pt (0.00 eV), indicating the H_2_O_2_ molecules exhibited a higher affinity for adsorption on the surface of Pt NCs. And Pt NCs were more conductive to cleave H_2_O_2_ into *OH species homogeneously since it was a spontaneous exothermic reaction with the lowest adsorption energy of −1.47 eV (*OH + *OH). However, the subsequent step of *O formation was the least exothermic (−1.47 to −1.98 eV), indicating the difficulty in removing *OH from the Pt NCs. Moreover, Pt NCs returned to the initial state with the desorption of *H_2_O and *O_2_ (viii) was observed to be an endcothermic reaction demanding an energy injection of 0.51 eV, thus presenting a substantial energy barrier to overcome. In contrast, the free energy of *O_2_ formation from *OH+*OOH (−1.37 to −1.94 eV) and desorption of H_2_O and O_2_ (–1.94 to –2.19 eV) was highly exothermic on MGNSs slab, which indicated that O_2_ was easy to form and release from the MGNSs. However, the cleavage of *H_2_O_2_ into *OH + *OH (0.02 to –0.54 eV) was the least endothermic, indicating the challenge in dissociating *H_2_O_2_. Similarly, MGNSs@Pt displayed the proper adsorption strength for active species, where the interaction between MGNSs and Pt NCs played a crucial role in the enhancement of CAT‐like activity.

To prove the confinement structure, the concentration of H_2_O_2_ and ·OH of MGNSs@Pt and GNRs@Pt were both simulated with finite element method (FEM) according to previous reports.^[^ ^26^ ^]^ The cross section of MGNSs@Pt and GNRs@Pt was built as 2D simulation models to imitate real‐time POD‐like process and the corresponding model parameters were listed in Table S6. H_2_O_2_ consumption gradually increased along with time, especially within the inner pores of MGNSs@Pt, and displayed a higher consumption rate compared to GNRs@Pt (Figures S21 and S22, Supporting Information). As shown in Figure 4G,H, compared to GNRs@Pt, the MGNSs@Pt generated more ·OH within the mesopores due to the nanoconfined evironment. According to the simulation results, the average generation rate of ·OH in the computational domain of MGNSs@Pt was higher than GNSs@Pt at various H_2_O_2_ concentration, which was in very good agreement with experimental results (Figure 4I; Figure S11A, Supporting Information). Collectively, the remarkable catalytic activity of MGNSs@Pt was attributed to two factors: 1) the MGNSs provided the appropriate adsorption ability for active species; 2) the mesoporous framework of MGNSs created the confinement effect for Pt NCs inside the mesopores.

### 2.5 In Vitro Antitumor Effect of MGNSs@Pt

**Figure 5:**
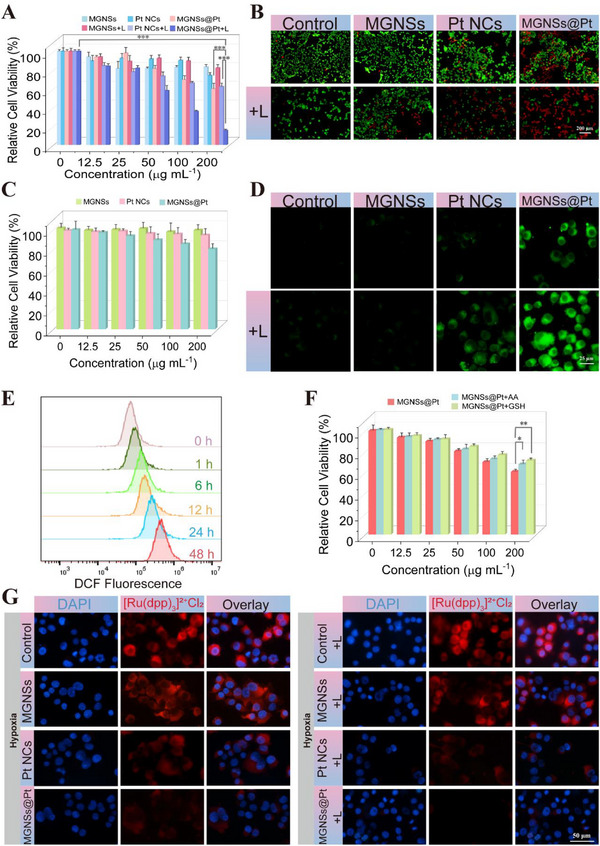
A) 4T1 cell viabilities after treatment with various concentrations (0, 12.5, 25, 50, 100, and 200 ug mL^−1^) of MGNSs, Pt NCs, MGNSs@Pt with (indicated with + L) or without 1064 nm laser irradiation. B) Live/dead staining of 4T1 cells treated with MGNSs, Pt NCs, MGNSs@Pt (200 µg mL^−1^) with or without 1064 nm laser irradiation. C) HUVEC cell viabilities after treatment with various concentrations (0, 12.5, 25, 50, 100, and 200 ug mL^−1^) of MGNSs, Pt NCs, MGNSs@Pt. D) Confocal laser scanning microscopy (CLSM) images of ROS in 4T1 cells treated with MGNSs, Pt NCs, MGNSs@Pt (100 µg mL^−1^) with or without 1064 nm laser irradiation. E) Flow cytometric analysis of intracellular ROS levels in 4T1 cells after treatment with MGNSs@Pt (100 µg mL^−1^) for 1, 6, 12, 24, and 48 h with 1064 nm laser irradiation. F) 4T1 cell viabilities after treatment with different concentrations of MGNSs@Pt, MGNSs@Pt+AA, or MGNSs@Pt+GSH. AA concentration: 100 µmol L^−1^, GSH concentration: 20 mmol L^−1^. G) CLSM image of intracellular O_2_ using [Ru(dpp)_3_]^2+^Cl_2_ as a probe after the treatments of MGNSs, Pt NCs, MGNSs@Pt (100 µg mL^−1^) with or without 1064 nm laser irradiation (low‐oxygen conditions: 5% CO_2_, 1% O_2_, and 94% N_2_). Data are defined as mean ± SD (*n* = 3). Statistical significance is assessed by unpaired Student's two‐sided *t*‐test and asterisks indicate significant differences (*p < 0.05, **p < 0.01, and ***p < 0.001).

The overexpressed hydrogen peroxide and week acidic condition in TME provided accessibility for nanozymes to act as cytotoxic agents against cancer cell by ROS generation. Meanwhile, nanozymes could overcome the intrinsic hypoxia within TME by decomposition of hydrogen peroxide into oxygen, which improved tumor‐specific therapeutic outcomes.^[^ ^23^, ^27^ ^]^ Given the promising performance of MGNSs@Pt, we employed them as chemodynamic agents and photothermal conversion agents for cancer therapy. Initially, the CCK‐8 assay was performed to access the cytotoxicity of MGNSs, Pt NCs and MGNSs@Pt on the mouse breast cancer 4T1 cells under the dark and laser irradiation conditions. The results showed that MGNSs@Pt had an obvious anti‐tumor effect on cancer 4T1 cells with the cell viability decreased to 59.9% at the concentration of 200 µg mL^−1^ (**Figure** **5**A) compared to MGNSs and Pt NCs with the cell viability of 83.2% and 74.3%. Moreover, the viability of 4T1 cells reduced when the MGNSs@Pt concentration increased, which mainly attributed to their oxidative injury, triggering a sufficiently high level of toxic ·OH and O_2_ to kill 4T1 cells. Further, the cytotoxic effect was significantly increased upon exposure to a 1064 nm laser. MGNSs@Pt plus laser irradiation (MGNSs@Pt + L group) exhibited maximum cytotoxicity toward 4T1 cells with 15.3% survival rate compared to Pt NCs plus laser irradiation (Pt NCs + L group) with 62.9% survival rate at a dose of 200 µg mL^−1^. These results demonstrated MGNSs@Pt have a potential for combining catalytic therapy and PTT. MGNSs not only serve as a nanocarrier for Pt NCs but also play a critical role in enhancing the overall therapeutic effect of the system. Next, live/dead cell double staining was used to verify the toxicity of MGNSs@Pt to 4T1 cells using calcein AM and propidium iodide (PI). MGNSs@Pt‐treated 4T1 cells exhibited strong red fluorescence under laser irradiation due to PI staining of the dead cells (Figure 5B; Figure S23A, Supporting Information) compared to three control groups. Notably, MGNSs@Pt showed little cytotoxic effect on the normal cell lines (HUVECs), especially at high concentration (Figure 5C). These results indicated that MGNSs@Pt has excellent biosafety and biocompatibility. The conversion of the overexpressed H_2_O_2_ in 4T1 cells into highly cytotoxic chemicals was the key factor for tumor‐specific therapy.

The intracellular ·OH generation capacity was further assessed using the ROS fluorescence probe 2′,7′‐dichlorofluorescin diacetate (DCFH‐DA), which could convert into 2′,7′‐dichlorofluorescein (DCF) with strong green fluorescence (λ_ex_ = 488 nm; λ_em_ = 517 nm) in the presence of ROS.^[^ ^28^ ^]^ In Figure 5D and Figure S23B (Supporting Information), 4T1 cancer cells exhibited strong green fluorescence signal following a 4 h incubation with MGNSs@Pt, whereas low fluorescent signal intensity was observed in the control, MGNSs and Pt NCs, indicating the enhanced catalytic effects of nanocatalysts in tumor cell. Moreover, a 1064 nm laser irradiation improved ROS generation in both Pt NCs and MGNSs@Pt treated 4T1 cells, suggesting that the promoting capability of the ROS generation in hyperthermia from the photothermal effect. In addition, the time‐dependent DCF fluorescence intensity was observed in MGNSs@Pt + L group (Figure 5E), indicating the accumulation of ROS with the incubation time prolonged. To evaluate the impacts of MGNSs@Pt on growth inhibition of tumor cell by oxidation, MGNSs@Pt were co‐treated with two different antioxidant agents (L‐ascorbic acid (AA) and glutathione (GSH)). The results showed that cell viability was increased in the cells treated with MGNSs@Pt and AA, or GSH, suggesting that AA and GSH could consume the oxidative ROS, which was a crucial factor in the cytotoxicity of MGNSs@Pt towards tumor cells (Figure 5F). These results were consistent with the ESR data that MGNSs@Pt induced the massive ROS production both in the solutions and intracellular environment.

We also proposed that the CAT‐like ability of MGNSs@Pt would generate O_2_ by decomposing the endogenous H_2_O_2_ in tumor cells to reverse the hypoxia and improve the antitumor effect. To confirm the self‐supplying O_2_ capacity of MGNSs@Pt in 4T1 cells, the fluorescent indicator [Ru(dpp)_3_]^2+^Cl_2_, which could be quenched by O_2_, was utilized to assess the intracellular O_2_ level in a hypoxic environment.^[^ ^19a^ ^]^ As shown in Figure 5G and Figure S23C (Supporting Information), the MGNSs@Pt group showed a weaker red fluorescence signal compared to the Pt NCs, MGNSs and control groups, and in the lower fluorescence intensities was observed with the laser irradiation condition. These results demonstrated that the trend in catalytic activity for O_2_ generation was consistent with ·OH generation. Together, the MGNSs@Pt + L group displayed superiority in CAT‐like activity and POD‐like activity over the Pt NCs and MGNSs groups, making it a promising nanoplatform for synergistic treatment of cancer.

### 2.6 Mechanism of MGNSs@Pt‐Induced Tumor Cell Death

**Figure 6:**
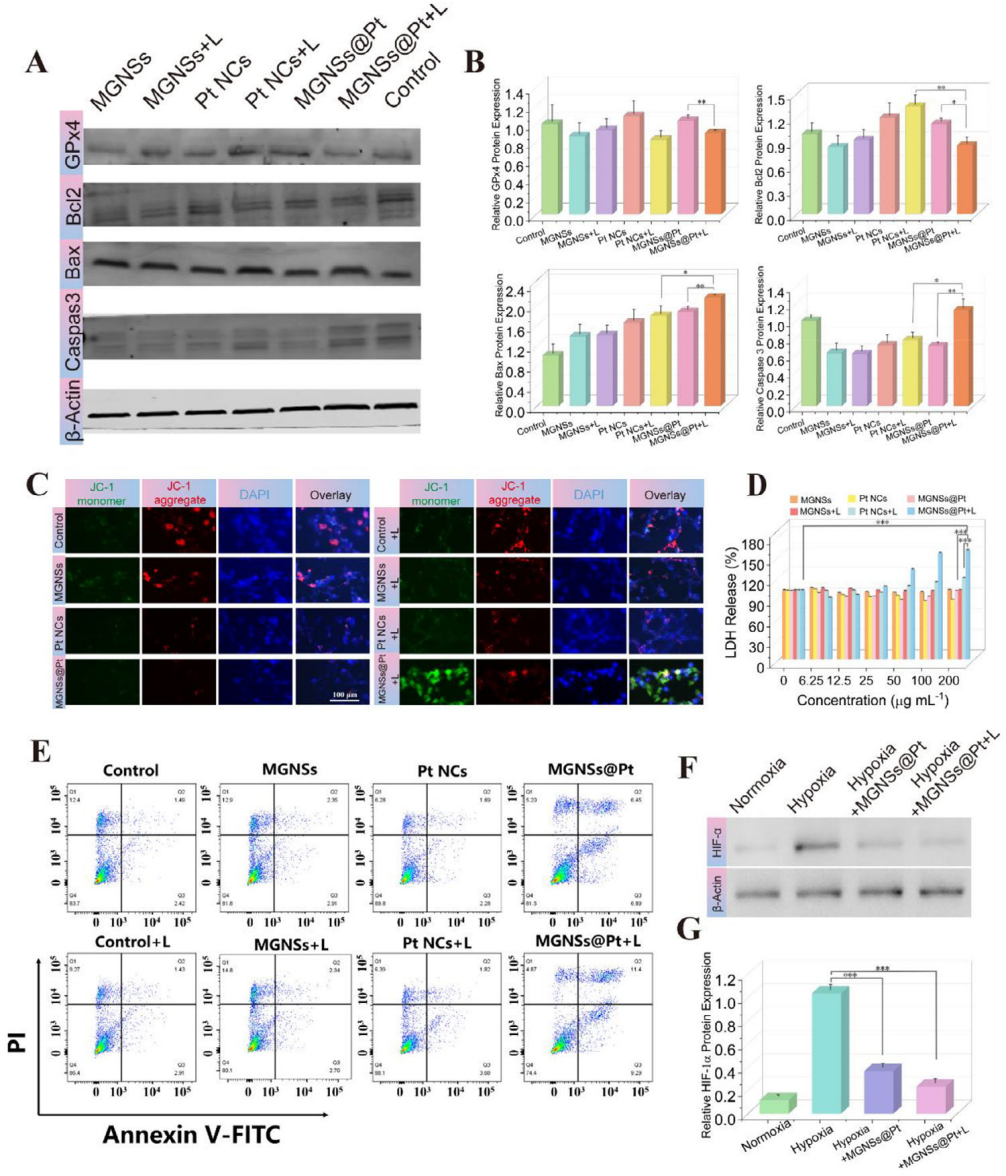
A) Western blot analysis of the protein expression of Caspase 3, GPx4, Bcl2, and Bax in 4T1 cells after different treatments. Nanomaterial concentration: 100 µg mL^−1^, laser irradiation condition: 2 W cm^−2^, 5 min. B) The corresponding quantitative analysis of the protein expression of (A) (*n* = 3). C) JC‐1 staining of 4T1 cells after treatments with MGNSs, Pt NCs, MGNSs@Pt with or without 1064 nm laser irradiation. Nanomaterial concentration: 100 µg mL^−1^, irradiation condition: 2 W cm^−2^, 5 min. D) LDH release of 4T1 cells after treatments with MGNSs, Pt NCs, MGNSs@Pt with or without 1064 nm laser irradiation. Nanomaterial concentration: 100 µg mL^−1^, irradiation condition: 2 W cm^−2^, 5 min. E) Flow cytometry analysis of 4T1 cells apoptosis after treatments with MGNSs, Pt NCs, MGNSs@Pt with or without 1064 nm laser irradiation. Nanomaterial concentration: 100 µg mL^−1^, irradiation condition: 2 W cm^−2^, 5 min. F) Western blot analysis of the protein expression of HIF‐1α after different treatments. Nanomaterial concentration: 100 µg mL^−1^, irradiation condition: 2 W cm^−2^, 5 min. G) The corresponding quantitative evaluation of HIF‐1α expression of (F). Data are defined as mean ± SD (*n* = 3). Statistical significance is assessed by unpaired Student's two‐sided *t*‐test and asterisks indicate significant differences (*p < 0.05, **p < 0.01, and ***p < 0.001).

The combined contribution from ROS‐induced oxidative damage and oxygen release influenced the relevant protein expression in tumor cells. To understand the mechanisms of MGNSs@Pt‐induced cell death, western blot experiments were performed to access the antioxidant system‐related protein (GPx4) expression and apoptosis‐related protein (Bax, Bcl2, and Caspase 3).^[^ ^2^, ^29^ ^]^ The results demonstrated a downregulation of GPx4 protein expression by 23.8% and 13.3% in the Pt NCs and MGNSs@Pt groups with laser irradiation, respectively, compared to the non‐laser‐irradiated cell groups. This downregulation in expression indicated laser irradiation enhanced the oxidative stress within 4T1 cells (**Figure** **6**A,B). Moreover, the MGNSs@Pt with PTT therapy decreased the intracellular content of Bcl2 by 15.1%, and upregulated Bax by 130% compared to the non‐treated group (control group). In addition, the level of activated caspase 3 was increased 12.1% in MGNSs@Pt + L group compared to the non‐treated group (control group). These results revealed that MGNSs@Pt with laser irradiation potentially promote the cell death by regulating apoptosis‐related proteins. The ratio of Bax to Bcl was correlated with mitochondria dysfunction.^[^ ^2^, ^30^ ^]^ Tetrechloro‐tetraethyl benzimidazol carbocyanine iodide (JC‐1, a cationic dye) was utilized to measure mitochondrial membrane potential, which exhibited potential‐dependent accumulation in mitochondria with red fluorescence. As shown in Figure 6C and Figure S24A (Supporting Information), the damage to the mitochondrial membrane induced by MGNSs@Pt was verified through the decrease of red fluorescent signals, which could be explained by the oxidative damage resulting from ·OH. Moreover, the highest green fluorescence intensity was observed in the MGNSs@Pt + L group, suggesting the laser irradiation intensified the serious mitochondria damage. Subsequently, cellular membrane integrity was assessed to investigate cell death using the lactate dehydrogenase (LDH) quantification assay.^[^ ^31^ ^]^ The MGNSs@Pt + L group showed the elevated LDH activity with increasing concentrations of MGNSs@Pt, resulting in a substantial release of up to 158.5% (Figure 6D). Further, the cell death mechanism was verified by fluorescence‐activated cell sorting (FACS) with Annexin V‐FITC and PI staining (Figure 6E; Figure S24B, Supporting Information). The percentage of cell apoptosis in the MGNSs@Pt group (6.45%) was higher than that in the control (1.49%), MGNSs (2.35%) and Pt NCs (1.69%) groups. In particular, the MGNSs@Pt + L group showed a higher percentage of cell apoptosis (11.4%), which was attributed to the synergistic effects of PTT and hyperthermia‐enhanced catalytic therapy.

As reported previously,^[^ ^3^, ^23^ ^]^ the cellular response to hypoxia has been shown to be primarily regulated by hypoxia inducible factor (HIF)‐1α. As shown in Figure 6F,G, HIF‐1α expression were markedly upregulated in 4T1 cells under the hypoxic stress compared to that in the normoxia condition. However, HIF‐1α was downregulated after treatment with MGNSs@Pt and MGNSs@Pt + L. Therefore, MGNSs@Pt plus 1064 nm laser irradiation has the potential to break through tumor hypoxia limitation to promote the effectiveness of tumor treatment. These protein expression in 4T1 cells by MGNSs@Pt + L treatment provided feedback for comprehending the therapy mechanism and guidance for improving tumor therapy. These data indicated that MGNSs@Pt with a 1064 nm laser irradiation significantly increased ·OH generation and O_2_ content, inducing oxidative damage and hypoxia alleviation within tumor cells. The interplay of mitochondrial dysfunction, cell membrane disruption, HIF‐1α downregulation as well as caspase 3 activation leaded to the effective induction of tumor cells apoptosis.

**Figure 7:**
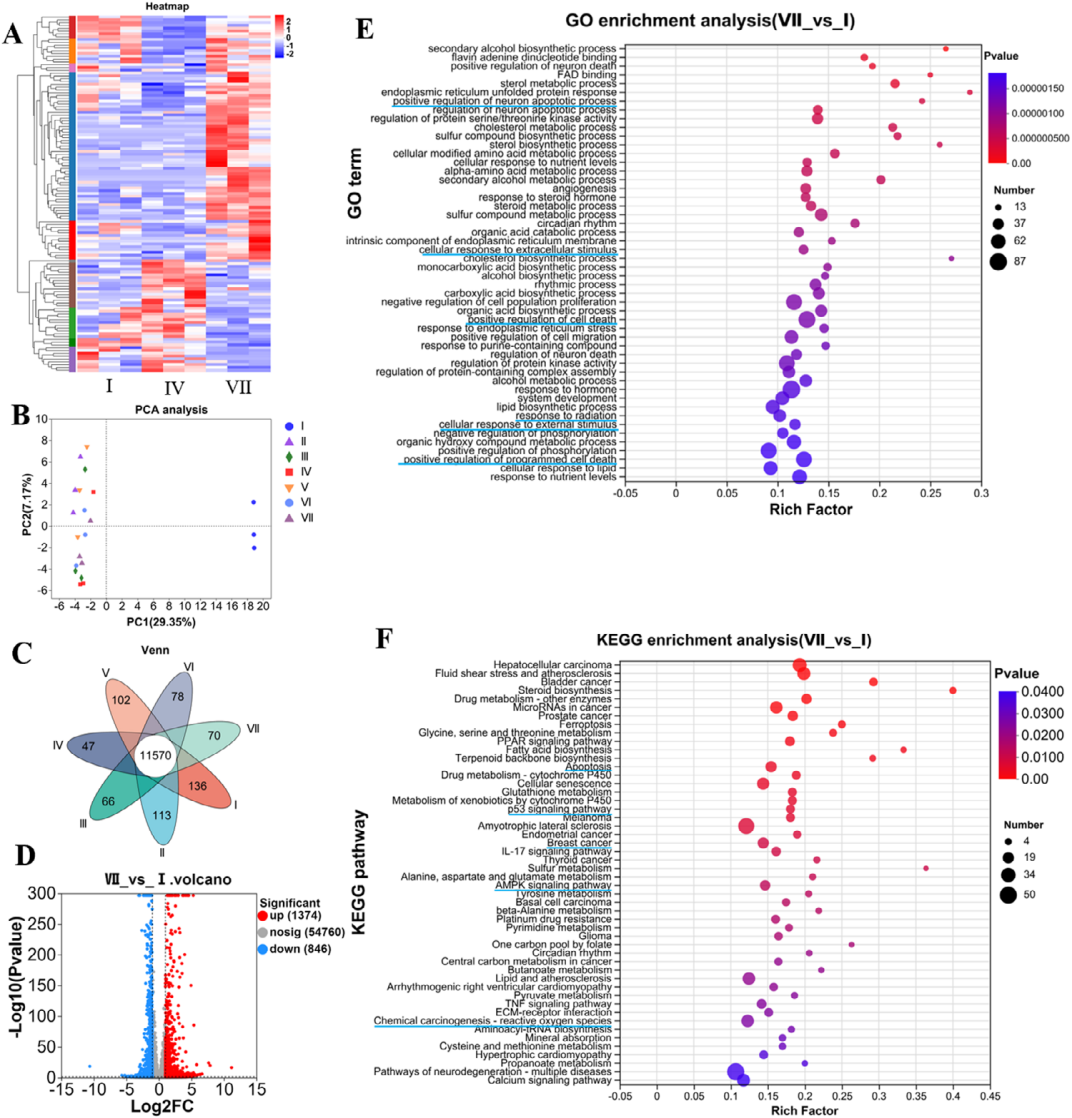
High‐throughput transcriptomic analysis. A) Heatmap diagram of differential gene expression in 4T1 cells after treatments with MGNSs@Pt with or without laser irradiation and control cells. B) Principal component analysis of differentially expressed genes in 4T1 cells after treatments with MGNSs, Pt NCs, MGNSs@Pt with or without laser irradiation. C) Calculating the intersection of the number of expressed genes with a Venn diagram in cells after treatments with MGNSs, Pt NCs, MGNSs@Pt with or without laser irradiation. D) Volcano plot showing upregulated (red) and downregulated (blue) genes in MGNSs@Pt + L group compared to control group. E) GO enrichment analysis and F) KEGG enrichment analysis of differential gene expression in MGNSs@Pt + L treated 4T1 cells compared to the control group. I: control, II: MGNSs, III: Pt NCs, IV: MGNSs@Pt, V: MGNSs + L, VI: Pt NCs + L, VII: MGNSs@Pt + L. Nanomaterial concentration: 100 µg mL^−1^, irradiation condition: 2 W cm^−2^, 5 min.

To further investigate the effect of MGNSs@Pt on 4T1 cells, high‐throughput transcriptomics analysis was carried out.^[^ ^32^ ^]^ The heat map showed hierarchical clustering of significantly differentially expressed genes (DEGs) in the 4T1 cells treated with MGNSs@Pt with or without 1064 nm laser irradiation and control cells (**Figure** **7**A). Principal component analysis (PCA) also highlighted two clearly distinct clusters among these cells with different treatments (Figure 7B; Figure S25A, Supporting Information). Among the 11570 genes detected using Venn analysis (Figure 7C), 136, 113, 66, 47, 102, 78, and 70 gene transcripts were in the control, MGNSs, Pt NCs, MGNSs@Pt, MGNSs + L, Pt NCs + L and MGNSs@Pt + L group, respectively. The genes expressed explicitly in MGNSs@Pt + L group were mainly attributed to the synergistic effect of PTT and catalytic therapy. Volcano plot and Minus‐Addition (MA) plot showed that a total of 2220 differentially expressed genes were identified in MGNSs@Pt + L group. Among them, 1374 genes were up‐regulated whereas 846 genes were down‐regulated (Figure 7D; Figure S25B, Supporting Information).

Furthermore, gene ontology (GO) enrichment analysis was applied to identify these genes biological functions process (Figure 7E; Figure S26A, Supporting Information). The varied expressed mRNAs were involved in regulation of programmed cell death and response to external stimulus in MGNSs@Pt+ L group, indicating MGNSs@Pt mediated thermal effect could contribute to the cell death. To better understand biological pathways and molecular networks of differentially expressed genes induced by MGNSs@Pt + L group, a bubble diagram of differential genes enriched in Kyoto Encyclopedia of Genes and Genomes (KEGG) pathway was analyzed (Figure 7F). MGNSs@Pt + L group involved in activating multiple pathways, including chemical carcinogenesis‐reactive oxygen pathway, AMPK signaling pathway, P53 signaling pathway, and cell apoptosis pathway, which further convinced that MGNSs@Pt + L group had better therapeutic effects by generating the oxidative stress due to the ROS produced by MGNSs@Pt and regulating cellular energy, cell cycle, DNA repair, and apoptosis by hyperthermia‐enhanced catalytic activities. Meanwhile, Gene Set Enrichment Analysis (GSEA) showcased a significant enrichment of genes associated with reactive oxygen pathway in MGNSs@Pt + L group (Figure S26B, Supporting Information), displaying the relevance of genes such as glutathione S‐transferase with the generation of reactive oxygen species. These results indicate that MGNSs@Pt plus laser irradiation could effectively eliminate tumor cells by multimodal synergistic therapy.

### 2.7 Biosafety and In Vivo Antitumor Effect of MGNSs@Pt

**Figure 8:**
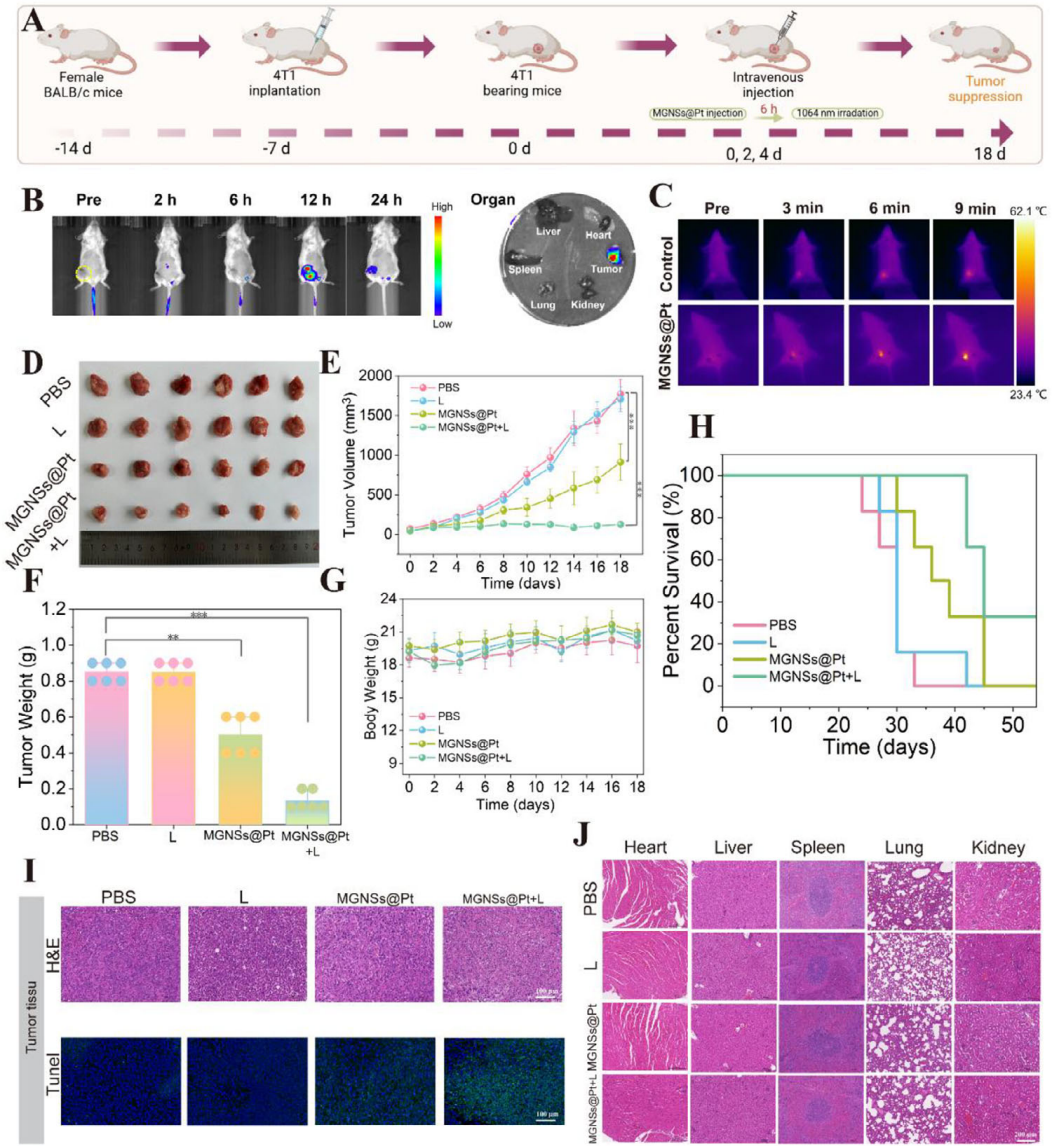
A) schematic illustration of 4T1 tumor xenograft establishment and in vivo experimental procedure and time schedule for evaluating the antitumor performance. B) Real‐time biodistribution images (left) of Cy5‐labeled MGNSs@Pt (10 mg kg^−1^) at different time points after tail vein injection and fluorescence imaging of main organs (heart, liver, spleen, lung, and kidney) and tumor (right). C) Photothermal images of 4T1 tumor‐bearing mice injected with and without MGNSs@Pt (10 mg kg^−1^) upon a 1064 nm laser (2 W cm^−2^). D) The dissected tumors photographs of 4T1‐tumor‐bearing mice after 18 days of different treatments. E) Tumor volume and F) tumor weight growth curves of 4T1‐tumor‐bearing mice after different treatments including control, Laser, MGNSs@Pt and MGNSs@Pt + L. Nanomaterial concentration: 10 mg kg^−1^, irradiation condition: 2 W cm^−2^, 5 min. G) Body weight monitoring for the indicated mice groups every two days. H) Kaplan‐Meier curves for mouse survival in the indicated groups for 50 days. I) H&E staining and TUNEL (green) images of tumor tissues harvested from mice after different treatments. Blue indicates DAPI staining of nuclei. Scale bar, 100 µm. J) H&E staining of major organs (heart, liver, spleen, lung, and kidney) of tumor tissues after 18 days of various treatments. Scale bar, 200 µm. Data are defined as mean ± SD (*n* = 4–6). Statistical significance is assessed by unpaired Student's two‐sided *t*‐test and asterisks indicate significant differences (*p < 0.05, **p < 0.01, and ***p < 0.001).

Based on the outstanding inhibition of cancer cell growth in vitro, we first performed a preliminary experiment using a group of tumor‐bearing mice, in which the three nanomaterials (MGNSs, Pt NCs and MGNSs@Pt) were intratumorally injected for comparative evaluation. The results showed that MGNSs@Pt group significantly inhibited tumor growth, while MGNSs showed little effect when compared to the control group (Figure S27, Supporting Information). Accordingly, we further focused in vivo antitumor activity of MGNSs@Pt by intravenously injecting them into mice bearing 4T1 xenograft tumors (**Figure** **8**A). The mice were randomly divided into four groups (control, laser, MGNSs@Pt and MGNSs@Pt + L, n = 6 per group). Firstly, Cy5‐labeled MGNSs@Pt was used to track nanomaterials biodistribution.^[^ ^33^ ^]^ As shown in Figure 8B and Figure S28A (Supporting Information), the fluorescence signal reached a maximum at 12 h within tumor region and a strong fluorescence signal was observed in the tumor compared with major organs (heart, liver, spleen, lung and kidney). Besides, the organs and tumor tissues were collected for ICP‐OES analysis. As shown in Figure S29 (Supporting Information), MGNSs@Pt efficiently accumulated into the tumor tissue with the relative distribution amount of 15.7% DI g^−1^ based on the typical enhanced permeability and retention effect (EPR) effect. Moreover, in situ photothermal imaging showed a temperature rise from 36.3 to 56.2 °C of the tumor site within 9 min after tail intravenous injection with MGNSs@Pt upon 5 min irradiation of 1064 nm laser. However, a minimal temperature fluctuated from 36.3 °C to 44.0 °C within 9 minutes in the control group (Figure 8C). These results suggested that MGNSs@Pt with 1064 nm laser irradiation had a high photothermal conversion efficiency, which undoubtedly met the required temperature for tumor hyperthermia.

Furthermore, tumor growth was monitored by measuring the sizes of the tumors every two days. Tumor growth suppression was observed in mice after the intravenous injection of MGNSs@Pt (Figure 8D). Notably, MGNSs@Pt + L group displayed remarkable antitumor effect and the tumor inhibition rate was as high as 95.5%; While only 49.0%, 2.3%, 2.1% of tumor inhibition rate were obtained in MGNSs@Pt, laser and PBS groups (Figure 8E). A similar result was also shown in the tumor weight curve, in which MGNSs@Pt + L group exhibited maximum tumor growth inhibition (Figure 8F). The satisfactory tumor inhibition efficiency was attributed to the catalytic activity and PTT therapy of MGNSs@Pt. Moreover, the body weight of mice showed no significant differences among four groups, indicating there was no serious toxicity effect toward mice during the therapeutic period (Figure 8G). Besides, the survival rate was improved in MGNSs@Pt + L group (Figure 8H). Further, Hematoxylin and eosin (H&E) staining was used to evaluate the pathological changes of the dissected tumor tissues in each group, in which MGNSs@Pt + L group exhibited larger cell necrotic areas (Figure 8I). The terminal deoxynucleotidyl transferase‐mediated dUTP nick‐end labeling (TUNEL) staining also showed that the MGNSs@Pt + L group had the obvious green fluorescence in tumor issues, further confirming maximum cell necrosis and apoptosis all the groups (Figure 8I; Figure S28B, Supporting Information). All treatment groups mice were harvested for histological analysis of the major organs (including the heart, liver, spleen, lungs and kidneys). No obvious pathological changes or noticeable damages were observed in all the groups (Figure 8J). Moreover, the complete blood count (CBC) and the acute liver and kidney function markers of the mice were evaluated after injection of MGNSs@Pt (Figures S30 and S31, Supporting Information). The levels of CBC indicators (including white blood cells (WBC), red blood cells (RBC), hemoglobin (HGB), hematocrit (HCT), mean corpuscular hemoglobin (MCH), mean corpuscular hemoglobin concentration (MCHC), platelets (PLT), mean corpuscular volume (MCV), platelet crit (PCT)), and serum biochemical markers [including alanine aminotransferase (ALT), aspartate aminotransferase (AST), urea or blood urea nitrogen (UREA) and creatinine (CREA)] remained within the reference ranges. And no significant differences were observed between wild type control mice and the MGNSs@Pt group. These results verified that the MGNSs@Pt exhibited outstanding performance in both tumor growth suppression efficacy, biocompatibility and safety, paving the way for their potential applications in various therapeutic area.

## 3 Conclusion

In summary, we have successfully synthesized a hydroxyl radical and oxygen nanogenerator of MGNSs@Pt with high photothermal conversion efficiency. The kinetic investigation demonstrated that MGNSs@Pt had a superior catalytic efficiency via using Michaelis−Menten theory. Compared to the Pt NCs and GNRs@Pt, MGNSs@Pt was successfully endowed with the enhanced catalytic activity due to the confinement effect of the mesopores in MGNSs. The DFT calculations and FEM simulation revealed that the MGNSs@Pt provided the adsorption ability for active species and the confinement‐induced enhancement of Pt NCs inside the mesopores. Moreover, such enhanced catalytic activity synergized with photothermal properties of MGNSs@Pt exerted an excellent antitumor therapeutic effect in the orthotopic 4T1 mouse models. This study provided a promising and safe strategy for designing NIR‐II light excitable nanozymes for wide biomedical applications in sensitive biosensing and effective antitumor therapy in the future.

## 4 Experimental Section

The detailed experimental methods were listed in the Supporting Information. Unless otherwise mentioned, all in vitro data represent means ± SD of three independent biological replicates. Statistical analysis was performed using an unpaired two‐tailed Student's *t*‐test with GraphPad Prism 9.5.0 (GraphPad Software, Inc., CA, USA). Animals were cared for and maintained under the Guidelines of Laboratory Animals of Fudan University and approved by the Animal Ethics Committee of Fudan University, China (approved No. FE221871).

## Conflict of Interest

The authors declare no conflict of interest.

## Author Contributions

H.C. and F.C. conceived and designed the project. F.C., J.C., H.L., Y.K., and M.Y. performed the experiments. J.K. and X.L. supervised the project. F.C. and M.Y. analyzed data. H.C., M.W., X.L., and F.C. wrote and edited the manuscript. All authors discussed the results and contributed to the preparation.

## Supporting information



Supporting Information

Supplemental Data 1

## Data Availability

The data that support the findings of this study are available in the supplementary material of this article.
