# Supplementary material for: Mesoporous Gold Nanospheres Confined Platinum Nanoclusters as Robust ROS and Oxygen Nanogenerators for NIR‐II Hyperthermia Cancer Therapy
Source: Adv Sci (Weinh). 2025 May 11;12(28):2502688. doi: 10.1002/advs.202502688 (PMC12302635; doi:10.1002/advs.202502688)
Supplement: Supplementary file 1 — Supporting Information [file ADVS-12-2502688-s002.docx]

**Supporting Information**

**Mesoporous Gold Nanospheres Confined Platinum Nanoclusters as Robust ROS and Oxygen Nanogenerators for NIR-II Hyperthermia Cancer Therapy**

Fei Cun^a^, Jie Chen^a^, Hanxue Li^a^, Yufang Kou^a^, Meiyan Wang^b,^ *, Xiaomin Li^a,^ *, Hui Chen^a^ *, Jilie Kong^a,^ *

^a^ Department of Chemistry, Fudan University, Shanghai, 200438, China

^b^ 411 Hospital, School of Medicine, Shanghai University, Shanghai 200444, China

*Corresponding authors

E-mail: chenhui@fudan.edu.cn, wangmy@shu.edu.cn, [lixm@fudan.edu.cn,](mailto:lixm@fudan.edu.cn,) jlkong@fudan.edu.cn

**Table of contents**

[1. Experimental Section 4](#_Toc4501)

[Materials 4](#_Toc1781)

[Characterization 4](#_Toc514)

[Preparation of Mesoporous Gold Nanospheres 5](#_Toc32195)

[Preparation of MGNSs@Pt 6](#_Toc2164)

[Preparation of GNRs@Pt and Pt NCs 7](#_Toc24695)

[Evaluation of Peroxidase (POD)-like Activity and Kinetic Assay 8](#_Toc23135)

[Comparison of Peroxidase-like Activity with Three Different Catalysts 9](#_Toc26690)

[Evaluation of Catalase (CAT)-like Activity 9](#_Toc1911)

[Evaluation of Photothermal Performance 10](#_Toc23597)

[Density Functional Theory (DFT) Calculations 10](#_Toc24635)

[Theoretical calculation based on finite element method (FEM) 12](#_Toc16875)

[Cell Culture and Viability Assay 13](#_Toc13826)

[Intracellular ROS Evaluation 13](#_Toc9787)

[Intracellular O_2_ Evaluation 14](#_Toc26687)

[Dead/Live Staining. 14](#_Toc16792)

[LDH Assay 15](#_Toc4373)

[JC-1 Assay 15](#_Toc8949)

[Annexin V-FITC/PI Apoptosis Assay. 16](#_Toc29564)

[Western Blot 16](#_Toc6624)

[RNA Sequencing 17](#_Toc8060)

[Tumor Mouse Model 19](#_Toc8772)

[In Vivo Biodistribution Imaging 19](#_Toc9543)

[In Vivo Photothermal Imaging 19](#_Toc26521)

[Evaluation of Anti-tumor Efficacy in Vivo 20](#_Toc13893)

[Statistical Analysis 22](#_Toc25163)

[2. Supporting Figures 23](#_Toc19271)

[Figure S1. 23](#_Toc27035)

[Figure S2. 24](#_Toc24951)

[Figure S3. 25](#_Toc9546)

[Figure S4. 26](#_Toc11212)

[Figure S5. 27](#_Toc26055)

[Figure S6. 28](#_Toc17665)

[Figure S7. 29](#_Toc24258)

[Figure S8. 30](#_Toc3682)

[Figure S9. 31](#_Toc24392)

[Figure S10. 32](#_Toc2545)

[Figure S11. 33](#_Toc15322)

[Figure S12. 34](#_Toc12967)

[Figure S13. 35](#_Toc537)

[Figure S14. 36](#_Toc11238)

[Figure S15. 37](#_Toc2817)

[Figure S16. 38](#_Toc10585)

[Figure S17. 39](#_Toc26567)

[Figure S18. 40](#_Toc15048)

[Figure S19. 41](#_Toc18133)

[Figure S20. 42](#_Toc17331)

[Figure S21. 43](#_Toc6521)

[Figure S22. 44](#_Toc25782)

[Figure S23. 45](#_Toc18499)

[Figure S24. 46](#_Toc3322)

[Figure S25. 47](#_Toc29485)

[Figure S26. 48](#_Toc23616)

[Figure S27 49](#_Toc25584)

[Figure S28. 50](#_Toc595)

[Figure S29. 51](#_Toc19524)

[Figure S30. 52](#_Toc28375)

[Figure S31. 53](#_Toc30424)

[3. Supporting Tables 54](#_Toc31959)

[Table S1. Detailed parameters for synthesis of MGNSs@Pt and the size of Pt NCs in each catalyst. 54](#_Toc2369)

[Table S2. A summary table with the XPS results of MGNSs@Pt 55](#_Toc24449)

[Table S3. Au and Pt contents for three catalysts, the particle size of Pt NCs and total size in each catalyst. 56](#_Toc9782)

[Table S4. Photothermal conversion efficiency of the Pt or Au-related metallic catalyst 57](#_Toc489)

[Table S5. Comparison of the catalytic efficiencies (K_cat_) of Pt or Au-related metallic catalyst 58](#_Toc31438)

[Table S6. The model parameters used in COMSOL Multiphysics 59](#_Toc22994)

[References 60](#_Toc22179)

# Experimental Section

**Materials**

1,2-dipalmitoyl-sn-glycero-3-phosphocholine (DPPC) was purchased from Xi’an ruixi Biological Technology Co., Ltd. (Xi ’an, China). Cholesterol, ascorbic acid (AA), poly(vinylpyrrolidone) (PVP MW 10 kDa), bovine serum albumin (BSA), HAuCl_4_·3H_2_O and H_2_PtCl_6_·6H_2_O were from Sigma-Aldrich (St Louis, MO, USA). H_2_O_2_, acetic acid and sodium acetate were from Shanghai Chemical Reagent Co., Ltd. (Shanghai, China). TMB solution (1%) was purchased from Solarbio Co., Ltd. (Beijing, China). Acetoxymethylester of calcein (Calcein-AM), propidium iodide (PI), 2′,7′-dichlorodihydrofluorescein diacetate (DCFH-DA) and JC-1 staining kit were purchased from Beyotime Co., Ltd. (Shanghai, China). Cell counting kit-8 (CCK-8) was purchased from Ruilong Biomedical Technology Co., Ltd. (Shanghai, China). [Ru(dpp)_3_]^2+^Cl_2_ was purchased from Maokang Biotechnology Co., Ltd. (Shanghai, China). LDH assay kit was purchased from Thermo Fisher Scientific (USA). Glutathione peroxidase 4 (GPx4) rabbit mAb, Bcl2 mouse mAb, Bax rabbit mAb were purchased from ZEN-BIOSCIENCE (Chengdu, China), β-actin was purchased from Yeasen Biotechnology Co., Ltd. (Shanghai, China). Anti-Caspase-3 antibody and anti-HIF-1α antibody were purchased from Abcam (Cambridge, UK).

## Characterization

Field emission scanning electron microscopy (FESEM) images were obtained on Zeiss Ultra 55 (Germany) at 3 kV and 10 μA. The transmission electron microscope (TEM) and high-resolution transmission electron microscope (HRTEM) were conducted on a HT7700 transmission electron microscope of Hitachi with an acceleration voltage of 120 kV. The high-angle annular dark-field scanning transmission electron microscopy (HAADF-STEM) and Elemental mapping analysis were conducted on a Tecnai G2 F20S-Twin transmission electron microscope of FEI with an acceleration voltage of 200 kV. The UV-vis absorption spectra were recorded using a Lambda365 spectrometer (PerkinElmer) and a DNM-9602 microplate reader (Beijing Perlong New Technology Co. Ltd., Beijing, China) in 96-well plates. The particle concentration of MGNSs@Pt was analyzed by a commercial Nanoparticle Tracking Analysis (NTA) system ZetaView PMX 110 (Particle Metrix, Meerbusch, Germany). The inductively coupled plasma optical emission spectroscopy (ICP-OES) were conducted on an iCAP7400 spectrometer (Thermo Fisher Scientific). Bruker D8 powder X-ray diffractometer (XRD) using Cu Ka radiation (40 kV, 40 mA) was used for the collection of XRD patterns. X-ray photoelectron spectroscopy (XPS) data were obtained by a K-Alpha electron spectrometer (Thermo Fisher Scientific).

**Preparation of** **Mesoporous Gold Nanospheres**

According to the Wang at al.,^[1]^ MGNSs were synthesized in a soft-templating approaches by using liposomes. For liposomes preparation, 50.0 mg of DPPC and 21.5 mg of cholesterol (molar ratio was 55:45) were dissolved in 20 ml of chloroform and the organic solvent was completely removed using a rotary evaporator. Then, AA solution (0.3 M) was added to the lipid residual until the concentration of the solute was 1 mg mL^-1^. After ultrasonication for 20 min, the liposomes solution was centrifugated at 10000 rpm for 20 min which was ready for next synthesis step.

For preparing MGNSs, 400 μL of HAuCl_4_·3H_2_O solution (8 mM) was dropped into the supernatant of liposome solution (1 mL) via a stable and constant rate (100 μL min^-1^) controlled by a micro-injection pump. After stirring for 1 h, the products were collected by centrifugation and washed three times with deionized water to remove the residual reactants and liposomes. It should be noted that a syringe pump was necessary to precisely control the addition and amount of reagents for uniform morphology and size of the synthesized MGNSs.

**Preparation of MGNSs@Pt**

To deposit platinum nanoclusters onto mesoporous gold, poly(vinylpyrrolidone) (PVP) was used as a stabilizer and adsorbent reagent for Pt^4+^.^[2]^ Briefly, 250 μL of MGNSs (157 μg) was diluted by deionized water to 1000 μL and ultrasonicated for 10 minutes before use, which will effectively preventing aggregation and precipitation. Then, 20 μL of PVP (20 wt %) was added to the MGNSs solution and ultrasonicated for 30 min. After adding 80 μL of AA solution (100 mg mL^-1^) and H_2_PtCl_6_·6H_2_O (40 μL of solution with different concentrations), the mixture was incubated at 65 ℃ and kept mild stirring for 1 h. Finally, MGNSs@Pt were collected by centrifugation and purified by washing for three times, and redispersed in deionized water.

## Preparation of GNRs@Pt and Pt NCs

The gold nanorods were synthesized followed by Ye at al.^[3]^ Briefly, 5 mL of 0.5 mM HAuCl_4_ was mixed with 5 mL of 0.2 M CTAB solution. 0.6 mL of fresh 0.01 M NaBH_4_ was diluted to 1 mL with water and then injected to the Au (III)-CTAB solution. The solution color changed from yellow to brownish yellow and the stirring was stopped after 2 min. The seed solution was aged at room temperature for 30 min before use. Then, 7.0 g of CTAB and a certain quantity of sodium oleate (NaOL) were dissolved in 250 mL of warm water (~50 ℃). The solution was allowed to cool down to 30 ℃ and 4 mM AgNO_3_ solution was added. The mixture was kept undisturbed at 30 ℃ for 15 min after adding 250 mL of 1 mM HAuCl_4_ solution. The solution became colorless after 90 min of stirring and a certain volume of HCl (37 wt. % in water, 12.1 M) was then introduced to adjust the pH. After another 15 min of slow stirring at 400 rpm, 1.25 mL of 0.064 M AA was added and the solution was vigorously stirred for 30 s. Finally, a small amount of seed solution was injected into the growth solution. The resultant mixture was stirred for 30 s and left undisturbed at 30°C for 12 h for NR growth. The final products were centrifugated at 7,000 rpm for 30 min.

For the preparation of Pt nanoclusters,^[4]^ 0.5 mL of BSA solution (20 mg mL^-1^) was mixed with H_2_PtCl_6_·6H_2_O (20 mM 0.5 mL). Then, 0.05 mL of NaOH (1 M) was added. The solution was incubated at 80 ℃ and kept stirring for 2 h. The final product was washed at least 5 times by ultrafiltration (Millipore, 10 kDa).

## Evaluation of Peroxidase (POD)-like Activity and Kinetic Assay

Peroxidase-like activity of MGNSs@Pt was evaluated using Michaelis-Menten, with nanozyme activity quantified by analyzing the reaction rates at varying substrate concentrations, and the data were further analyzed using the double reciprocal (Lineweaver-Burk) plot.^[5]^ The experiments were conducted as follows: 180 μL of TMB solution at various concentrations (0.05 mM, 0.1 mM, 0.2 mM, 0.3 mM, 0.4 mM, 0.5 mM and 0.6 mM) was mixed with 180 μL of 0.2 M H_2_O_2_ (pH=6.5). Alternatively, 180 μL of H_2_O_2_ solution at various concentrations (0.025 mM, 0.05 mM, 0.1 mM, 0.2 mM, 0.3 mM, 0.4 mM and 0.5 mM) was mixed with 180 μL of 1.1 mM TMB solution. Then, 20 μL of MGNSs@Pt (0.047 μg) were added to initiate the reaction and the time scan mode of the UV-vis spectrophotometer was used with an interval of 2 sec for 1 min. Then, the reaction velocity ($v$) was obtained from the “Absorbance *versus* Time” curve, which could be fitted by the Michaelis-Menten equation:

$$\frac{1}{v}=\frac{K_{M}}{v_{max}\cdot[S]}+\frac{1}{v_{max}}$$

Where$v_{max}$ was the maximal reaction velocity,

$[S]$ was the substrate concentration of TMB or H_2_O_2_

$K_{M}$ was the Michaelis constant

The kinetic parameters, $K_{M}$ and $v_{max}$ could be calculated from the double reciprocal equation. The catalytic efficiency $K_{cat}$ was obtained from the equation:

$$K_{cat}=\frac{v_{max}}{[E]}$$

Where $[E]$ was the particle concentration of catalysts.

## Comparison of Peroxidase-like Activity with Three Different Catalysts

The peroxide-like activities of MGNSs@Pt, GNRs@Pt and Pt NCs were compared. For GNRs@Pt, the mass of gold and platinum elements was determined by ICP (Table S2) and should be roughly kept the same to that of MGNSs@Pt in each assay. And the mass of platinum in Pt NCs should be kept consistent with that of MGNSs@Pt.

## Evaluation of Catalase (CAT)-like Activity

The CAT-like activity of MGNSs@Pt was evaluated by the generation of oxygen dissolved in the H_2_O_2_ solution using a portable oxygen meter (Leici JPBJ-608) at room temperature. Briefly, 10 mM H_2_O_2_ solution (pH=6.5) was mixed with MGNSs@Pt solution at various concentrations (25, 50 and 75 μg mL^-1^). The generation of O_2_ was recorded every 30 s at 37 °C with a dissolved oxygen meter.

## Evaluation of Photothermal Performance

The temperature was acquired by placing the solution to be measured in a 2 mL glass vial, with the 1064 nm laser probe positioned 1 cm away from the vial. MGNSs@Pt aqueous solution with various concentration (25, 50, 75, 100 μg mL^-1^) was exposed to a 1064 nm laser for 300 s at room temperature with a power intensity of 2 W cm^-2^. The temperature and thermal images were monitored and recorded using a non-contact infrared (IR) Thermometers (Fluke Ti480 PRO Infrared Camera). 100 μg mL^-1^ of MGNSs@Pt were irradiated at 2 W cm^-2^ for several cycles, where each cycle consisted of a heating period followed by a natural cooling period to assess their photothermal stability.

## Density Functional Theory (DFT) Calculations

All the theoretical calculations were carried out with density functional theory (DFT) method as implemented in the Vienna Ab Initio Simulation Package (VASP).^[6]^ The electron ion interaction was described with the projector augmented wave (PAW) method, while the electron exchange and correlation energy were solved within the generalized gradient approximation with the revised Perdew-Burke-Ernzerhof (RPBE) exchange-correlation functional.^[7]^ The empirical correction in Grimme’s method (DFT+D3) was used to describe van der Waals interaction, and the dipole correction was employed to correct potential spurious terms arising from the asymmetry of the slabs.^[8]^ The kinetic energy cutoff of plane wave was set to be 400 eV and the convergence criterion for the residual forces and total energies were set to be 0.03 eV Å^−1^ and 10^−5^ eV, respectively.

A *p*(4 × 4) four-atomic-layer slab was used to model the Au(111) and Pt(111) surface, while Pt cluster was constructed with 10 Pt atoms supported on the Au(111) surface with *p*(5 × 5) four atomic-layers. For these three catalysts, the bottom two layers were fixed during the structural relaxation. A vacuum layer of 25 Å was set between the periodically repeated slabs to avoid strong interactions and a 3×3×1 Monkhorst-Pack k-point grids was used to sample the Brillouin zone.

The Gibbs free energy change (ΔG) of each elementary step was calculated by using the computational hydrogen electrode (CHE) model reported by Nørskov et al.^[9]^ In this model, the chemical potential of the proton-electron pair in aqueous solution is related to that of one-half of the chemical potential of an isolated hydrogen molecule. The ΔG value can be obtained by the formula: ΔG = ΔE + ΔZPE − TΔS, where ΔE is the reaction energy of reactant and product species adsorbed on the catalyst directly obtained from DFT calculations; ΔZPE and ΔS are the changes between the adsorbed species and the gas phase molecules in zero-point energies and entropy at 298.15 K, which can be calculated from the vibrational frequencies.

## Theoretical calculation based on finite element method (FEM)

COMSOL Multiphysics software was used to build the nanoconfinement effect of MGNSs@Pt and GNRs@Pt was used for comparison. To analyze the difference in the catalytic efficiency of two types of nanoparticles to produce ·OH in the experiment, the mass transfer equation in COMSOL was used to calculate ·OH production process of the two single particles:

$$\frac{\partial c_{i}}{\partial t}+\nabla\cdot\mathbf{J}_{i}=R_{i}$$

where $c_{i}$ was the concentration, $R_{i}$ was the reaction rate, and $\mathbf{J}_{i}$ was the flux that depended on concentration gradient and diffusion coefficient $D_{i}$:

$$\mathbf{J}_{i}=-D_{i}\nabla c_{i}$$

Appling surface reaction boundary conditions to the particle surface:

$$-\mathbf{n}\cdot\mathbf{J}_{i}=J_{0,i}$$

where $\mathbf{n}$ is the surface normal vectors, and $J_{0,i}$ (unit: mol/(m^2^·s)) was the surface reaction rate which can be calculated by

$$J_{0,i}= \frac{v_{i}}{N_{V}s}$$

where $v_{i}$ (unit: mol/(m^3^·s)) was the total reaction rate，$N_{V}$ (unit: 1/m^3^) was the nanoparticle concentration，$s$ (unit: m^2^) was nanoparticle surface area. The total reaction rate had the following relationship:

$$\frac{1}{v_{H_{2}O_{2}}}= \frac{K}{v_{max}[S]}+\frac{1}{v_{max}}$$

$$v_{HO\cdot}=2v_{H_{2}O_{2}}$$

The corresponding parameters were listed in Table S5.

## Cell Culture and Viability Assay

HUVEC or 4T1 cells were cultured in Dulbecco’s modified eagle medium (DMEM) containing 10% (v/v) fetal bovine serum (FBS) and 1% (v/v) penicillin/streptomycin, and incubated at 37°C in a humidified atmosphere containing 5% CO_2_. For cytotoxicity assay, 4T1 or HUVEC cells were seeded into 96-well plates at a density of 5000 cells per well and cultured overnight. Then the culture media were replaced by fresh medium containing three kinds of nanomaterials with different concentrations (0, 12.5, 25, 50, 100, 200 µg mL^−1^). 4 hours later, cells were irradiated with or without a 1064 nm laser (2 W cm^-2^, 5 min per well). After another 24 hours of cultivation, the cell counting kit-8 (CCK-8) assay was conducted to assess the cell activity. CCK-8 solution was mixed with FBS-free culture medium at a volume ratio of 1:10. Then, 100 µL of the mixture was added to each well and incubated at 37℃ in a 5% CO_2_ incubator for 1 hour. The absorbance at 450 nm was detected using the microplate reader.

## Intracellular ROS Evaluation

DCFH-DA was used to evaluate the ability of cells to generate ROS. First, the 4T1 cells were cultured in the 6-well plate 24 h before treating with nanomaterials (100 µg mL^−1^) for 4 hours. Cells were washed twice with PBS three times and irradiated with or without a 1064 nm laser (a power intensity of 2 W cm^-2^ for 5 min). After another 4 h, the culture media were replaced by 1 mL DCFH-DA (10 μM in FBS-free DMEM) and incubated for 20 min, allowing fully contact between the probe and cells. The cells were washed with PBS three times and the level of intracellular ROS was evaluated by detecting the fluorescence of DCF (λ_ex_ = 488 nm, λ_em_ = 517 nm) using a confocal laser scanning microscope (CLSM).

## Intracellular O_2_ Evaluation

[Ru(dpp)_3_]^2+^Cl_2_ was applied to be a O_2_ probe, which can be visualized with red fluorescence. Briefly, the 4T1 cells were cultured in the 6-well plate under hypoxic conditions (5 % CO_2_, 1 % O_2_ and 94 % N_2_). After 24 h, the cells were incubated with three kinds of nanomaterials including MGNSs, Pt NCs, MGNSs@Pt (100 µg mL^−1^) for 4 h and irradiated with or without a 1064 nm laser (a power intensity of 2 W cm^-2^ for 5 min). After another 4 h of incubation, cells were washed twice with PBS three times and stained by [Ru(dpp)_3_]^2+^Cl_2_ with a final concentration of 10 μM for 20 min. Finally, the cells were washed and analyzed by using a CLSM.

## Dead/Live Staining.

Calcein/PI Cell Viability/Cytotoxicity Assay Kit was utilized to analyze the living and dead cells. 4T1 cells were first cultured overnight. Then the cells were incubated three kinds of nanomaterials including MGNSs, Pt NCs, MGNSs@Pt (200 µg mL^-1^) for 4 h and irradiated with or without a 1064 nm laser (a power intensity of 2 W cm^-2^ for 5 min). After the co-incubation for an additional 24 hours, cells were washes twice with PBS. Afterwards, calcein-AM and PI were used to stain 4T1 cells for 30-min. Finally, the cells were again washed twice with PBS and observed using fluorescence microscopy.

## LDH Assay

The 4T1 cells were seeded in the 6-well culture plate and allowed to adhere overnight. Then the cells were treated with three kinds of nanomaterials including MGNSs, Pt NCs, MGNSs@Pt (100 µg mL^−1^) for 4 h and irradiated with or without a 1064 nm laser (a power intensity of 2 W cm^-2^ for 5 min). After a 4-hour incubation period of the nanomaterials. The supernatants of the cells subjected to various treatments were harvested and centrifuged. The quantification of LDH leakage was analyzed using a cytotoxicity LDH assay kit, according to the manufacturer's instructions. Finally, the absorbance at 490 nm was measured using a microplate reader (BioTek Instruments).

## JC-1 Assay

In order to assess the intracellular mitochondrial membrane potential, the cells were treated with three kinds of nanomaterials including MGNSs, Pt NCs, MGNSs@Pt (100 µg mL^-1^) for 4 h and were irradiated with or without a 1064 nm laser (a power intensity of 2 W cm^-2^ for 5 min). Then cells were stained with a JC-1 assay kit for a 4 h. according to the manufacturer’s protocols. Subsequently, the cells were additionally stained with DAPI for 15 minutes to label the nucleus. Finally, the cells were analyzed using CLSM.

## Annexin V-FITC/PI Apoptosis Assay.

Cell apoptosis was assessed using the Annexin V-FITC/Propidium Iodide (PI) double-staining apoptosis detection kit (C1062M, Beyotime Biotechnology). The 4T1 cells (2×10^5^ cells/well) were cultured in the 6-well plate and allowed to adhere overnight. Then the cells were treated were treated with three kinds of nanomaterials including MGNSs, Pt NCs, MGNSs@Pt (100 µg µL^-1^) for 4 h, and irradiated with or without a 1064 nm laser (a power intensity of 2 W cm^-2^ for 5 min). The collected cells were washed 3 times with PBS, resuspended in 195 μL Annexin V-FITC binding solution and 5 μL Annexin V-FITC, followed by adding 10 μL propidium iodide staining solution and gentle mixing. The mixture was then incubated at room temperature in the dark for 15 min. Subsequently, cell apoptosis analysis was performed by LSRFortessaTM Flow Cytometer (BD Biosciences).

## Western Blot

The 4T1 cells were seeded in 12-well culture plates and subjected to different treatments. Following a 24-hour incubation period, cells were harvested and the proteins were extracted. Subsequently, Then, cell lysates were resolved on a 12% SDS polyacrylamide gel and electroblotted onto polyvinylidene fluoride (PVDF) membranes. Subsequently, the samples were blocked with 5% non-fat milk for 1 hour at room temperature. The membranes were then incubated overnight at 4 °C with primary antibodies:Caspase 3 (1:1000, Abcam), Bcl (1:500, ZEN-BIOSCIENCE), Bax (1:500, ZEN-BIOSCIENCE), GPx4 (1:500, ZEN-BIOSCIENCE), HIF-1α (1:1000, Abcam), and β-actin actin (1:10000, Yeasen) followed by secondary antibodies: Alexa Fluor 594 AffiniPure Goat Anti-Mouse IgG(H+L) (1:5000, YEASEN) at room temperature for 1 h. Membranes were visualized using a fluorescent western blot imaging system (LI-COR Odyssey Biosciences).

## RNA Sequencing

4T1 cells were treated with MGNSs, Pt NCs, MGNSs@Pt with or without 1064 nm laser irradiation. Total RNA from these cells (5 μg) was extracted using Trizol reagent for constructing RNA-seq transcriptome library. Shortly, messenger RNA was isolated according to the polyA selection method by oligo (dT) beads and then fragmented by the fragmentation buffer. Subsequently, double-stranded cDNA was synthesized using a SuperScript double-stranded cDNA synthesis kit (Invitrogen, CA, USA) with random hexamer primers (Illumina). Then the synthesized cDNA was subjected to end-repair, phosphorylation and ‘A’ base addition according to the Illumina’s library construction protocol. Libraries were selected for cDNA target fragments of 200–300 bp on 2% Low Range Ultra Agarose followed by PCR amplified using Phusion DNA polymerase (New England Biolabs (Beijing) LTD.; China) for 15 PCR cycles. After quantified by TBS380, paired-end RNA-seq sequencing library was sequenced with the Illumina HiSeq Xten (2 × 150 bp read length).

The raw paired end reads were trimmed and quality controlled by the SeqPrep (https://github.com/jstjohn/SeqPrep) and the Sickle (https://github.com/najoshi/sickle) with default parameters. Then clean reads were separately aligned to reference genome with orientation mode using the TopHat (http://tophat.cbcb.umd.edu/, version2.0.0) software [67]. The mapping criteria of bowtie was as follows: sequencing reads should be uniquely matched to the genome allowing up to 2 mismatches, without insertions or deletions. Then the region of gene was expanded following depths of sites and the operon was obtained. In addition, the whole genome was split into multiple 15 kb windows that share 5 kb. New transcribed regions were defined as more than 2 consecutive windows without overlapped region of gene, where at least 2 reads mapped per window in the same orientation. The data were analyzed on the online Majorbio Cloud platform.

## Tumor Mouse Model

The 6-8 weeks-old BALB/c female mice (20 ± 2 g) were purchased from Hangzhou Ziyuan Laboratory Animal Co., Ltd (Zhejiang, China). All animal experiments were strictly conducted according to the regulations of the Institutional Animal Care and Use Committee of China with the approval of the Animal Ethics Committee of Fudan University (approved No. FE221871). To establish the orthotopic 4T1 breast tumor model, 4T1 cells (1×10^7^ cell mL^-1^,100 μL) was injected into the upper right mammary fat pads.

## In Vivo Biodistribution Imaging

After the intravenous injection of Cy5-loaded MGNSs@Pt (10 mg kg^−1^) into 4T1 tumor-bearing mice, fluorescence signals at the tumor sites were monitored at various time points (0, 2, 6, 12, and 24 h) using the IVIS Lumina II in vivo imaging system (Perkin Elmer, USA). Then, the mice were euthanized, and the heart, liver, spleen, lung, kidney, and tumor tissues were collected for fluorescence imaging. Meanwhile, the collected organs and tumor tissues were processed using a tissue grinder, followed by digestion with 2 mL of chloroazotic acid. After 24 hours, the solutions were filtered to remove residual tissue debris, and the supernatants were used for ICP-OES analysis.

## In Vivo Photothermal Imaging

4T1 tumor-bearing mice were intravenously injected with MGNSs@Pt (10 mg kg^−1^). After 12 h, tumor sites were exposed to a 1064 nm laser (2 W cm^-2^) for 9 min. The thermal images were obtained by using a non-contact infrared Thermometers.

## Evaluation of Anti-tumor Efficacy in Vivo

For the evaluation of intratumoral injection, the 4T1 tumor-bearing mice were subsequently used after the tumor volume reached to 25-40mm^3^. The 4T1 tumor-bearing mice were randomly separated into four groups (n = 6), and injected with PBS (Control), MGNSs (10 mg kg^-1^), Pt NCs (10 mg kg^-1^), and MGNSs@Pt (10 mg kg^-1^) at day 0, 2, and 4. The weight and volume of tumors were measured every two days during the treatment.

For the evaluation of intravenous injection, the 4T1 tumor-bearing mice were subsequently used after the tumor volume reached to 50-100 mm^3^. The 4T1 tumor-bearing mice were randomly divided into four groups (n = 6) including control (PBS), 1064 nm laser (2 W cm^-2^, 5 min), MGNSs@Pt and MGNSs@Pt + laser (irradiated by 1064 nm laser for 5 min after 12 h injection of MGNSs@Pt). The four groups were intravenously injected with PBS or MGNSs@Pt (10 mg kg^−1^) at day 0, 2, and 4. The tumor volume was detected and recorded every two days by caliper measurement. Tumor volume was calculated by measuring the length and width of each tumor using calipers following formula: tumor volume = L × W^2^/2, where L and W mean the length (mm) and width (mm) of tumor, respectively. The protocol requires animals to be euthanized when the tumor volume burden reaches 2000 mm^3^.

The dissected tumor tissues and major organs (including heart, liver, spleen, lung, and kidney) were stained with a Hematoxylin and eosin (H&E) staining kit (C0105M, Beyotime Biotechnology) for histological analysis according to the manufacturer’s instructions. Briefly, the tissue sections were dehydrated by increasing alcohol concentrations and cleared using xylene (Sangon Biotech; CAS: 1330-20-7) and stained with the hematoxylin staining solution for 8 min and washed in running tap water for 10 min. Next, the tissue sections were differentiated in 1% acid alcohol for 10 s, washed in running tap water for 5 min, and then were counterstained in the eosin staining solution for 30 s to 1 min and washed in running tap water for 5 min. Finally, the tissue sections were sealed by a drop of mounting medium over the tissue and then covered by a coverslip. The slides were then observed by a BX53 upright microscope (Olympus, Japan) equipped with an Olympus digital camera

The terminal deoxynucleotidyltransferase mediated nick end labeling (TUNEL) staining kit (C1088, Beyotime Biotechnology) was used to analyze the apoptosis levels of tumor tissues the structure of tumors according to the manufacturer’s instructions. Briefly, frozen tumor tissue sections were fixed in a fixation solution (4% paraformaldehyde) for 30 min. Then the tissue sections were washed two times with PBS for 10 min each and treated with a permeabilization solution (0.5% Triton X-100 in PBS) for 5 min. Subsequently, the tissue sections were incubated with 50 μL TUNEL solution at 37°C in the dark for 60 min. Fluorescence images were obtained from a BX53 upright fluorescence microscope (Olympus, Japan). The nuclei of apoptotic cells were stained green, and all other nuclei were stained blue.

**Statistical Analysis**

Unless otherwise mentioned, all *in vitro* data represent means ± SD of three independent biological replicates. For the animal experiments, each treatment group consisted of randomly selected mice (n = 3-6) and the results are expressed as means ± SD. Statistical analysis was performed using an unpaired two-tailed Student’s *t*-test with GraphPad Prism 9.5.0 (GraphPad Software, Inc., CA, USA). Results were considered statistically significant at **p* < 0.05 (moderate significance), ***p* < 0.01 (high significance), and ****p* < 0.001 (very high significance).

# Supporting Figures


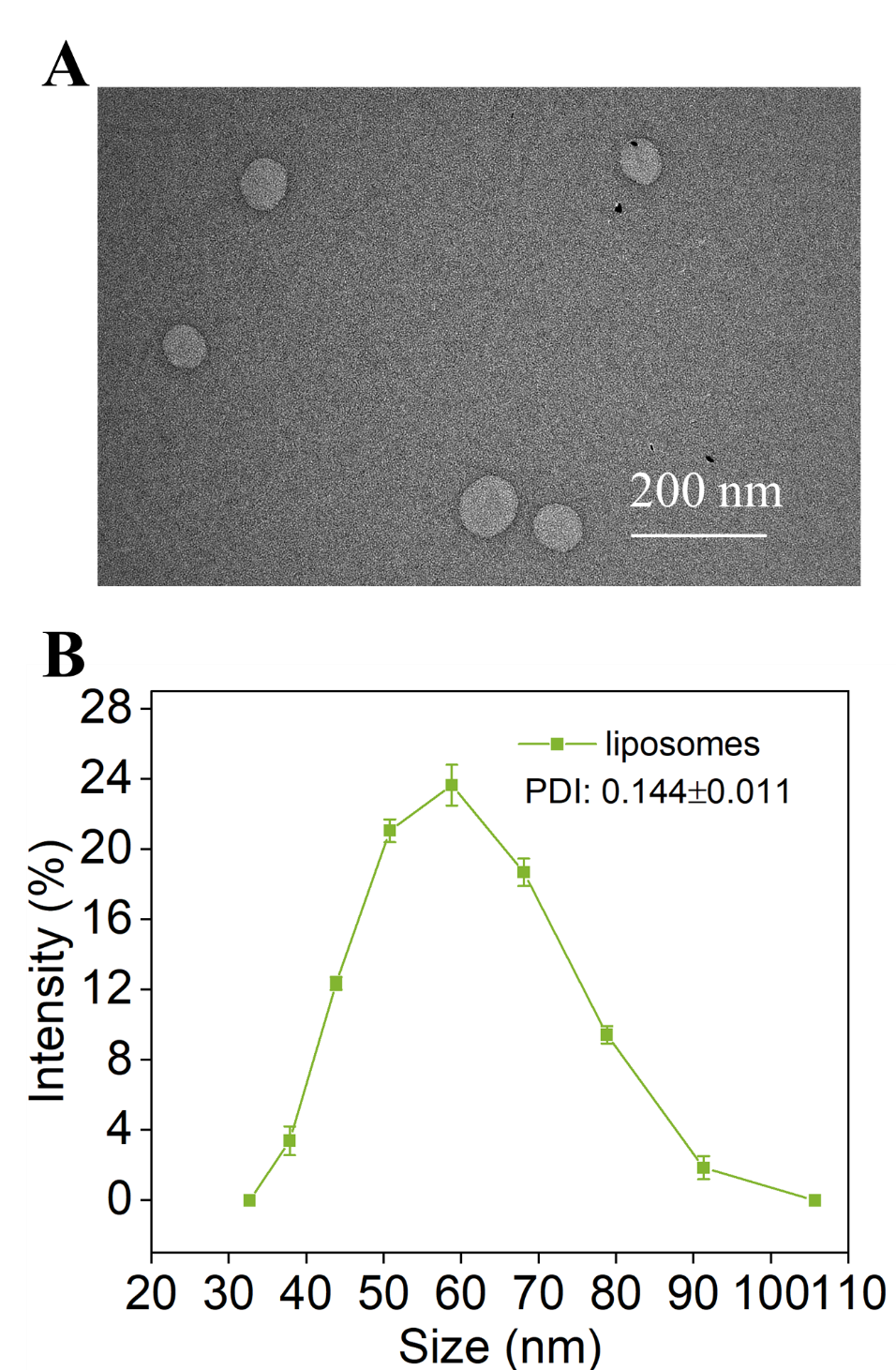


Figure S1. The TEM image (A) and size distribution (B) of liposomes.


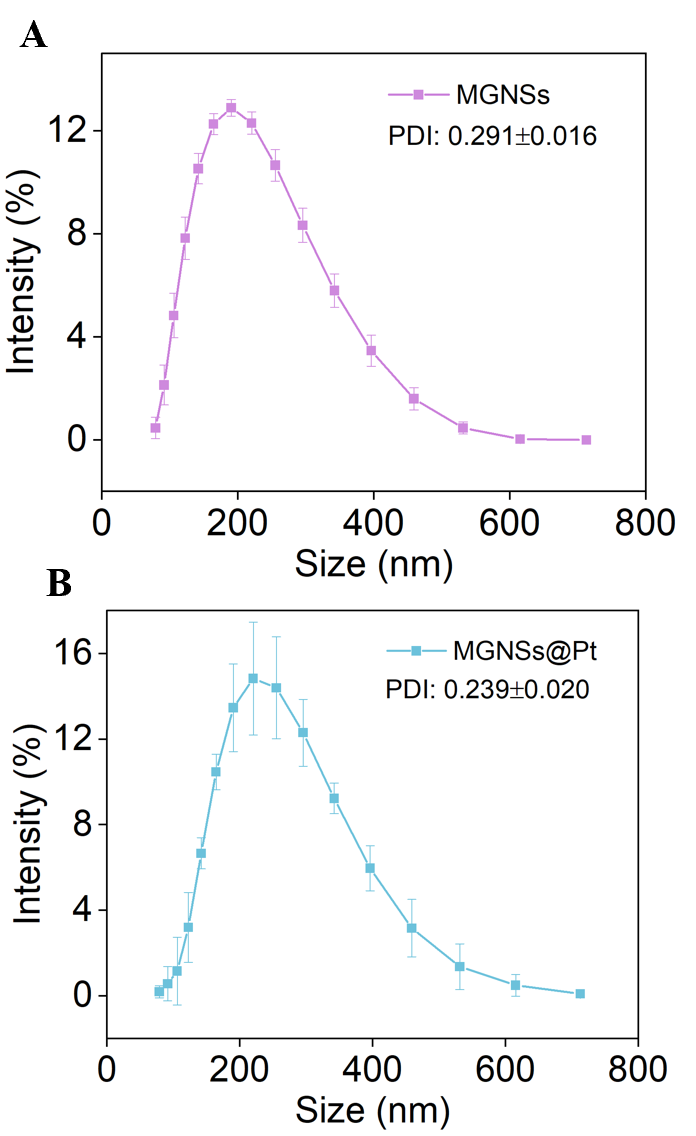


Figure S2. The size distribution of MGNSs and MGNSs@Pt measured by dynamic light scattering.

The results suggested that the average diameter of MGNSs was 210.0 ± 9.6 nm and PDI data was 0.291 ± 0.016. The average diameter of MGNSs@Pt was 232.8 ± 7.2 nm and PDI data was PDI: 0.239 ± 0.020.


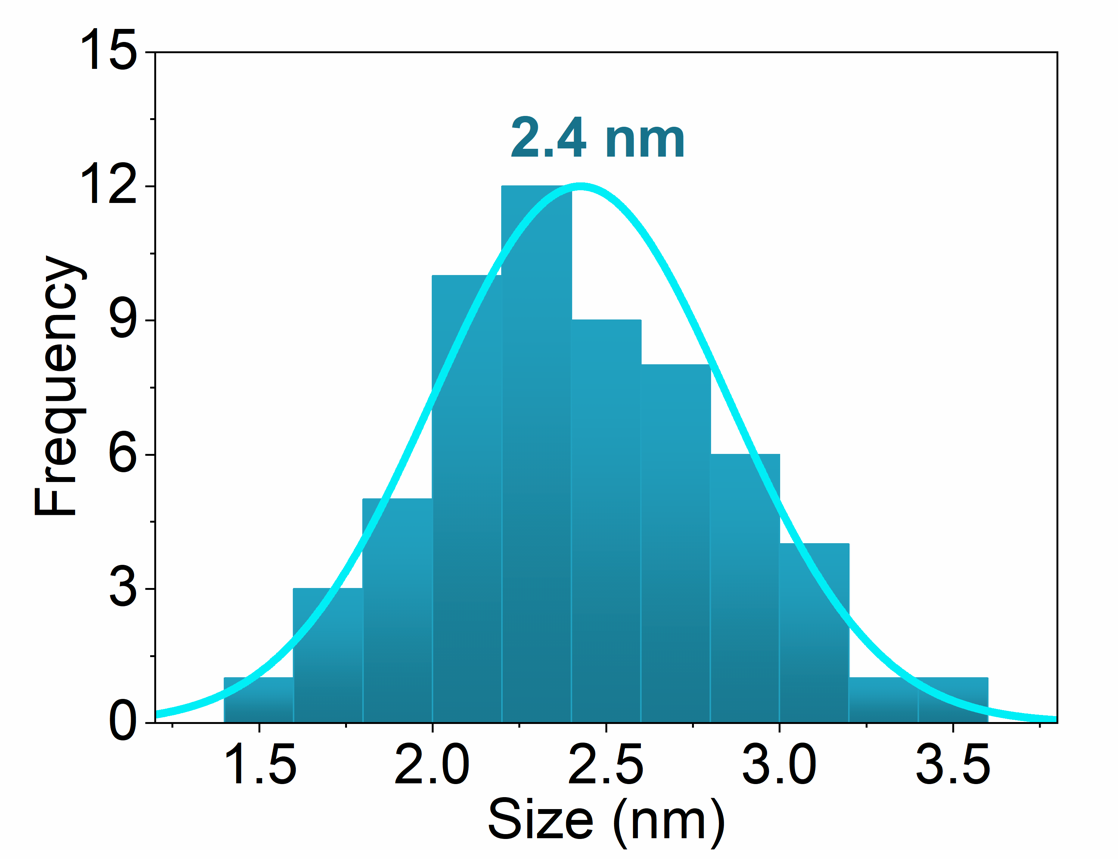


Figure S3. The size distribution of Pt nanoclusters on MGNSs@Pt.


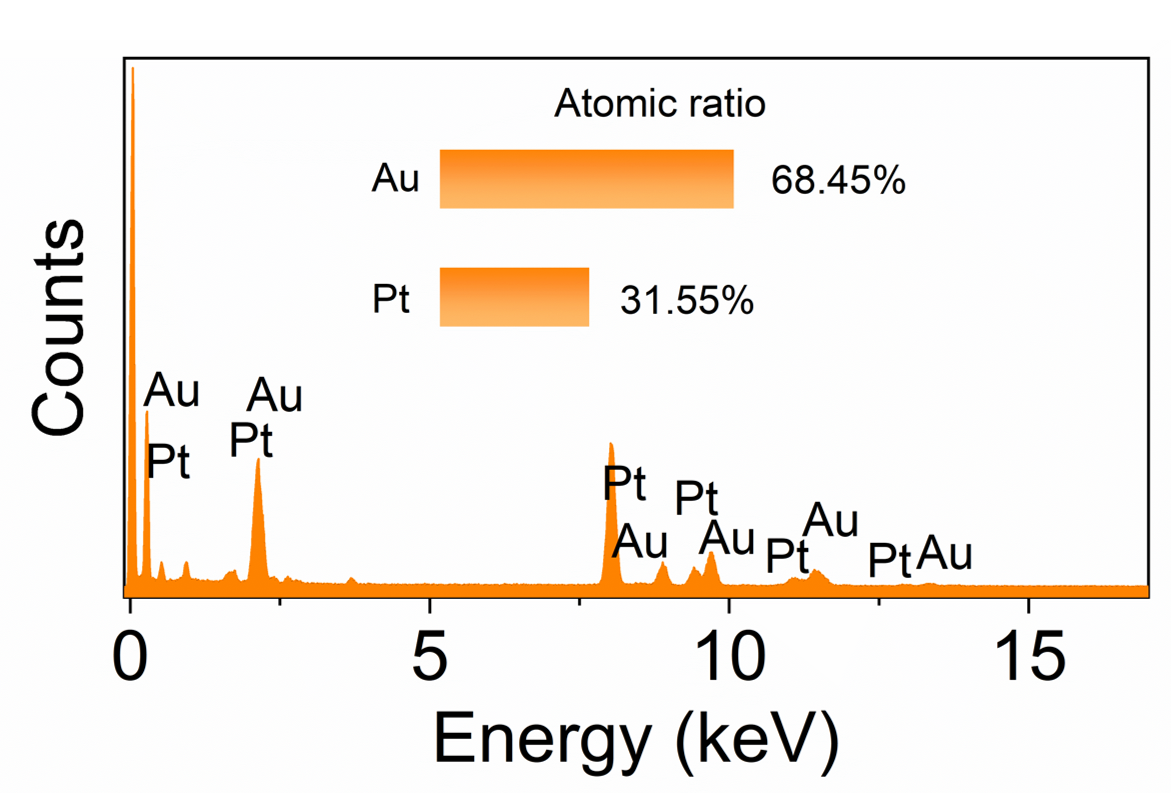


Figure S4. EDS spectroscopy of MGNSs@Pt. From EDS data, the doping efficiency of Pt in the MGNSs@Pt is calculated to be 94.7%.


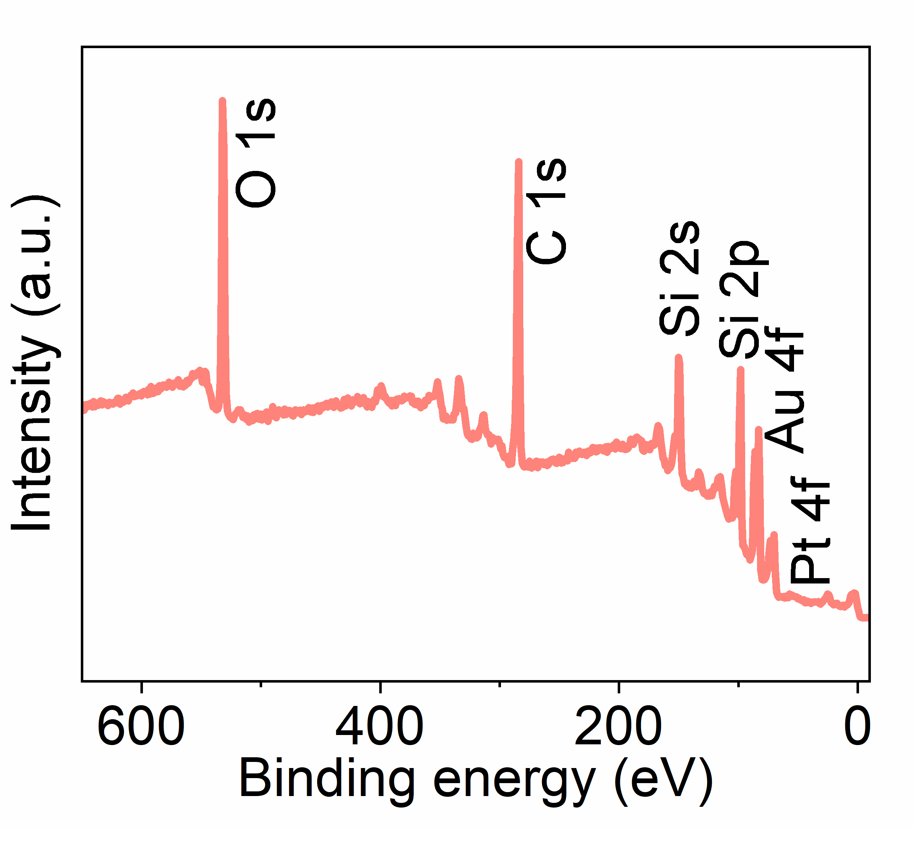


Figure S5. X-ray photoelectron spectroscopy (XPS) survey scan of MGNSs@Pt. Si signal was contributed by the silicon-based substrate typically used for support and hold the MGNSs@Pt samples.


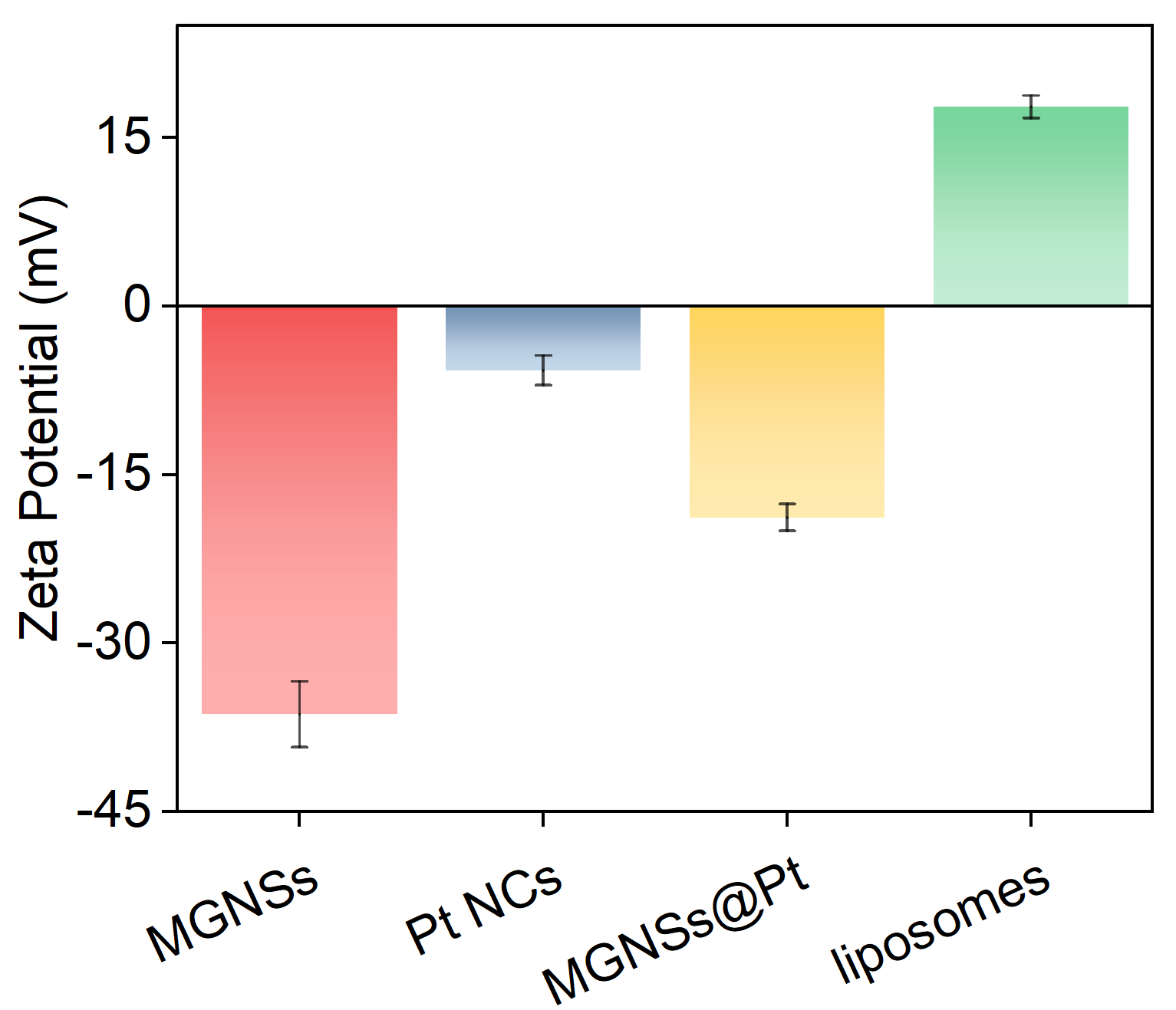


Figure S6. Zeta potential of MGNSs, Pt NCs, MGNSs@Pt and liposomes.


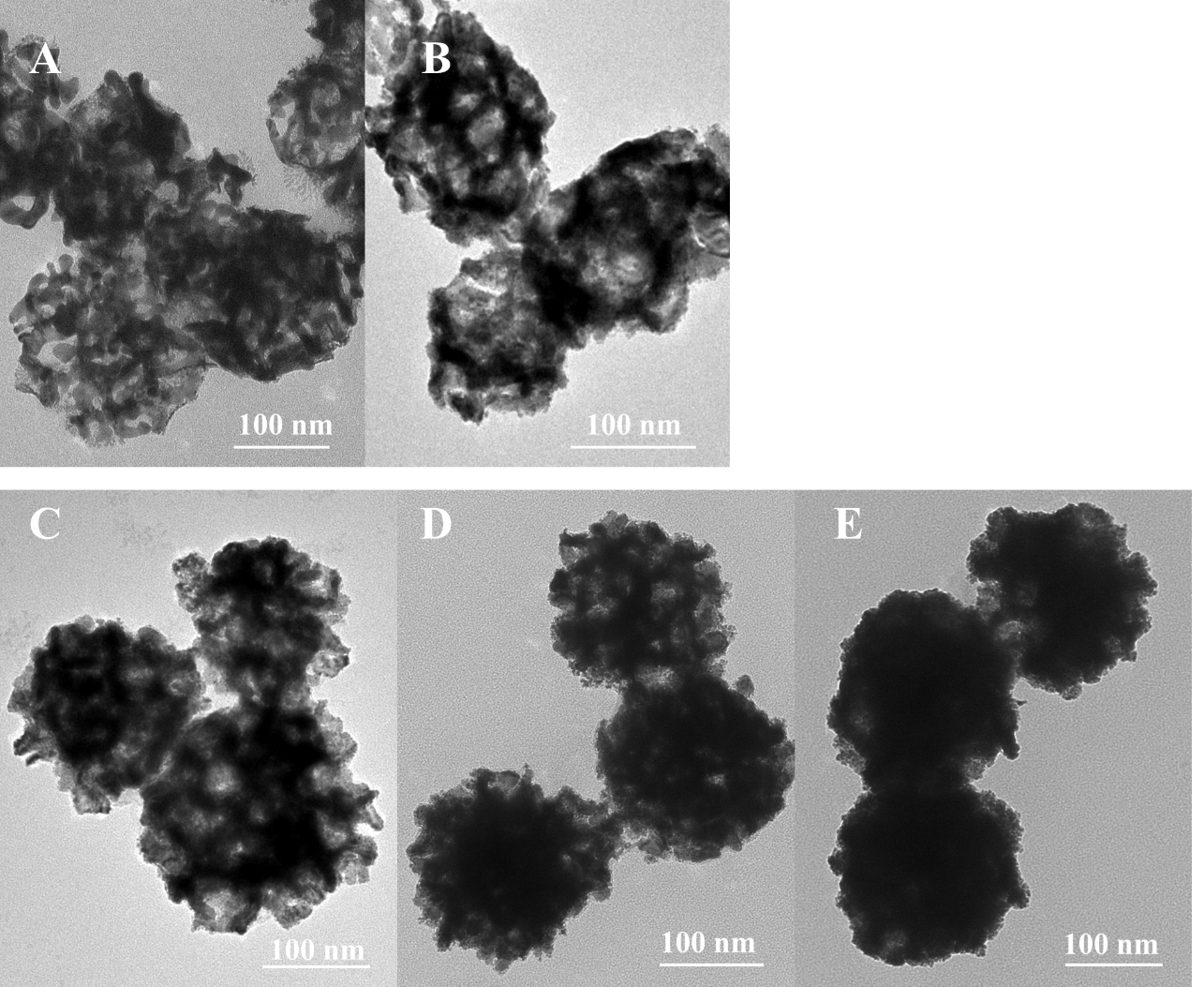


Figure S7. The TEM images of MGNSs@Pt with different Pt content. (A) 9 wt%, (B) 17 wt%, (C) 34 wt%, (D)50 wt% and (E) 67 wt%.


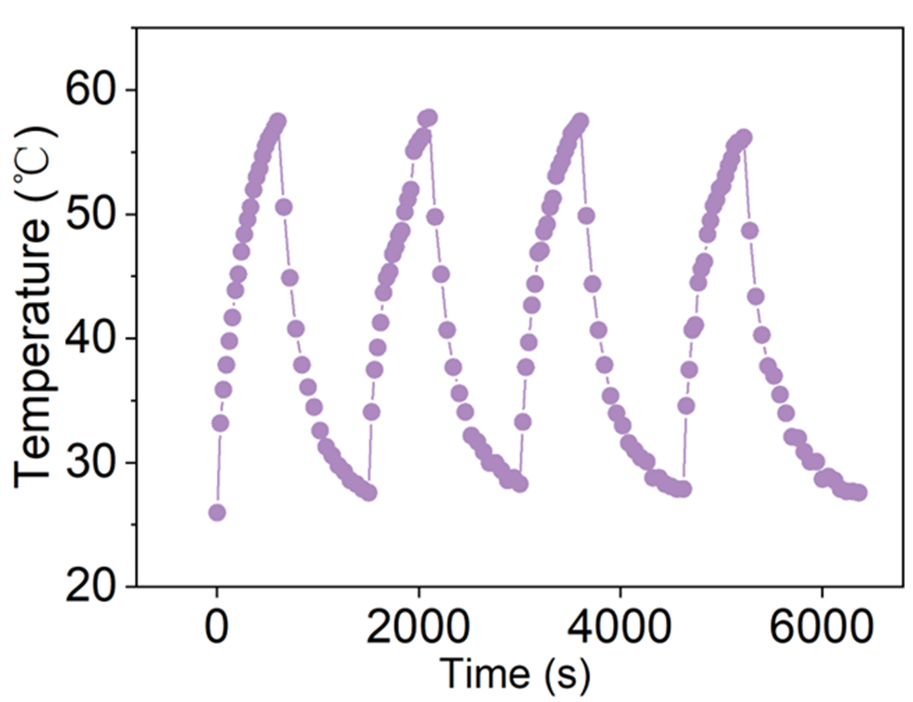


Figure S8. Photothermal stability evaluation of MGNSs under four on-off 1064 nm laser irradiation (100 μg mL^-1^, 2 W cm^−2^).


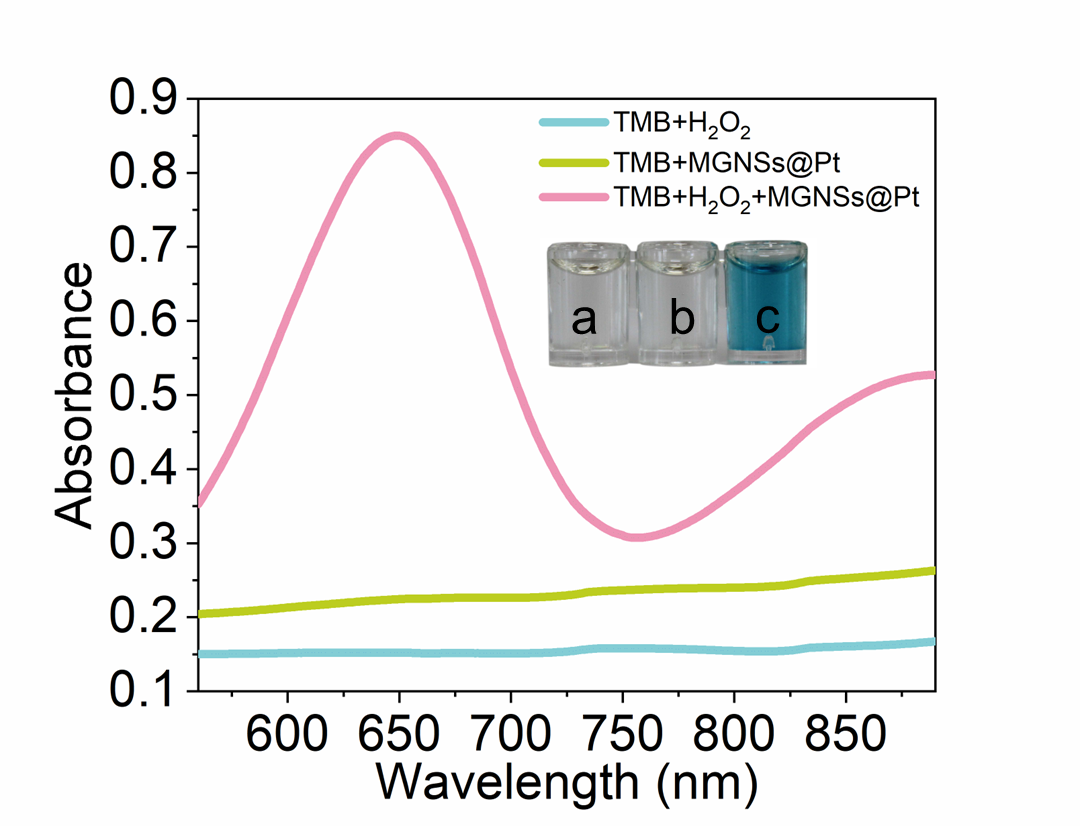


Figure S9. UV−vis spectra of three reaction solutions. Inserts: the color change of (a) TMB+H_2_O_2_, (b) TMB+MGNSs@Pt and (c) TMB+H_2_O_2_+ MGNSs@Pt.


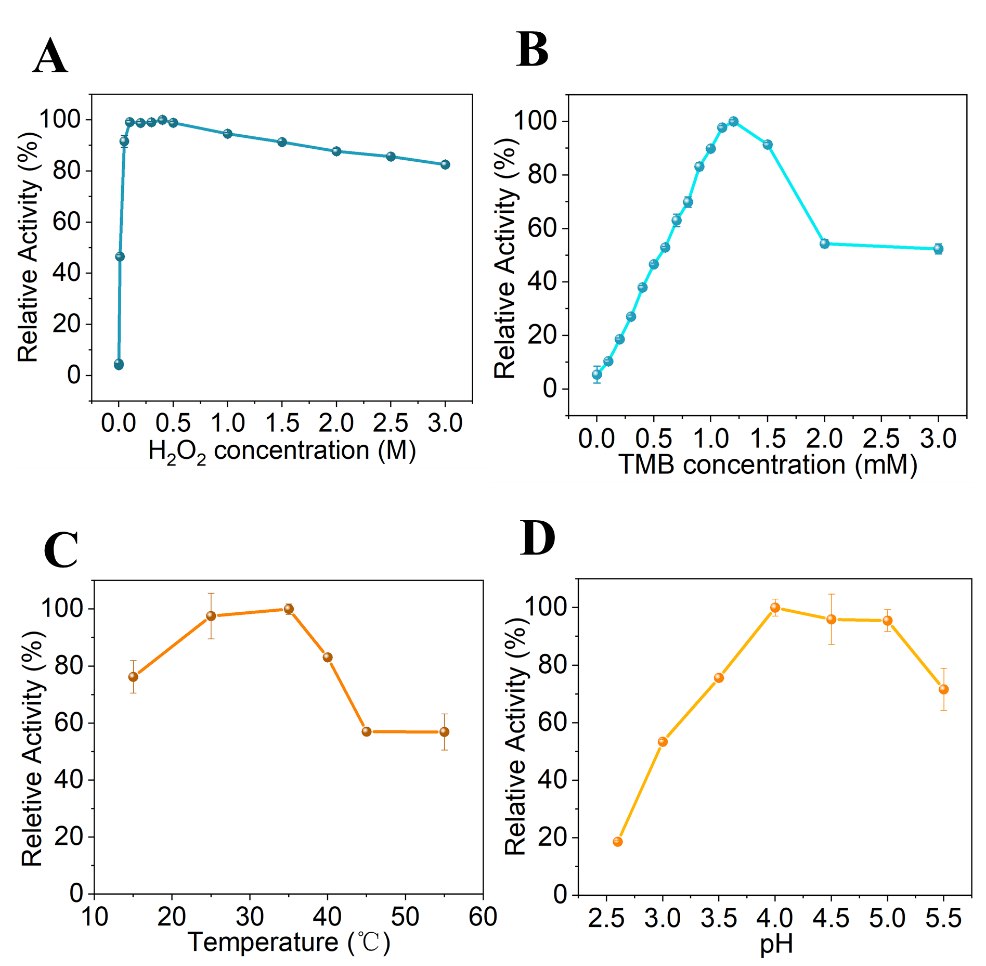


Figure S10. Effect of different conditions on the peroxidase-like activity of MGNSs@Pt: (A) TMB concentration, (B) H₂O₂ concentration, (C) temperature, and (D) pH. The optimal concentrations of TMB and H₂O₂ were 1.1 mM and 0.2 M, respectively


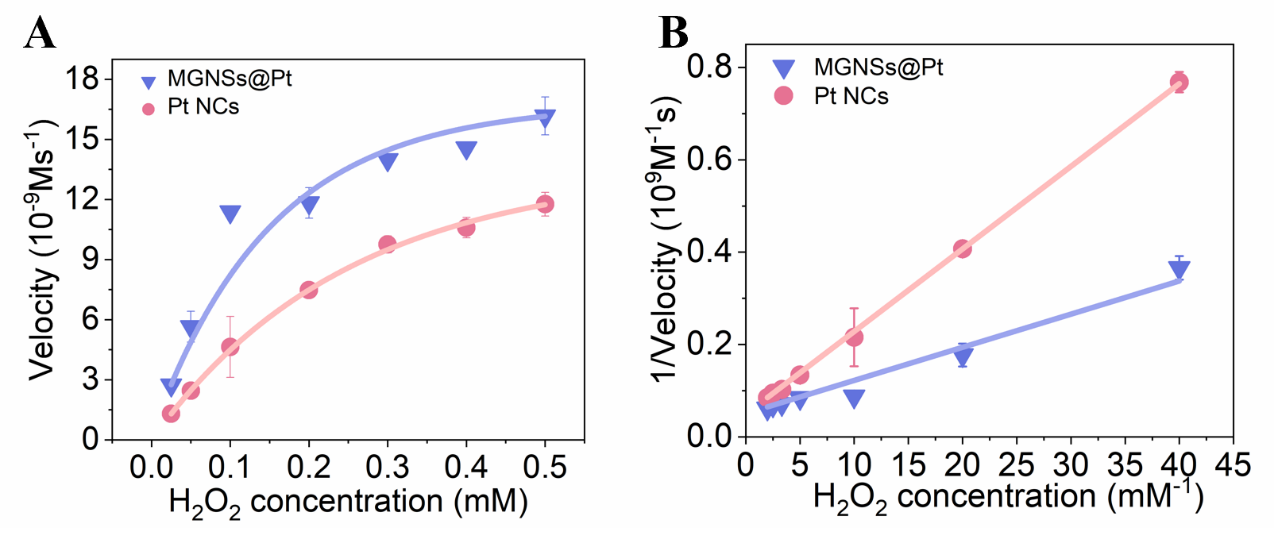


Figure S11. (A) Steady-state kinetic assay of MGNSs@Pt and Pt NCs by varying concentrations of H_2_O_2_ (0, 0.1,0.2,0.3,0.4, 0.5 mM). (B) Double-reciprocal plots of (A).


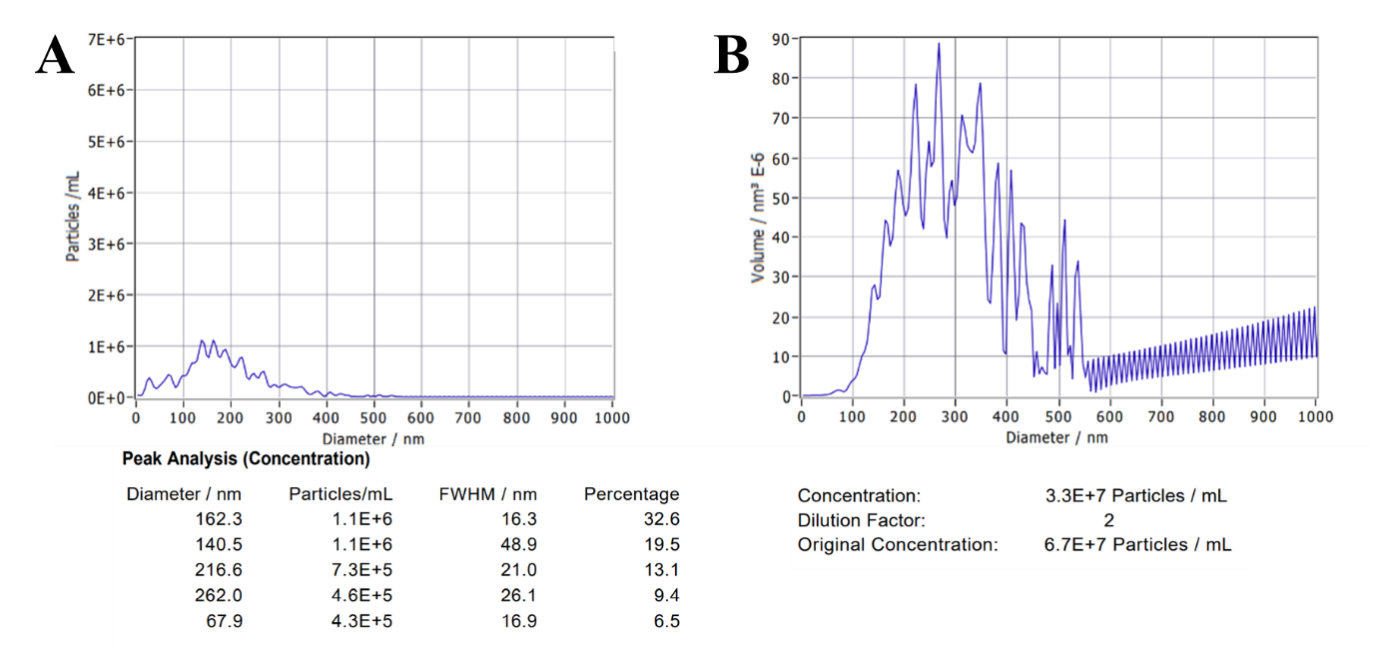


Figure S12. Particle concentration of MGNSs@Pt determined by nanoparticle tracking analysis (NTA) for calculation of K_cat_ value. The final concentration was 6.7×10^7^ Particles mL^-1^, corresponding to 0.5 μg mL^-1^ of MGNSs@Pt solution.


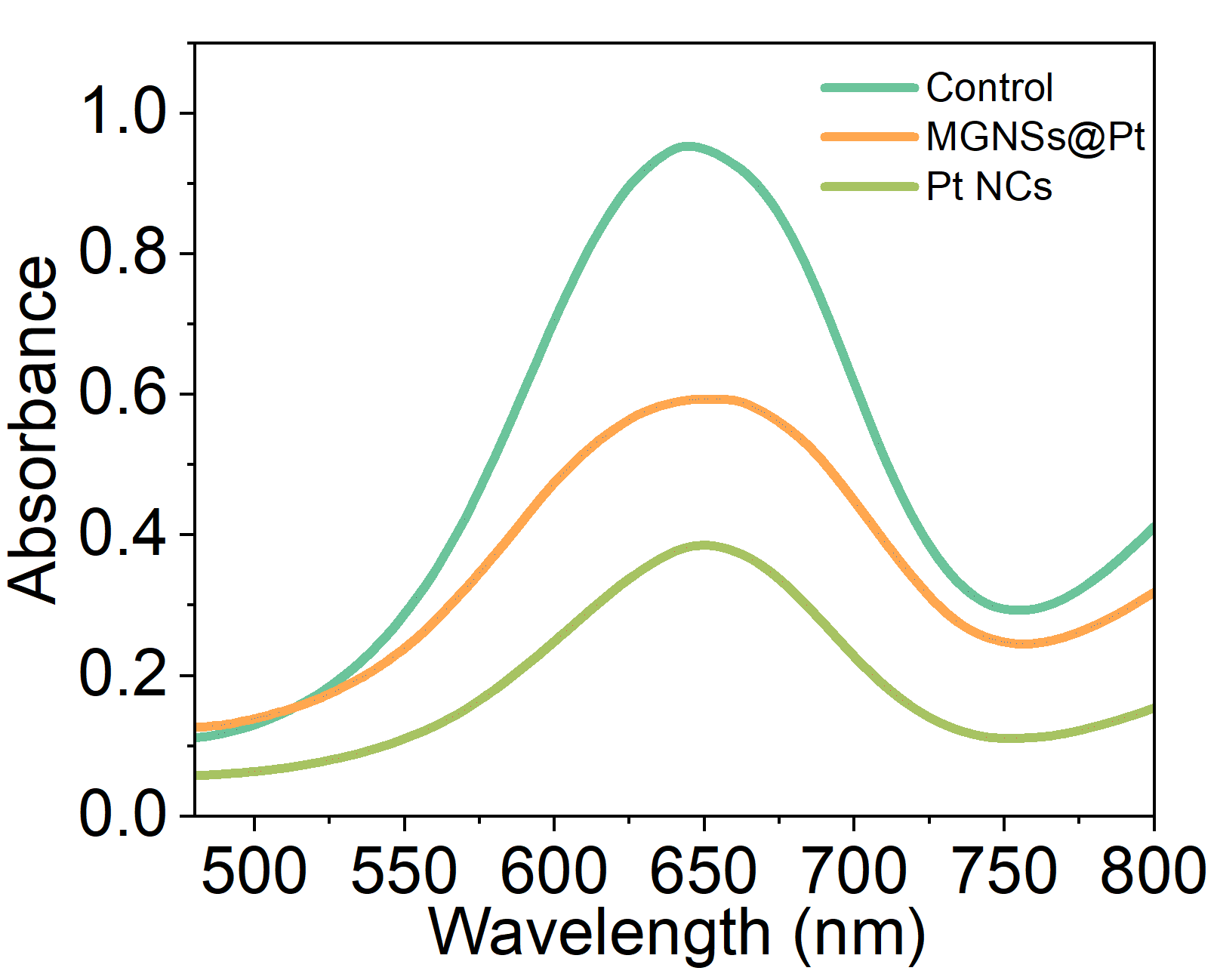


Figure S13. Absorption changes for the system of TMB, H_2_O_2_ with MGNSs@Pt and Pt NCs using isopropanol as scavenger for ·OH radicals.


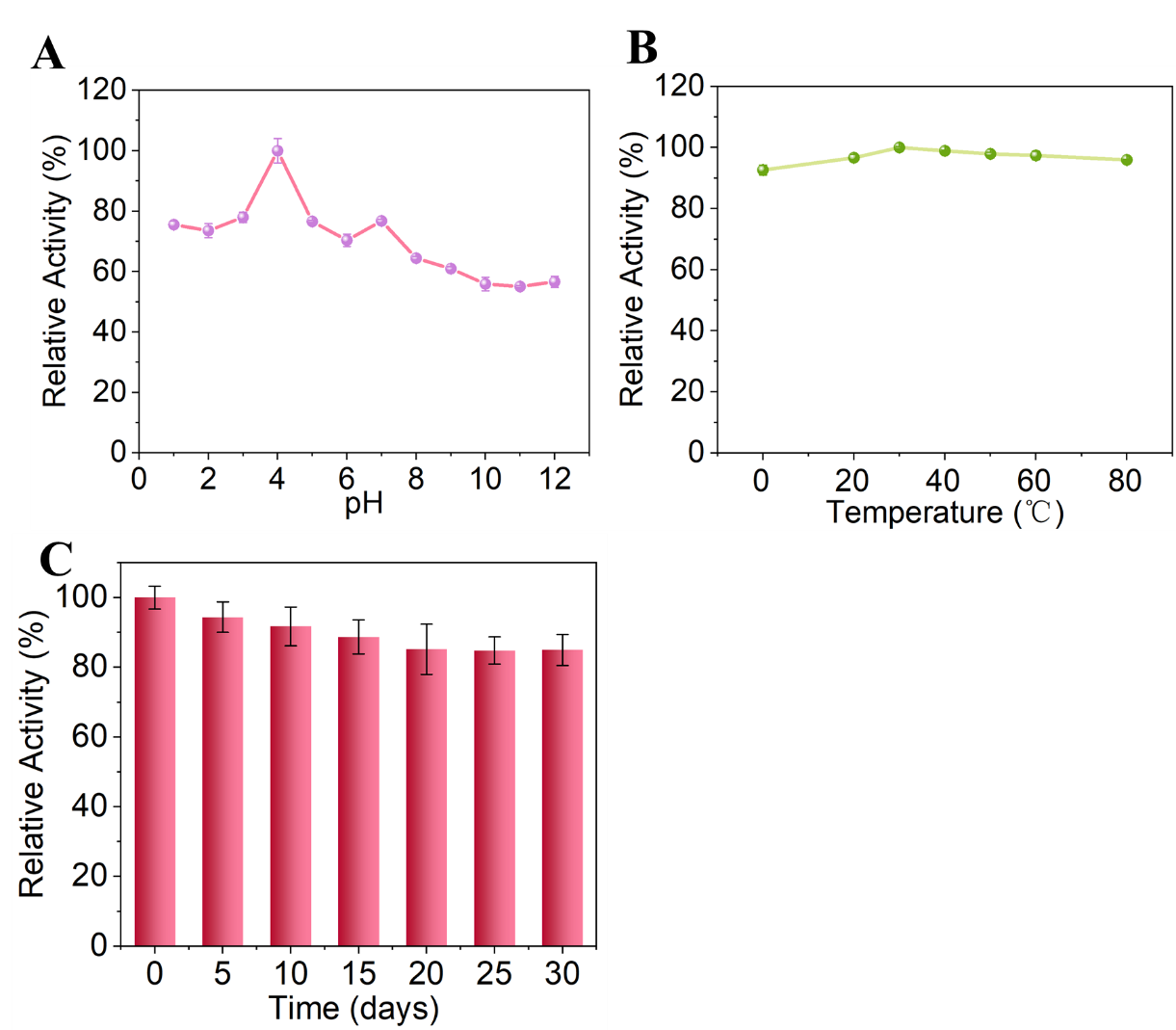


Figure S14. The effect of storage condition on the POD-like activity of MGNSs@Pt (A and B). MGNSs@Pt was stored in the different pH values ranging from 1 to 12 using sodium hydroxide (NaOH) and hydrochloric acid (HCl) and stored in different temperature for 2 h. MGNSs@Pt could be maintained at high catalytic activity in the wide pH (1-12) and temperature range (0-80 ℃). (C) The POD-like activity of MGNSs@Pt under different storage time. The POD-like activity of MGNSs@Pt dispersed in deionized water solution still maintained 85% at 4 ℃ after 30 d (pH = 6.5).


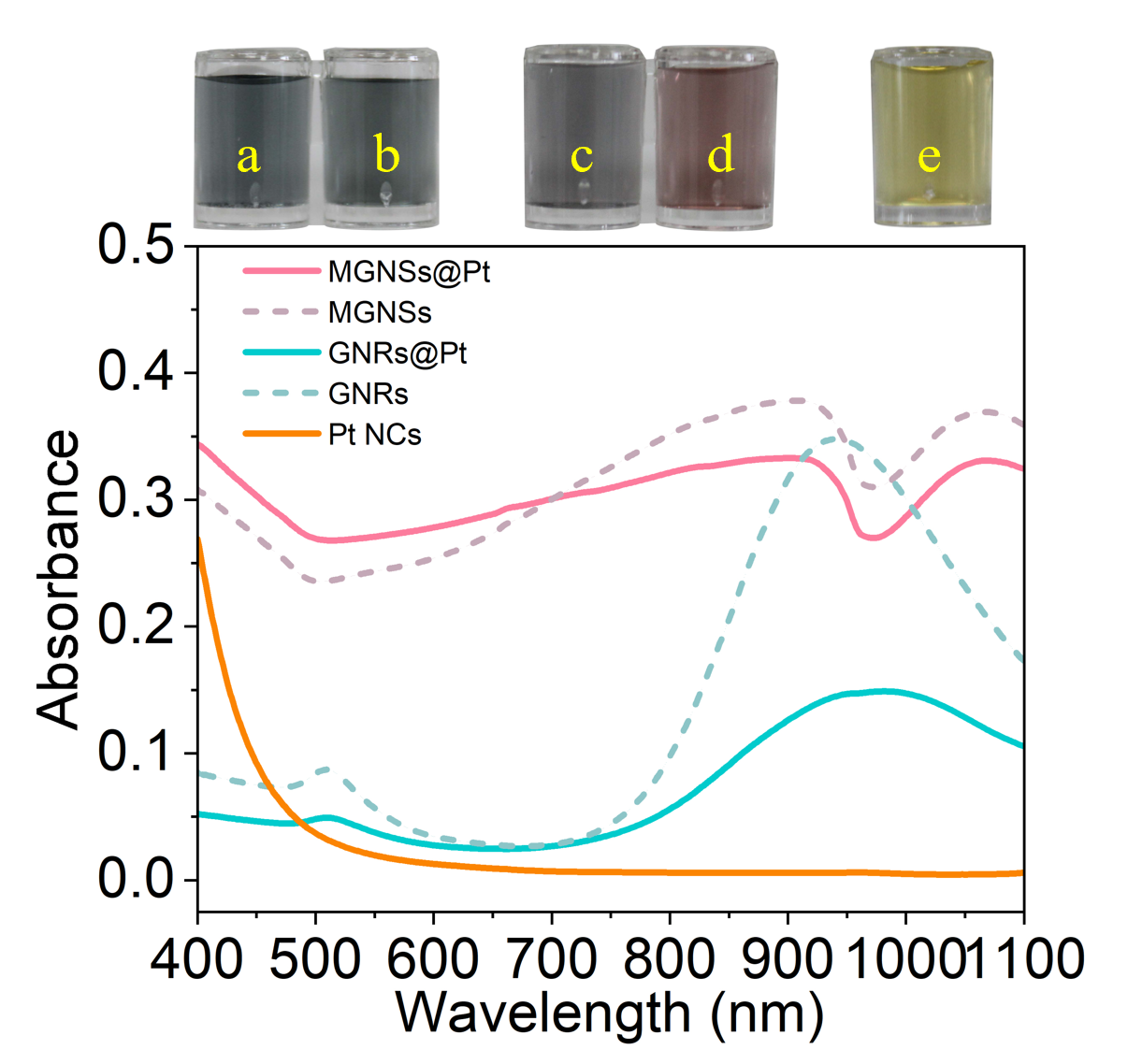


Figure S15. (A) UV-vis spectra of the different nanostructure. Insert: the corresponding digital pictures of (a) MGNSs@Pt, (b) MGNSs, (c) GNRs@Pt, (d) GNRs and (e) Pt NCs.


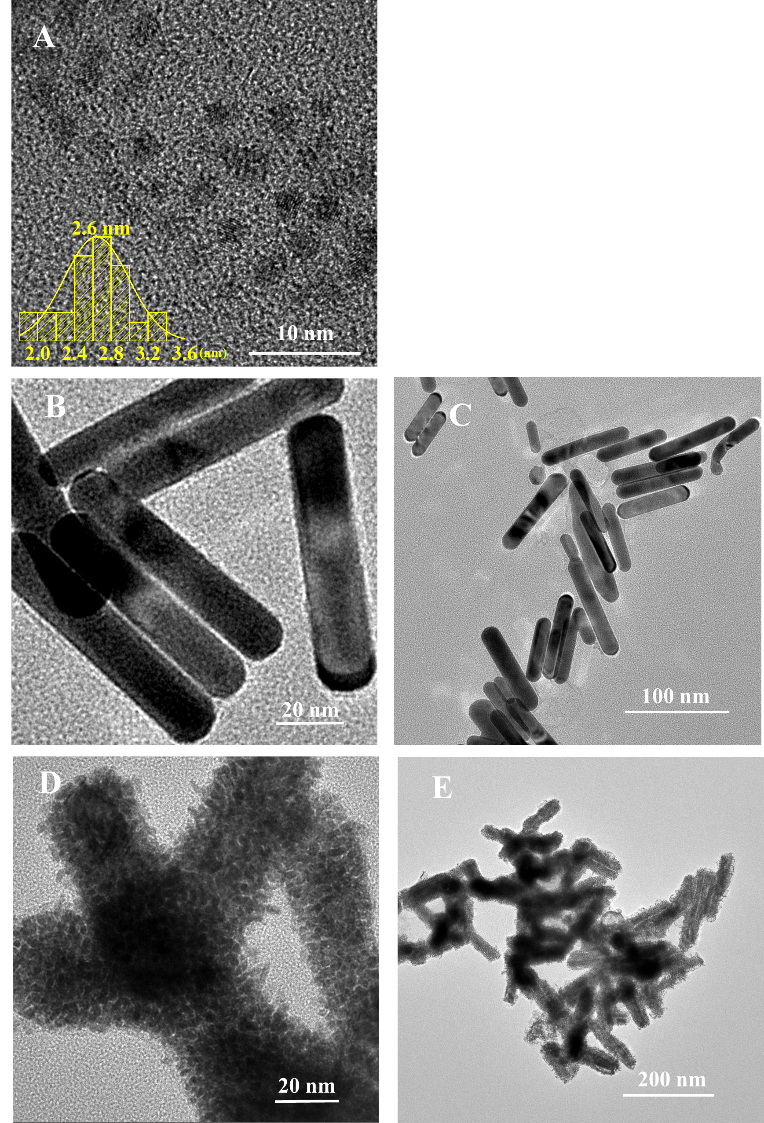


Figure S16. TEM images of different nanostructures. (A) Pt NCs (average particle size of 2.6 nm), (B, C) GNRs (the diameter of 17.2 nm and the aspect ratio of 5.0), (D, E) GNRs@Pt.


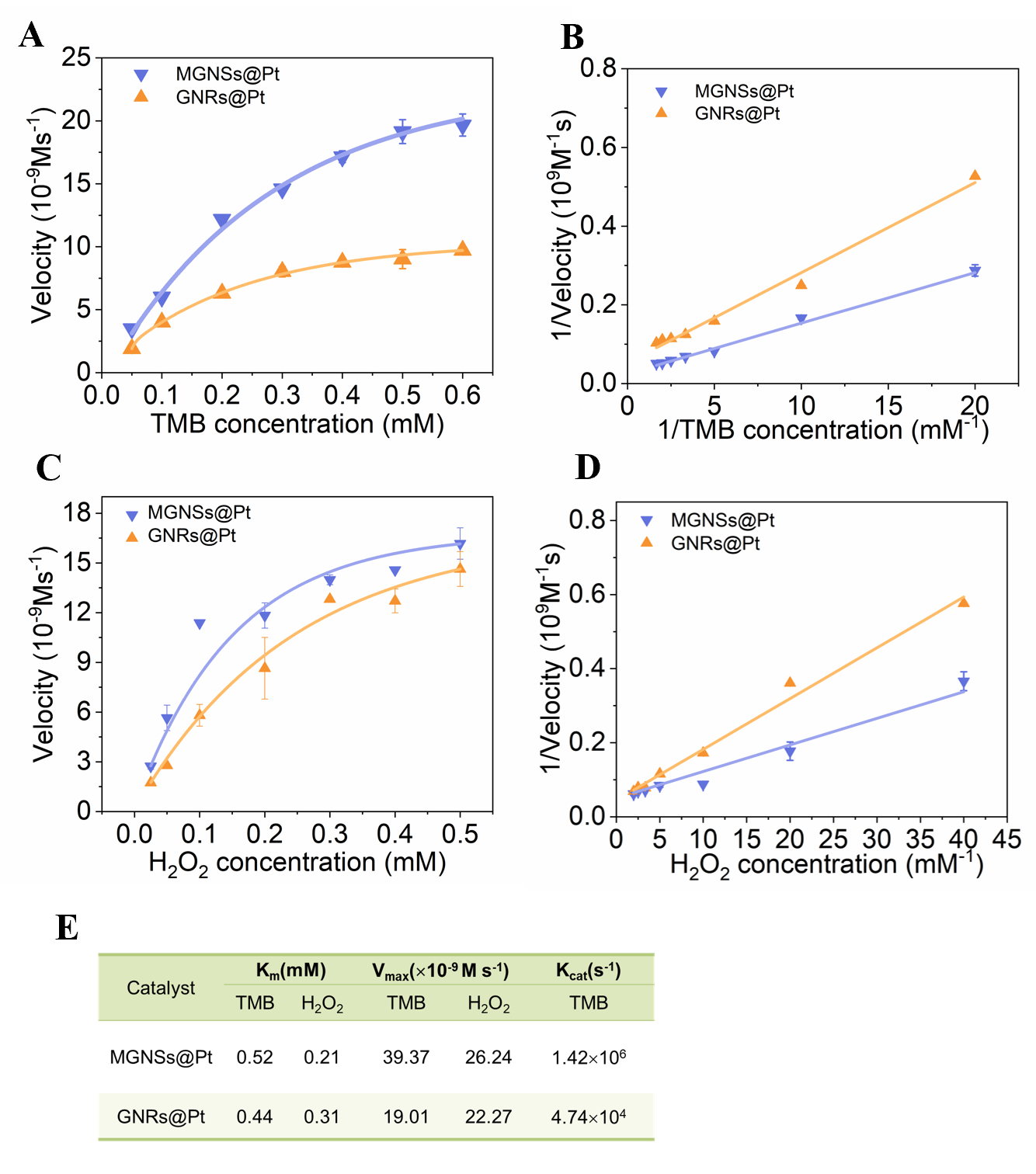


Figure S17. (A) Steady-state kinetic assay of MGNSs@Pt and GNRs@Pt by varying concentrations of TMB. (B) Double-reciprocal plots of (A). (C) Steady-state kinetic assay of MGNSs@Pt and GNRs@Pt by varying concentrations of H_2_O_2_. (D) Double-reciprocal plots of (C). (E) Comparison of the Michaelis−Menten constant (K_m_) and the Maximum Reaction Velocity (V_max_) among MGNSs@Pt and GNRs@Pt.


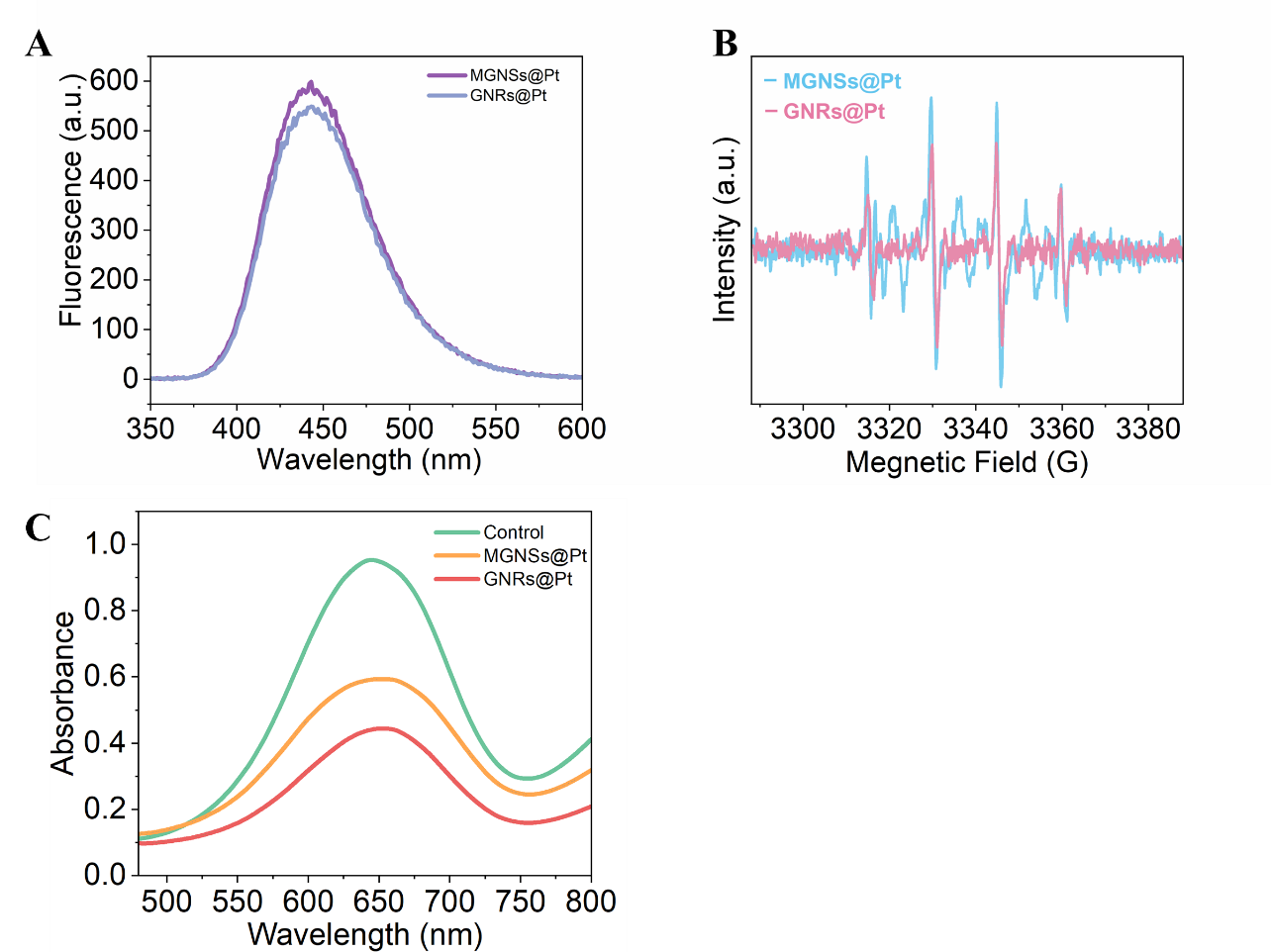


Figure S18. (A) Fluorescence spectra for the system of 0.5 mM TA, 50 mM H_2_O_2_ with MGNSs@Pt and GNRs@Pt (100 μg mL^-1^) at pH 6.5. (B) ESR spectra of 50 mM DMPO, 50 mM H_2_O_2_ with MGNSs@Pt and GNRs@Pt (100 μg mL^-1^) at pH 6.5. (C) Absorption changes for the system of TMB, H_2_O_2_ with MGNSs@Pt and GNRs@Pt using isopropanol as scavenger for ·OH radicals.


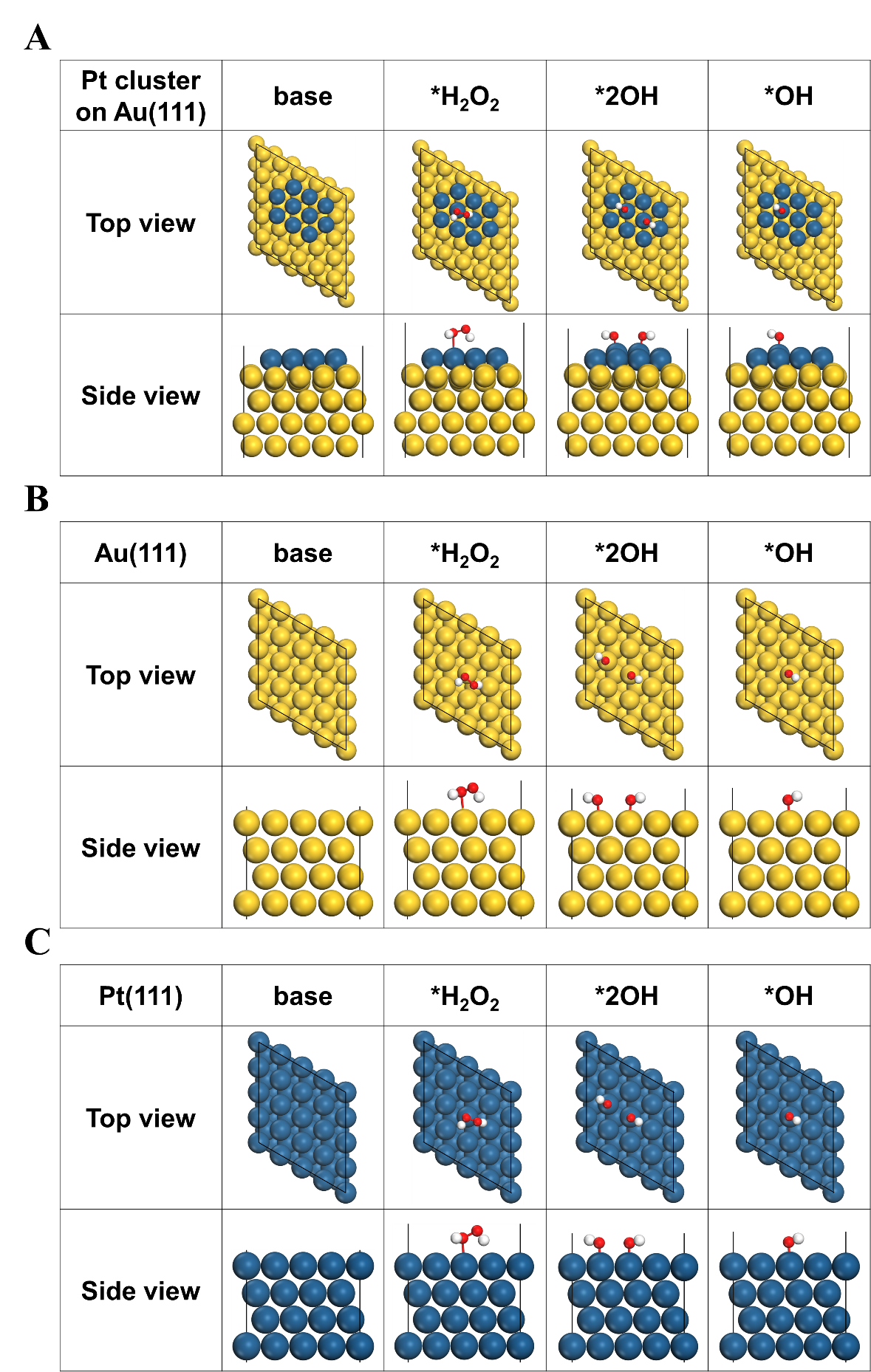


Figure S19. The optimized atomic structures of adsorbed intermediates during peroxidase-like reaction on (A) MGNSs@Pt, (B)MGNSs and (C) Pt NCs.

**
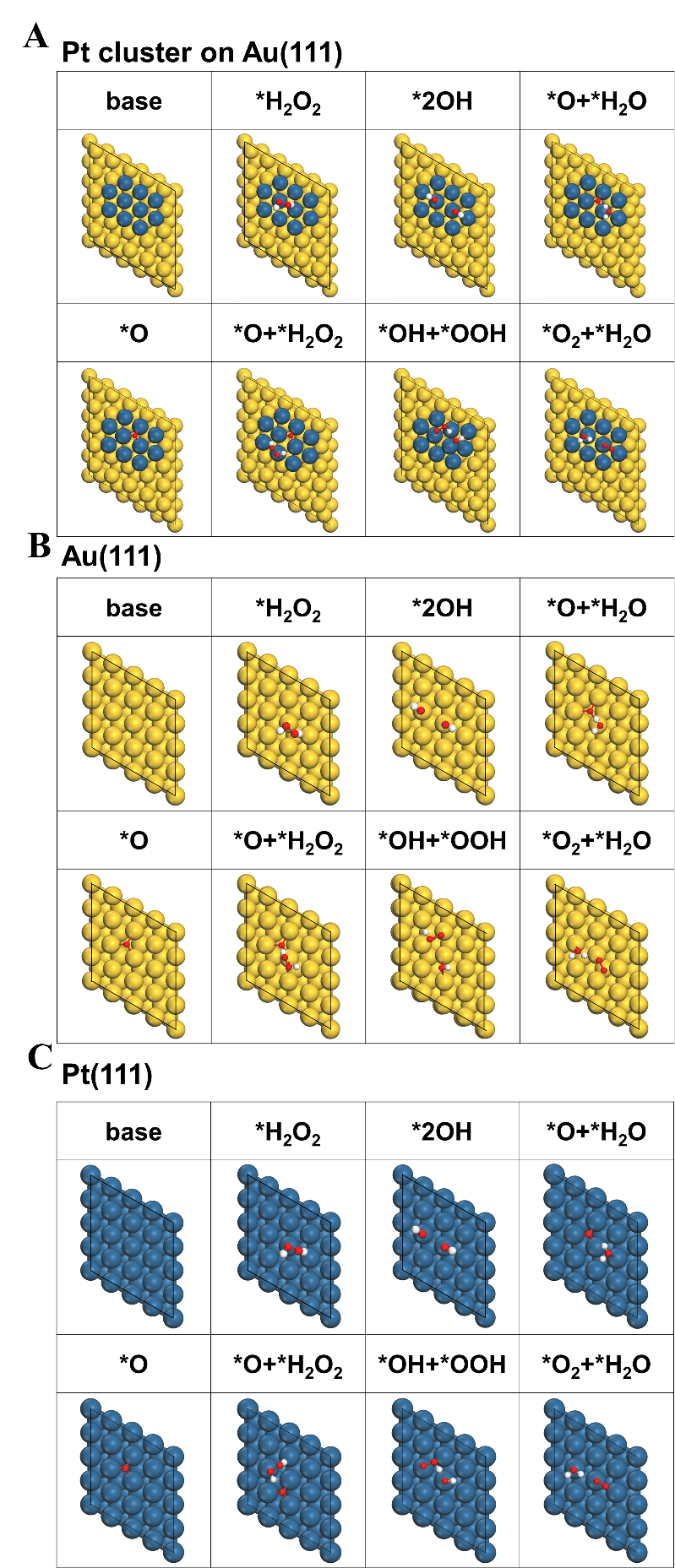
**

Figure S20. The optimized atomic structures of adsorbed intermediates during catalase-like reaction on (A) MGNSs@Pt, (B) MGNSs and (C) Pt NCs.


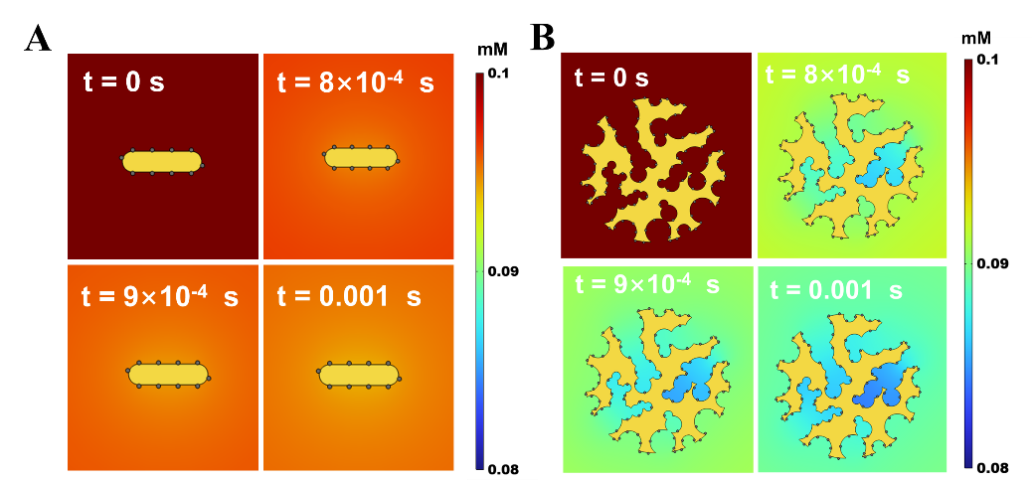


Figure S21. (A) Real-time H_2_O_2_ consumption of constructed GNRs@Pt models with initial concentration of 0.1 mM. (B) Real-time H_2_O_2_ consumption of constructed MGNSs@Pt models with initial H_2_O_2_ concentration of 0.1 mM.


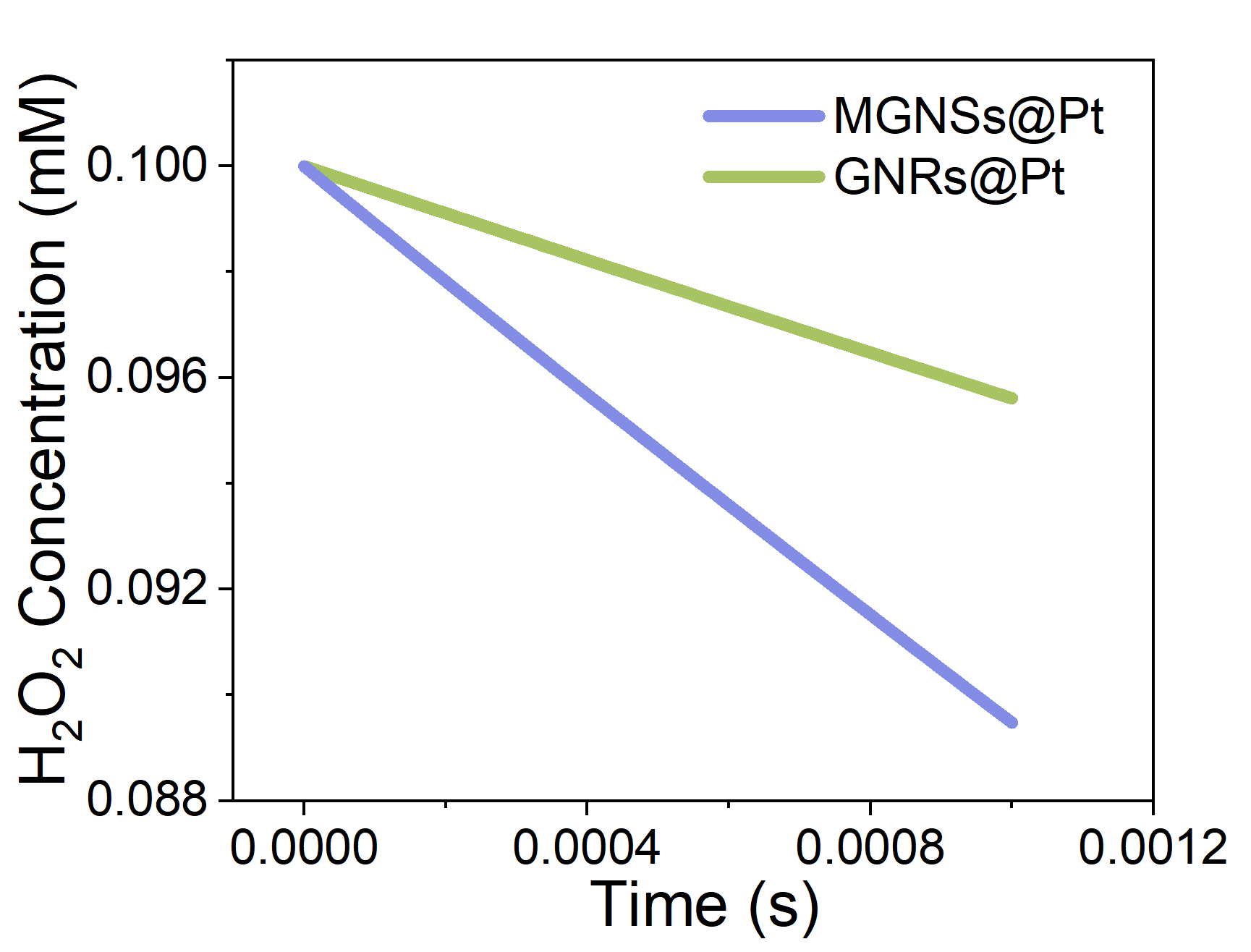


Figure S22. The simulated H_2_O_2_ consumption of MGNSs@Pt and GNRs@Pt along with time.


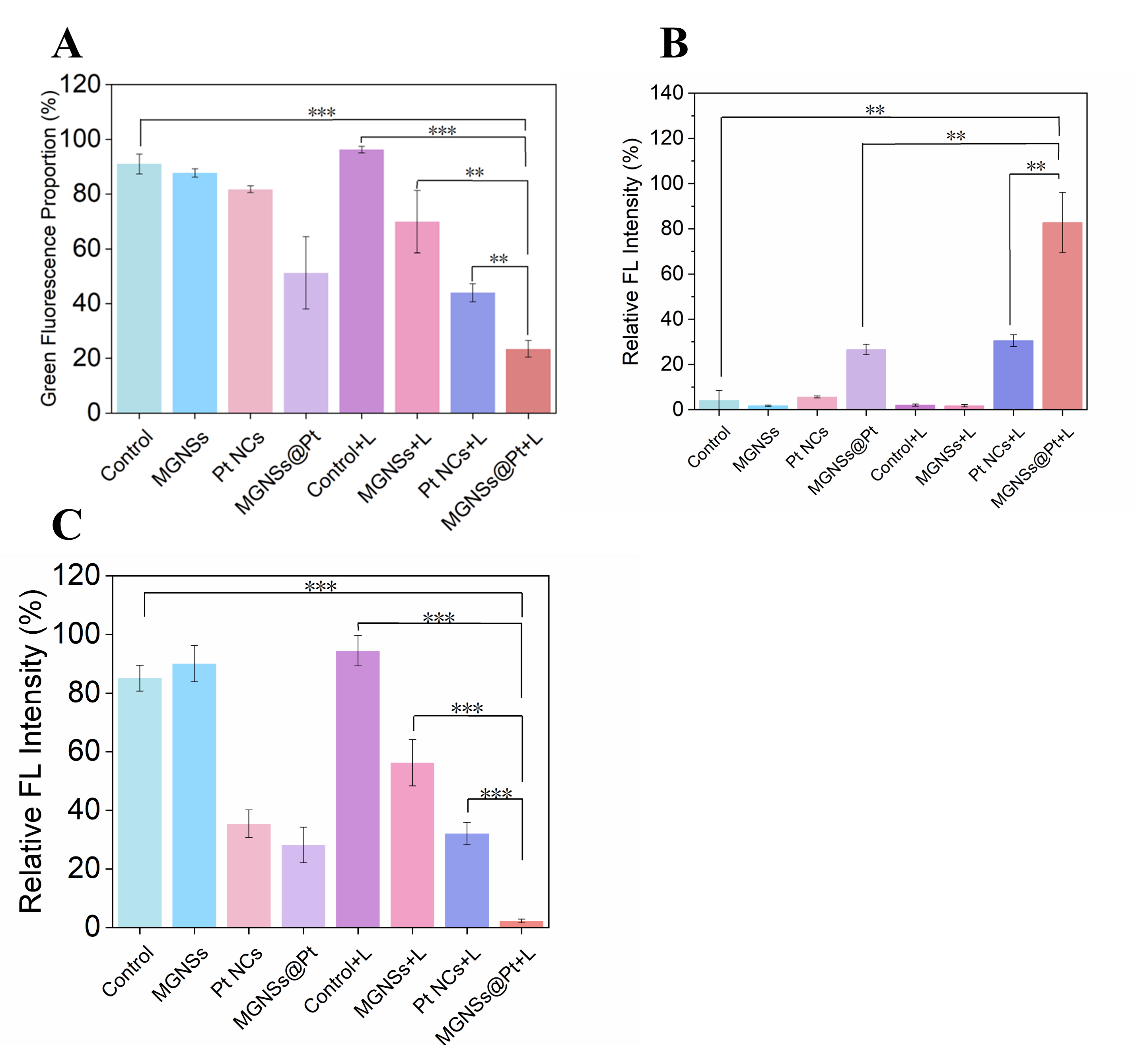


Figure S23. (A) Semi-quantitative analysis of green fluorescent proportion in live/dead-stained 4T1 cells. (B) Semi-quantitative analysis of relative fluorescent intensity of ROS in 4T1 cells. (C) Semi-quantitative analysis of relative red fluorescent intensity of intracellular O_2_ in 4T1 cells. Data are defined as mean ± SD (n = 3). Statistical significance is assessed by unpaired Student’s two-sided *t*-test, ***p*<0.01, ****p*<0.001.


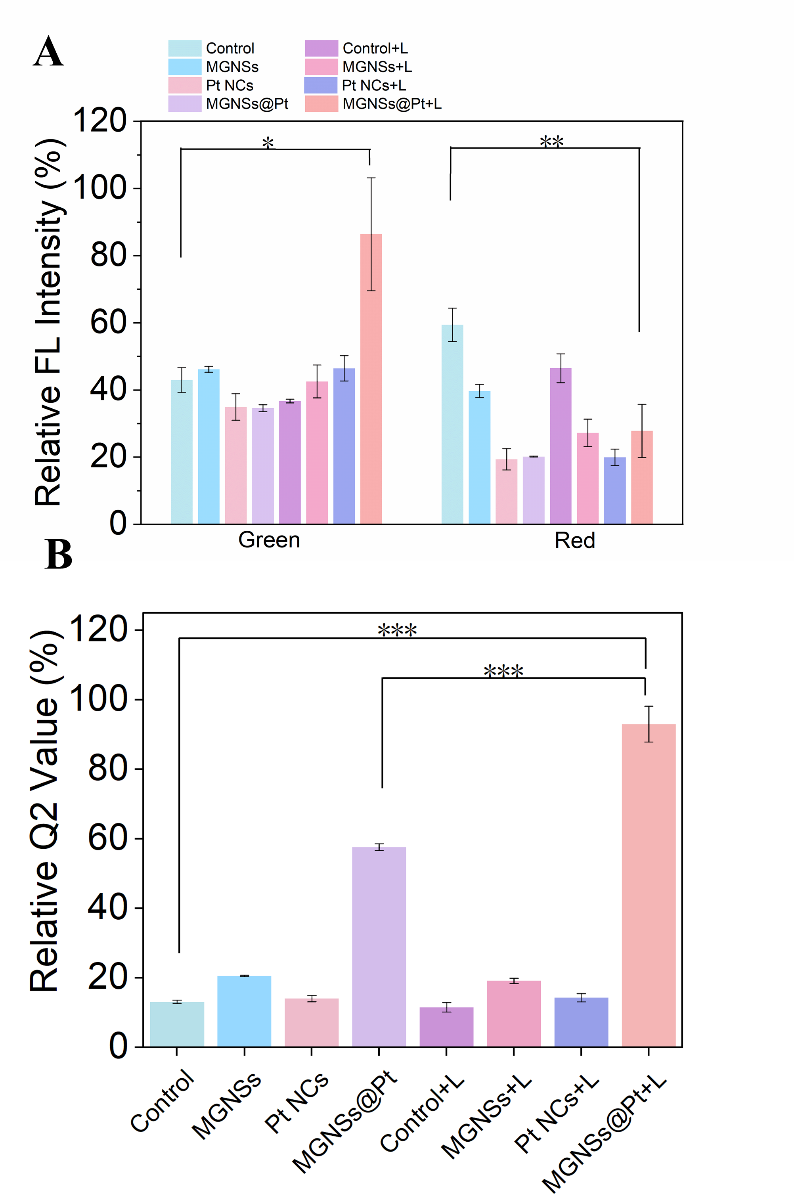


Figure S24. (A) Semi-quantitative analysis of relative green and red fluorescent intensity in JC-1-stained 4T1 cells. (B) Quantitative analysis of Q2 value in flow cytometry analysis of 4T1 cells. Data are defined as mean ± SD (n = 3). Statistical significance is assessed by unpaired Student’s two-sided *t*-test, **p*<0.05, ***p*<0.01, ****p*<0.001.


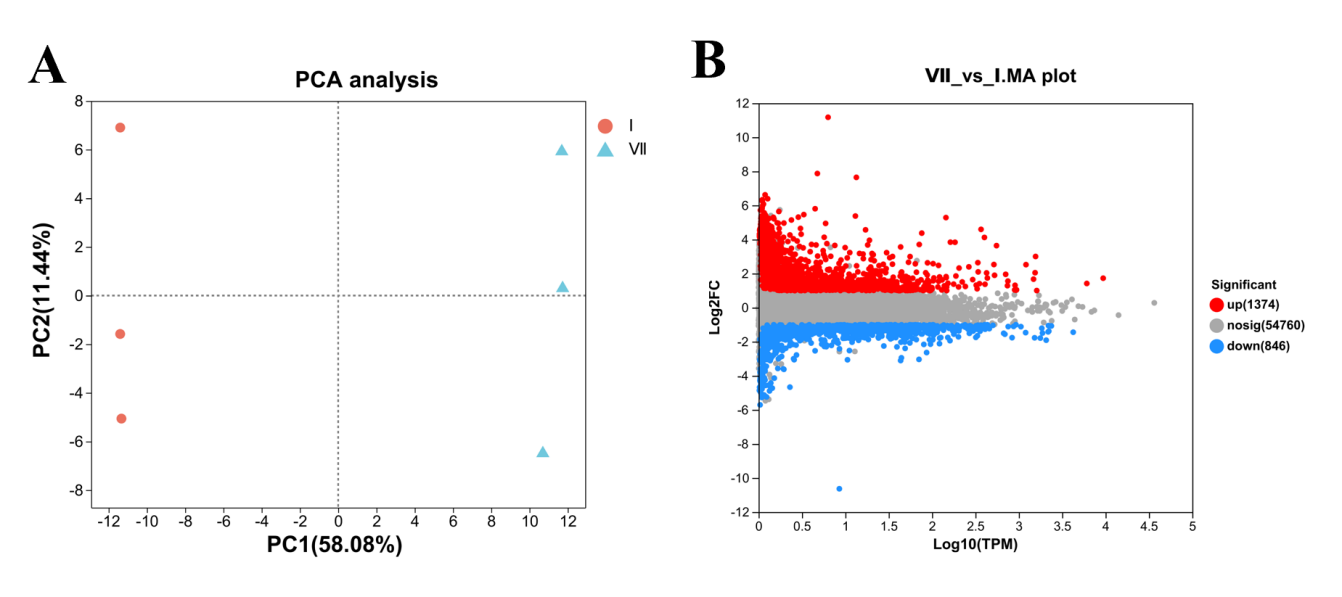


Figure S25. (A) Principal component analysis of differentially expressed genes in 4T1 cells treated MGNSs@Pt with laser irradiation and control cells (n = 6). (B) MA plot showing upregulated (red) and downregulated (blue) genes in MGNSs@Pt + L group compared to control group. Ⅰ: control, Ⅶ: MGNSs@Pt + L (n = 6).


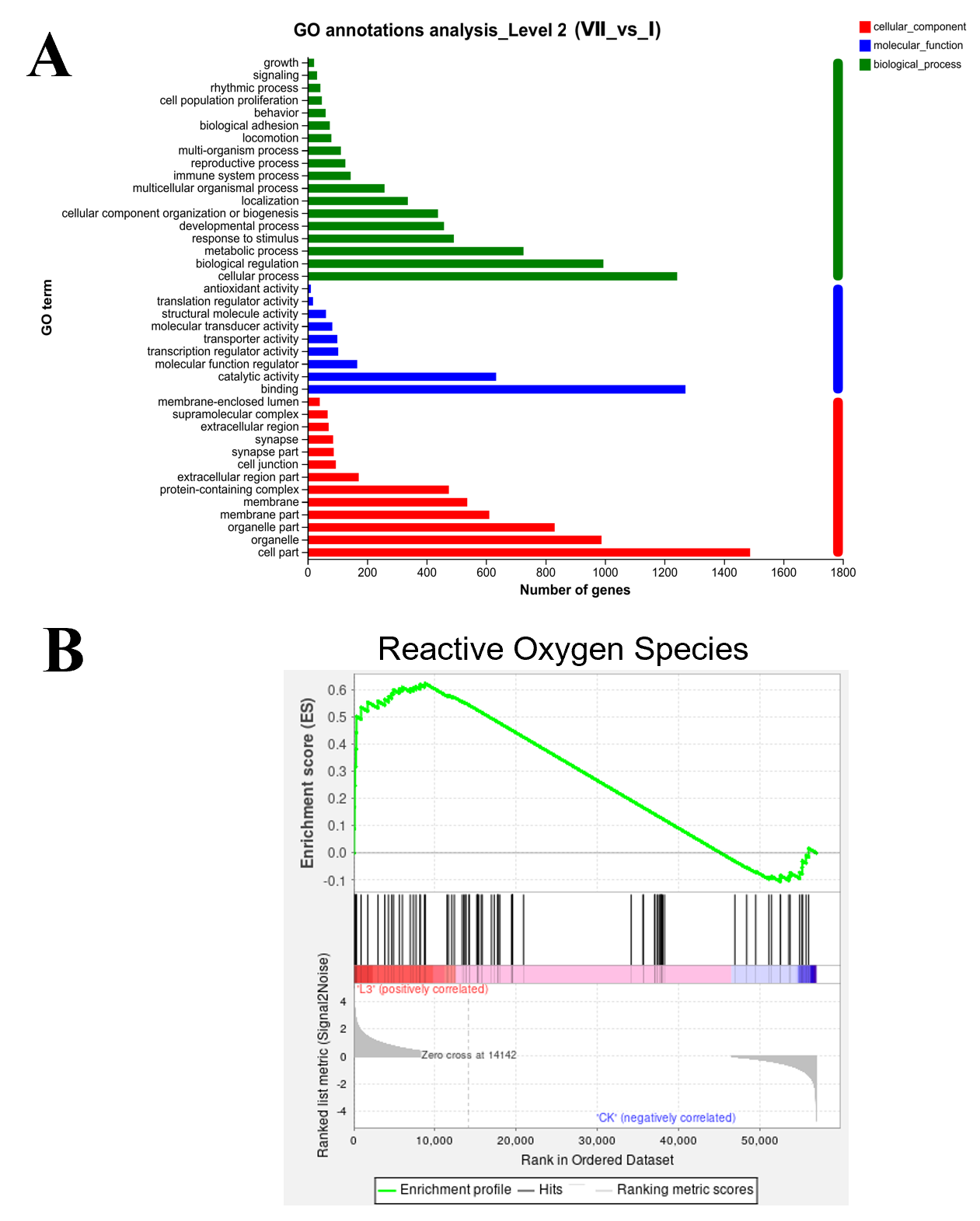


Figure S26. (A) GO and (B) GSEA enrichment analysis of differential gene expression in MGNSs@Pt + L treated 4T1 cells and the control cells (n = 6).


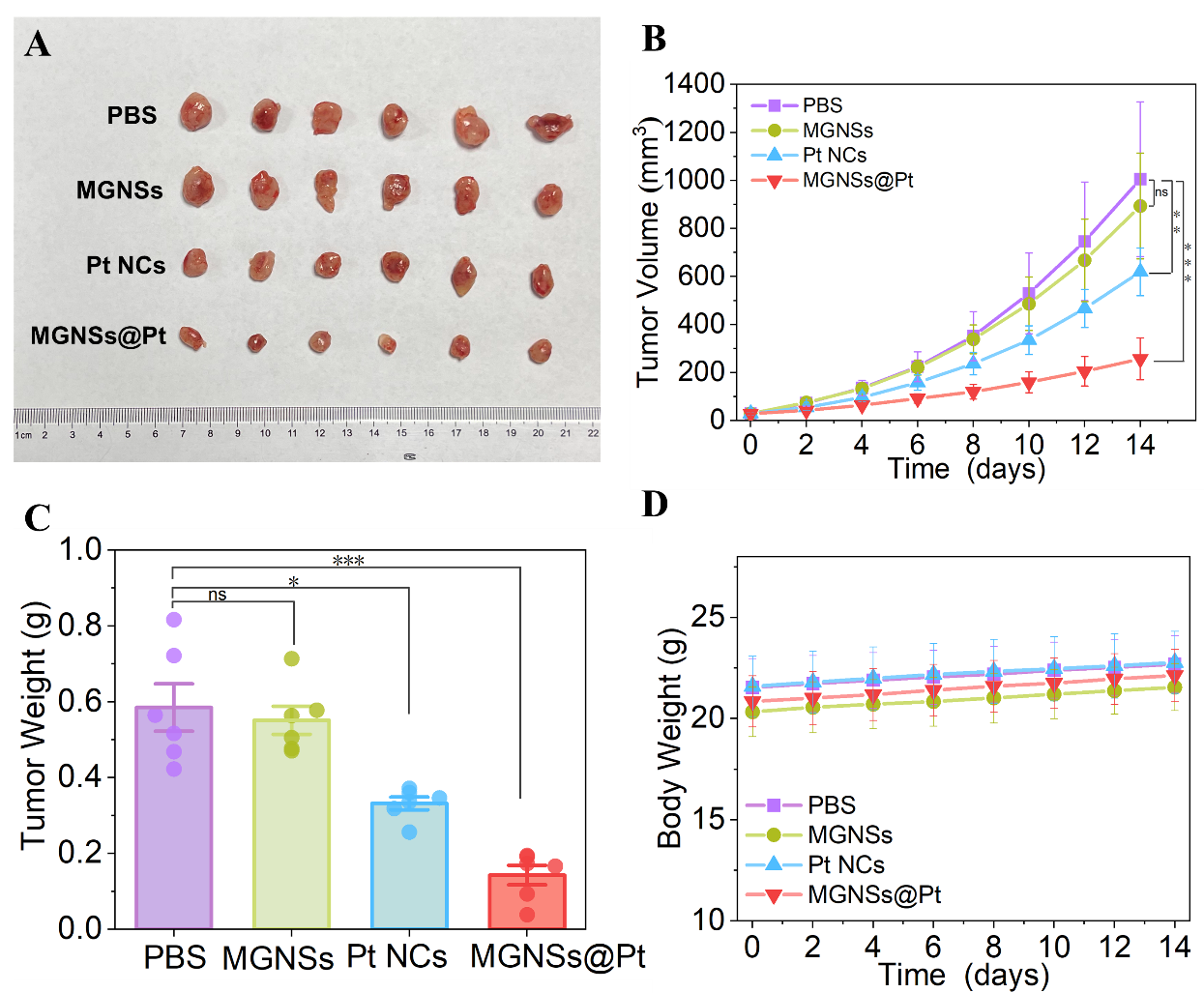


Figure S27**.** Intratumoral injection effect of tumor-bearing mice. (A) The dissected tumors photographs of 4T1-tumor-bearing mice after 14 days of different treatments. (B) Tumor volume and (C) tumor weight of 4T1-tumor-braring mice after different treatment including PBS, MGNSs, Pt NCs and MGNSs@Pt. Nanomaterial concentration: 10 mg kg^-1^. (D) Body weight change of different treatment for 14 days. Data are defined as mean ± SD (n = 6). Statistical significance is assessed by unpaired Student’s two-sided *t*-test and asterisks indicate significant differences (ns：not significant, *p*>0.05, **p* < 0.05, and ****p* < 0.001).


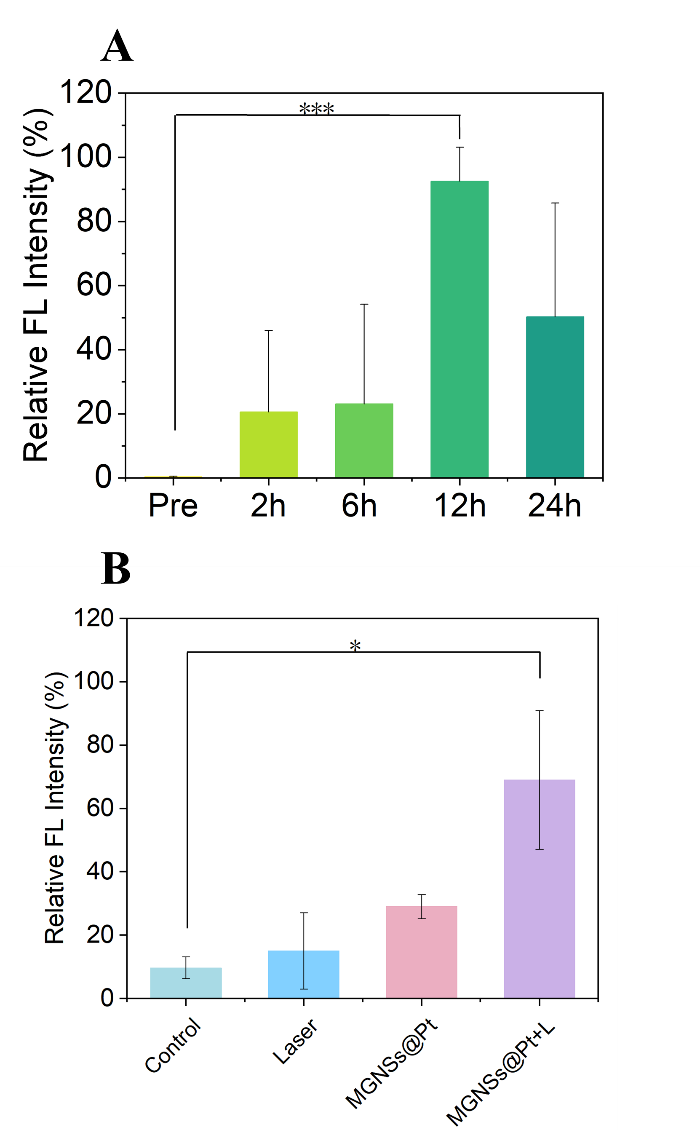


Figure S28. (A) Semi-quantitative analysis of relative fluorescent intensity in real-time biodistribution images of Cy5-labeled MGNSs@Pt. (B) Semi-quantitative analysis of relative green fluorescent intensity in TUNEL images of tumor tissues. Data are defined as mean ± SD (n = 3). Statistical significance is assessed by unpaired Student’s two-sided *t*-test, **p*<0.05, ***p*<0.01, ****p*<0.001.


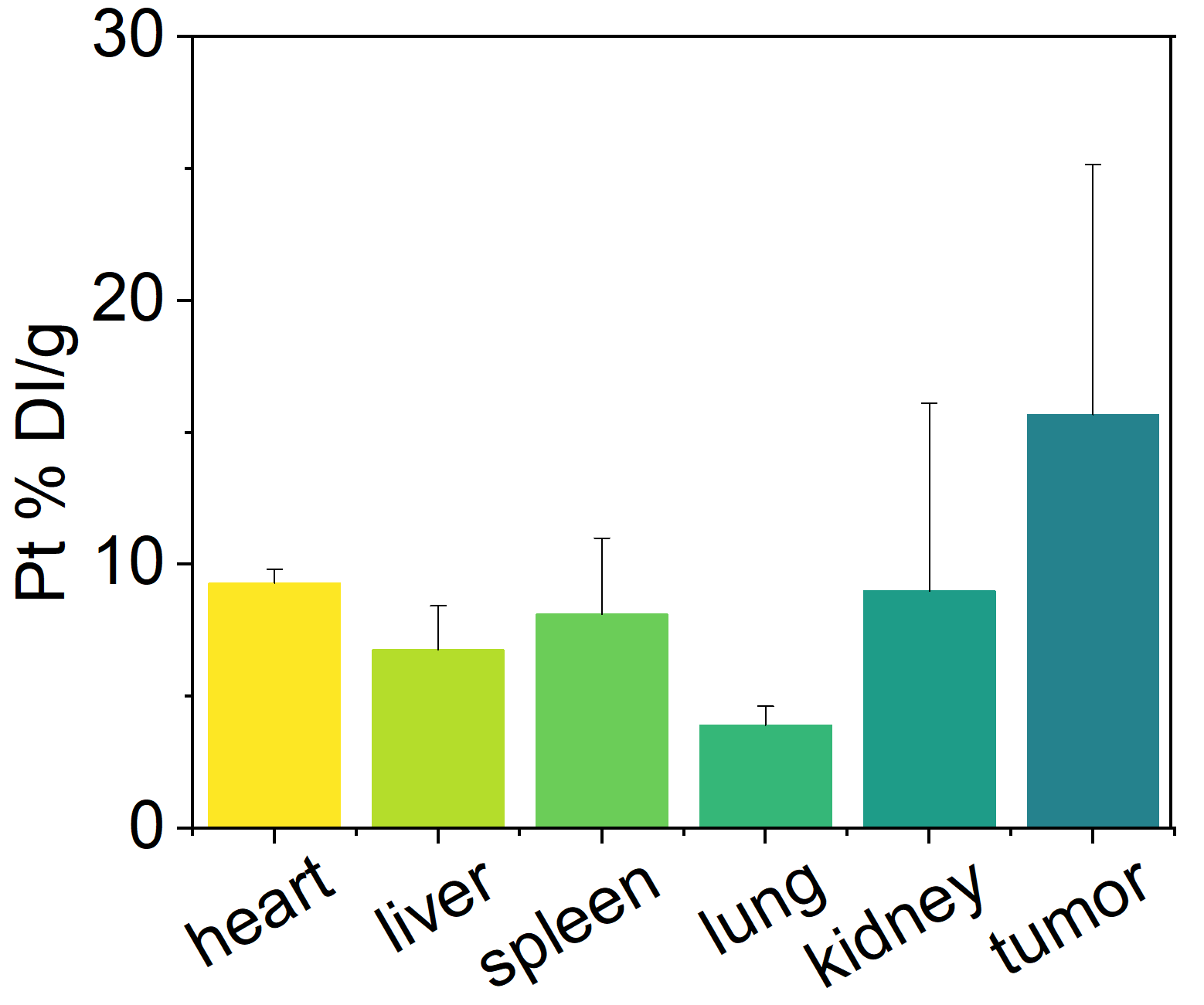


Figure S29. Biodistribution of Pt element after injection of MGNSs@Pt in 4T1 tumor-bearing mice determined by ICP-OES (n = 4).


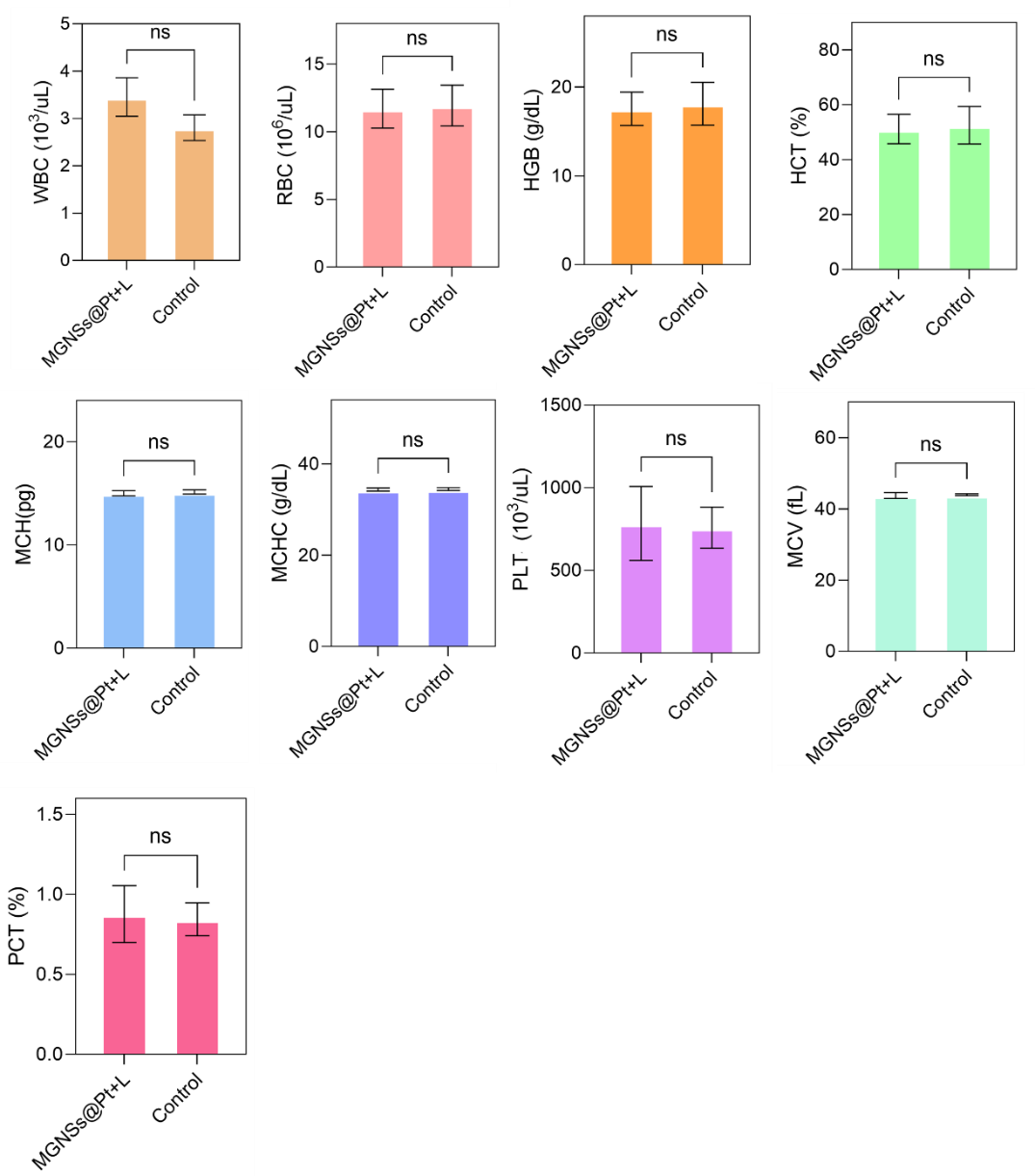


Figure S30. Hematology analysis of BALB/c mice injected with MGNSs@Pt and healthy wild-type (WT) BALB/c control mice. WBC (white blood cells), RBC (red blood cells), HGB (hemoglobin), HCT (hematocrit), MCH (mean corpuscular hemoglobin), MCHC (mean corpuscular hemoglobin concentration), PLT (platelets), MCV (mean corpuscular volume), and PCT (platelet crit) were tested using Sysmex XT-2000i automated hematology analyzer. All data are presented as means ± SD (n = 4 mice per group). Statistical significance is assessed by unpaired Student’s two-sided *t*-test, ns (not significant) *p*>0.05.


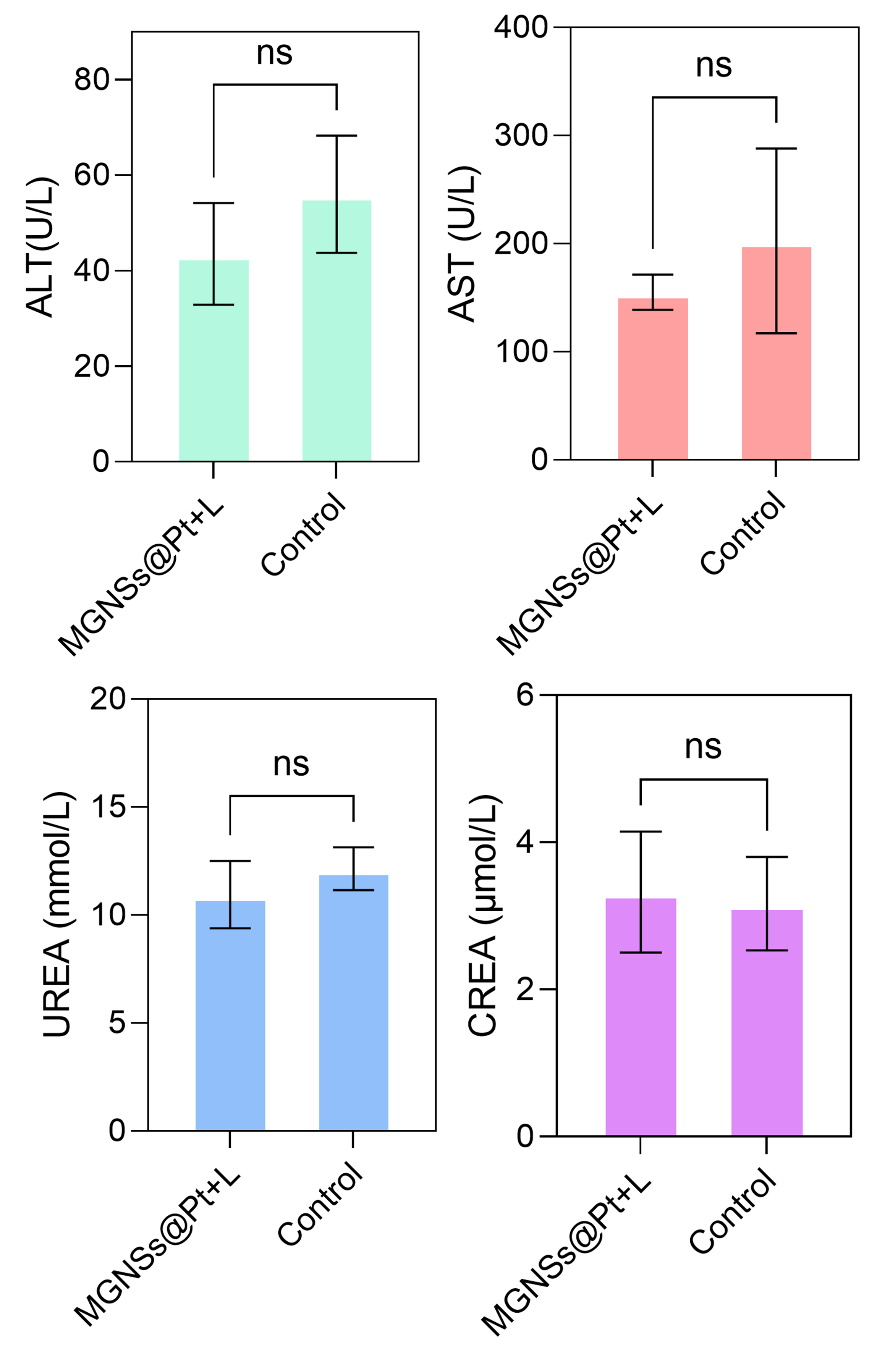


Figure S31. Blood biochemistry of BALB/c mice injected with MGNSs@Pt and healthy wild-type (WT) BALB/c control mice. ALT (alanine aminotransferase), AST (aspartate aminotransferase), UREA (urea or blood urea nitrogen) CRE (creatinine) were tested using an automatic biochemical analyzer BX-3010 (Sysmex). Data are defined as mean ± SD (n = 4). Statistical significance is assessed by unpaired Student’s two-sided *t*-test, ns (not significant) *p*>0.05.

# Supporting Tables

## Table S1. Detailed parameters for synthesis of MGNSs@Pt and the size of Pt NCs in each catalyst.

| **Molar ratio**  **(n_Au_:n_Pt_)** | **MGNSs**  **(μg)** | **AA solution**  **(μL)** | | **H_2_PtCl_6_·6H_2_O**  **(mol L^-1^)** | **Pt size**  **(nm)** | |
| --- | --- | --- | --- | --- | --- | --- |
| 10:1 | 158 | | 80 | 0.002 | | 2.9 |
| 10:2 | 158 | | 80 | 0.004 | | 2.4 |
| 10:5 | 158 | | 80 | 0.01 | | 2.4 |
| 10:10 | 158 | | 80 | 0.02 | | 2.6 |
| 10:20 | 158 | | 80 | 0.04 | | 3.7 |

## Table S2. A summary table with the XPS results of MGNSs@Pt

| **Element** | **Binding Energy (eV)** | **Peak Assignment** | **Atomic** |
| --- | --- | --- | --- |
| Au 4f | 83.3  87.0 | Au 4f_7/2_  Au 4f_5/2_ | 3.5 % |
| Pt 4f | 70.6  73.9 | Pt 4f_7/2_  Pt 4f_5/2_ | 1.58 %  (Pt^2+^ 20.7 %  Pt^0^ 79.3 %) |

.

## Table S3. Au and Pt contents for three catalysts, the particle size of Pt NCs and total size in each catalyst.

| **Catalyst** | **ICP-OES** | | **Pt size**  **(nm)** | **Total size**  **(nm)** |
| --- | --- | --- | --- | --- |
|  | **Au (mg L^-1^)** | **Pt (mg L^-1^)** |  |  |
| MGNSs@Pt | 167.8 | 86.25 | 2.4 | 232.8 |
| GNRs@Pt | 169.4 | 84.9 | 2.5 | 28.8 × 114.8 |
| Pt NCs | / | 84.2 | 2.6 | 2.6 |

## Table S4. Photothermal conversion efficiency of the Pt or Au-related metallic catalyst

| Catalyst | **Photothermal**  **conversion**  **efficiency (η, %)** | **Irradiation wavelength**  **(λ, nm)** | **References** |
| --- | --- | --- | --- |
| AuNFs | 23.9 | 1064 | ^[1]^ |
| Au@Pt nanostructures | 41.3 | 1064 | ^[10]^ |
| Pt@IrSACs/RBC | 38.1 | 1064 | ^[11]^ |
| Pt_50_Sn_50_-PEG BNCs | 38.3 | 808 | ^[12]^ |
| Au_2_Pt-PEG-Ce_6_ | 31.5 | 808 | ^[13]^ |
| Pt-carbon | 39 | 808 | ^[14]^ |
| Platinum nanodots | 46.9 | 808 | ^[15]^ |
| MGNSs@Pt | 43.4 | 1064 | This work |

## Table S5. Comparison of the catalytic efficiencies (K_cat_) of Pt or Au-related metallic catalyst

| Catalyst | **K_cat_(s^-1^)**  (Substrate: TMB) | **References** |
| --- | --- | --- |
| Pt particles | 4.6×10^-2^ | ^[4]^ |
| Au@Pt rods | 1.4×10^4^ | ^[2b]^ |
| Au@Pt rods | 5.7×10^3^ | ^[16]^ |
| Pt cubes | 8.2×10^5^ | ^[17]^ |
| Au@Pt particles | 1.0×10^6^ | ^[18]^ |
| Pt hollow nanodendrites | 1.7×10^-2^ | ^[19]^ |
| Pt NCs | 20.16 | This work |
| GNRs@Pt | 4.74×10^4^ | This work |
| MGNSs@Pt | 1.42×10^6^ | This work |

## Table S6. The model parameters used in COMSOL Multiphysics

| **Model parameters** | |
| --- | --- |
| Initial concentration of H_2_O_2_ | 0.1 mM |
| Initial concentration of ·OH | 0.0 mM |
| Diffusion coefficient of ·OH and H_2_O_2_ | 2.5 × 10^-10^ m^2^ s^-1^ |
| Concentration of MGNSs@Pt | 1.11 × 10^10^ particles/L |
| Concentration of GNRs@Pt | 2.83 × 10^11^ particles/L |
| Surface area of MGNSs@Pt per particle | 7.29 × 10^-13^ m^2^ |
| Surface area of GNRs@Pt per particle | 3.47 × 10^-15^ m^2^ |
| Size of MGNSs@Pt | 215 nm |
| Size of GNRs@Pt | 17 nm in diameter with aspect ratio of 5 |

# References

[1] J. Wang, J. Sun, Y. Wang, T. Chou, Q. Zhang, B. Zhang, L. Ren, H. Wang, *Adv. Funct. Mater.* **2020**, 30, 1908825.

[2] a)M. Broto, M. M. Kaminski, C. Adrianus, N. Kim, R. Greensmith, S. Dissanayake-Perera, A. J. Schubert, X. Tan, H. Kim, A. S. Dighe, J. J. Collins, M. M. Stevens, *Nat. Nanotechnol.* **2022**, 17, 1120; b)W. He, Y. Liu, J. Yuan, J.-J. Yin, X. Wu, X. Hu, K. Zhang, J. Liu, C. Chen, Y. Ji, Y. Guo, *Biomaterials* **2011**, 32, 1139.

[3] X. Ye, C. Zheng, J. Chen, Y. Gao, C. B. Murray, *Nano Lett.* **2013**, 13, 765.

[4] S.-B. He, H.-H. Deng, A.-L. Liu, G.-W. Li, X.-H. Lin, W. Chen, X.-H. Xia, *ChemCatChem* **2014**, 6, 1543.

[5] a)Z. Xi, K. Wei, Q. Wang, M. J. Kim, S. Sun, V. Fung, X. Xia, *J. Am. Chem. Soc.* **2021**, 143, 2660; b)X. Xia, J. Zhang, N. Lu, M. J. Kim, K. Ghale, Y. Xu, E. McKenzie, J. Liu, H. Ye, *ACS Nano* **2015**, 9, 9994.

[6] G. Kresse, J. Furthmüller, *Physical Review B* **1996**, 54, 11169.

[7] a)B. Hammer, L. B. Hansen, J. K. Nørskov, *Physical Review B* **1999**, 59, 7413; b)J. P. Perdew, K. Burke, M. Ernzerhof, *Phys. Rev. Lett.* **1996**, 77, 3865.

[8] a)L. Goerigk, S. Grimme, *Phys. Chem. Chem. Phys.* **2011**, 13, 6670; b)J. Neugebauer, M. Scheffler, *Physical Review B* **1992**, 46, 16067.

[9] J. K. Nørskov, J. Rossmeisl, A. Logadottir, L. Lindqvist, J. R. Kitchin, T. Bligaard, H. Jónsson, *The Journal of Physical Chemistry B* **2004**, 108, 17886.

[10] J. Sun, J. Wang, W. Hu, Y. Wang, Q. Zhang, X. Hu, T. Chou, B. Zhang, C. Gallaro, M. Halloran, L. Liang, L. Ren, H. Wang, *ACS Nano* **2022**, 16, 10711.

[11] J. Cheng, L. Li, D. Jin, Y. Dai, Y. Zhu, J. Zou, M. Liu, W. Yu, J. Yu, Y. Sun, X. Chen, Y. Liu, *Adv. Mater.* **2023**, 35, 2210037.

[12] Y. Zhu, R. Zhao, L. Feng, C. Wang, S. Dong, M. V. Zyuzin, A. Timin, N. Hu, B. Liu, P. Yang, *ACS Nano* **2023**, 17, 6833.

[13] M. Wang, M. Chang, Q. Chen, D. Wang, C. Li, Z. Hou, J. Lin, D. Jin, B. Xing, *Biomaterials* **2020**, 252, 120093.

[14] Y. Yang, D. Zhu, Y. Liu, B. Jiang, W. Jiang, X. Yan, K. Fan, *Nanoscale* **2020**, 12, 13548.

[15] Y. a. Tang, T. Yang, Q. Wang, X. Lv, X. Song, H. Ke, Z. Guo, X. Huang, J. Hu, Z. Li, P. Yang, X. Yang, H. Chen, *Biomaterials* **2018**, 154, 248.

[16] J. Liu, X. Hu, S. Hou, T. Wen, W. Liu, X. Zhu, J.-J. Yin, X. Wu, *Sensors and Actuators B: Chemical* **2012**, 166-167, 708.

[17] H. Ye, Y. Liu, A. Chhabra, E. Lilla, X. Xia, *ChemNanoMat* **2017**, 3, 33.

[18] Z. Gao, H. Ye, D. Tang, J. Tao, S. Habibi, A. Minerick, D. Tang, X. Xia, *Nano Lett.* **2017**, 17, 5572.

[19] C. Ge, R. Wu, Y. Chong, G. Fang, X. Jiang, Y. Pan, C. Chen, J.-J. Yin, *Adv. Funct. Mater.* **2018**, 28, 1801484.
